# Characterization of bending balloon actuators

**DOI:** 10.3389/frobt.2022.991748

**Published:** 2022-09-19

**Authors:** Ung Hyun Ko, Vardhman Kumar, Benjamin Rosen, Shyni Varghese

**Affiliations:** ^1^ Department of Orthopaedics Surgery, Duke University School of Medicine, Duke University, Durham, NC, United States; ^2^ Department of Biomedical Engineering, Pratt School of Engineering, Duke University, Durham, NC, United States; ^3^ Department of Mechanical Engineering and Material Science, Pratt School of Engineering, Duke University, Durham, NC, United States

**Keywords:** soft actuators, pressurized actuators, balloon actuators, bending actuator, balloon actuator

## Abstract

The emerging field of soft robotics often relies on soft actuators powered by pressurized fluids to obtain a variety of movements. Strategic incorporation of soft actuators can greatly increase the degree of freedom of soft robots thereby bestowing them with a range of movements. Balloon actuators are extensively used to achieve various motions such as bending, twisting, and expanding. A detailed understanding of how material properties and architectural designs of balloon actuators influence their motions will greatly enable the application of these soft actuators. In this study, we developed a framework involving experimental and theoretical analyses, including computational analysis, delineating material and geometrical parameters of balloon actuators to their bending motions. Furthermore, we provide a simple analytical model to predict and control the degree of bending of these actuators. The described analytical tool could be used to predict the actuating function of balloon actuators and thereby help generate optimal actuators for functions which require control over the extent and direction of actuation.

## Introduction

Unlike conventional robotics, soft robots are composed of highly compliant materials. The physical characteristics of soft robots allow passive interaction with surroundings to conduct delicate tasks in situations where classic/hard robots might fail, such as in medical assistance, irregular object investigation, and active prosthetics such as grippers (Cianchetti et al., 2018; Schmitt et al., 2018; Shintake et al., 2018; Whitesides, 2018; Jin et al., 2020; Zaidi et al., 2021). Pressurized balloon actuators have emerged as an attractive alternative to electronic-assisted actuators and are increasingly being used to control movements of soft robots (El-Atab et al., 2020). A typical balloon actuator is made of silicone elastomers containing chamber(s) which can be filled with fluids. Introduction of the fluid inflates the chamber, and the expansion pressure generated within the chamber leads to movement of the body resulting in large and fast deformation. Classification of soft actuators and design strategies to achieve different types of actuations have been reviewed in detail (Gorissen et al., 2017; Pagoli et al., 2021; Xavier et al., 2022). Balloon actuators are designed to undergo various movements such as expansion, contraction, twisting, and bending (Fujiwara et al., 2009; Marchese et al., 2015; Schaffner et al., 2018; Balak and Mazumdar, 2020). All of these motions are used at varying extents to endow soft robots with the desired actuation where the type of motion is largely controlled by architectural/geometrical features. For example, the expansion type balloon actuator in one of its design utilizes a bellows-like pattern with cylindrical fluidic channels which is designed to generate rapid linear elongation by concentrating the changes in volume in one direction (Dämmer et al., 2021; Hashem et al., 2021). This type of motion has been utilized in a ring-shaped robot gripper and in an endoscope stabilizer (Gorissen et al., 2018; Schreiber and Manns, 2021). The contracting type of balloon actuator produces axial shortening using radial expansion of the fluid chamber (Dang et al., 2019). This contraction movement generates axial force that can sustain a large magnitude of load and is utilized as an artificial muscle of a soft robot (Verrelst et al., 2005). To achieve twisting type motion, spiral constraints were incorporated within the structure which induces twisting movements of the elastomer body when the chamber inflates (Yan et al., 2018; Hu and Alici, 2020). Surakusumah et al. knitted the balloon actuator with a braided fibrous mat to create a twisting motion and utilized it to control the motion of bronchoscope for crawling the trachea and branches (Surakusumah et al., 2014).

The bending type balloon actuator utilizes a design that has an asymmetric structure involving one or more elastomers (Gorissen et al., 2011; Gorissen et al., 2017). In their simplest form they consist of a single chamber with walls of varying compliances (Xavier et al., 2021). When the chamber is pressurized, expansion occurs on the side with a wall of higher compliance. Upon inflation, the expansion of the balloon structure results in bending force. This bending force produces rapid movement, implementing many types of nature-inspired motions in soft-robotic devices such as those of octopus (Wehner et al., 2016), spider (Ranzani et al., 2018), dragonfly (Kumar et al., 2021), and human finger (Lu and Kim, 2003; Jeong et al., 2005; Kim and Cha, 2020). Various strategies can be used to vary the wall compliance such as changing the geometry, using different materials, or incorporating constraints such as inextensible fibers (fiber-reinforced bending actuators) (Polygerinos et al., 2015). By connecting multiple bending balloon actuators in series, advanced functionalities such as gripping and locomotion can been achieved (Shepherd et al., 2011; Zolfagharian et al., 2020; Pagoli et al., 2021). Most of the balloon actuator assisted soft robotics are focused largely on the type of movement. However, given the relationship between the material and geometrical properties of bending balloon actuator and its actuation, models that can describe and predict the performance of the actuators will expand their applications.

Various models describing the behavior of bending actuators have been reported. Among the analytical models, the Euler–Bernoulli beam theory is the most widely adapted and it treats the bending actuators as ideal beams loaded with moment at its edge and counteracted with the bending stiffness of the beam (Gorissen et al., 2011; Gorissen et al., 2017). However, several limitations of this model have been reported such as its inapplicability at large deformations, inaccuracy due to changes in beam cross-sectional area during actuation, and its use of Young’s modulus, which is not sufficient to capture the complex stress-strain behavior of hyperelastic materials (Gorissen et al., 2017). Coupling of non-linear finite element method (FEM) solvers with hyperelastic material properties have been used to model complex behavior of soft actuators (Gorissen et al., 2017). However, incorrect use of material properties (approximating from literature) can compromise the accuracy of the results (Gorissen et al., 2017; Xavier et al., 2021). Additionally, very few dynamic FEM models of bending actuators have been reported with most models incorporating a fixed load to the actuator and performing a static/quasi-static simulation (Xavier et al., 2021). In this study, we used a single microchannel-based hydraulic bending actuator, a system widely adapted in small-scale soft robotics, to assess the effect of material properties and geometry on bending motion. By using experimentally determined material properties, we performed dynamic FEM analyses to model the bending of the actuator with response to increasing pressure generated by the fluid flow into the microchannel. The bending angle of the balloon actuator with various geometries was characterized as a function of fluidic pressure. In addition, we also varied the mechanical properties of the material used to fabricate different components of the balloon actuator to determine their role on actuation. The computational model was validated experimentally, and a simple analytical model has been proposed to describe the extent of bending of the actuators as a function of fluidic pressure.

## Materials and methods

### Balloon actuator fabrication

**FIGURE 1:**
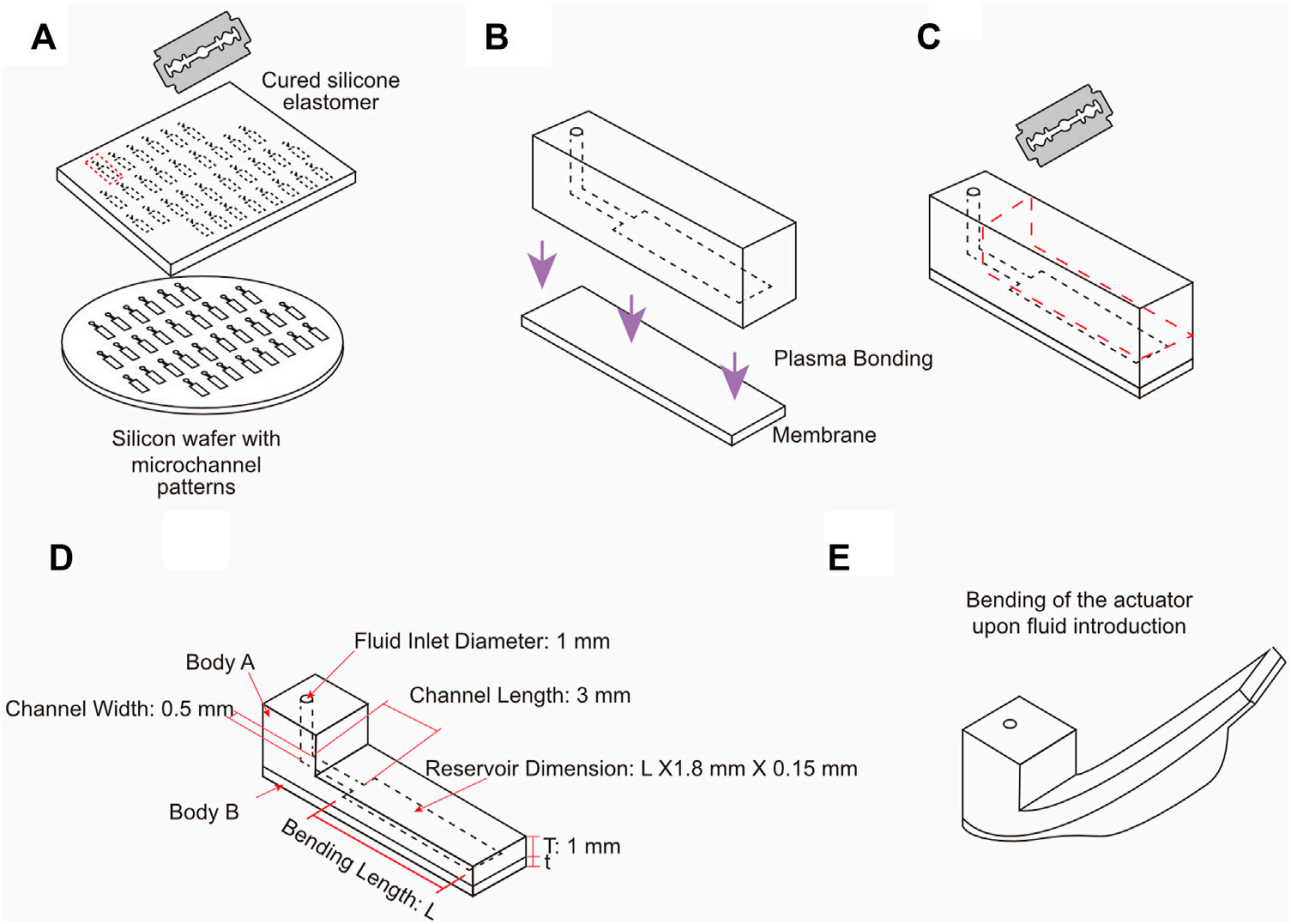
Schematic depicting the process of balloon actuator fabrication. **(A)** Silicon wafers patterned with microchannels were used as molds for soft lithography of Body A. **(B)** Inlet was punched into Body A which was then bonded to Body B (membrane prepared using spin coating) using oxygen plasma **(C)** Body A was trimmed to maintain a thickness of 1 mm over the microchannel of all actuators. **(D)** Dimensions of different parts of the actuator. **(E)** Bending of the actuator upon fluid introduction.

The balloon actuator was prepared by plasma bonding of two independent elastomeric bodies—body A and body B—with different mechanical properties and dimensions. The elastomeric body A contained a microchannel connected to a rectangular chamber whose length was varied to create balloons with different expansion area. The microchannel was designed as a straight channel with dimensions of 0.5 mm × 3 mm using AutoCAD software and connected to the rectangular chamber without a fluid outlet. The microchannel and chamber were created by soft lithography (Figure 1A) using patterned silicon wafer (100 mm, (1 0 0), boron-doped, p-type, ID: 452, University Wafer). Patterns with a height of 150 μm were etched using SU-8-100 photoresist (MicroChem, Inc.) according to the manufacturer’s instructions. Polydimethylsiloxane (PDMS) (Sylgard 184) was cured at 60°C for at least 2 h and released from the patterned wafer. After curing, a microchannel inlet was created using a 1 mm diameter biopsy punch (Miltex, Inc.). The body B, an expandable membrane, was fabricated by spin-coated PDMS or Ecoflex 00-30 of desired thickness. The bonding between the body A and the body B was achieved by using oxygen plasma (K1050X, Quorum Technologies, Ltd.) (Figure 1B) at 50 W for 30 s and the body A was trimmed to maintain 1 mm thickness above the microchannel (Figures 1C,D). The bonded structures were stored at 60°C until used for actuation by means of fluid introductions (Figure 1E).

### Uniaxial tensile testing

Hyperelastic material properties of PDMS were determined by uniaxial tensile testing using ElectroForce 3220 Series III (TA Instruments, Inc.). The ASTM (American Society for Testing Materials) Type V dumbbell specimen of 4 mm thickness was used with a 225 N load cell. The stress-strain measurements were carried out at 0.25 mm/min strain rate. A stress-strain curve was fitted using a two-term Ogden model (Eq. 1) to acquire the Ogden parameters.

$$\sigma \left(\lambda \right)=\frac{1}{\lambda^{2}}\cdot \left(\mu_{1}\cdot \lambda^{\alpha_{1}}+\mu_{2}\cdot \lambda^{\alpha_{2}}-\mu_{1}\cdot \lambda^{\left(-\frac{1}{2}\right)\alpha_{1}}-\mu_{2}\cdot \lambda^{\left(-\frac{1}{2}\right)\alpha_{2}}\right)$$ (1)

Where, λ is the principle stretch and α_1_, α_2_, μ_1_, and  μ_2_ are material constants.

The Ogden parameters $\mu_{1}$ , $\mu_{2}$ , $\alpha_{1}$ , and $\alpha_{2}$ for PDMS were calculated through curve-fitting (Table 1 and Supplementary Figure S1). Ecoflex 00-30 Ogden parameters were obtained from our previous study (Kumar et al., 2021). These parameters were used for COMSOL simulation.

**TABLE 1:** Two-term Ogden parameters of Ecoflex 00-30, and PDMS with 1:10, 1:15 ratio of base to curing agent.

		$\mu_{1}$ (kPa)	$\mu_{2}$ (kPa)	$\alpha_{1}$	$\alpha_{2}$
Ecoflex 00-30		1.241	7.879 × 10^−9^	3.034	13.02
PDMS	1:10	1.111 × 10^−22^	21.66	0	3.268
	1:15	0	9.417	1.176	3.299

### COMSOL simulation

3D numerical simulations using COMSOL 5.3a or 5.5 were performed to characterize the balloon actuator bending angle with different chamber dimensions and mechanical properties. The balloon actuator developed in the simulation was composed of a flexible body (similar to body A) and an expandable membrane (similar to body B). The flexible body and expandable membrane dimensions were designed to be 3 mm width × 12 mm length × 1 mm height, and 3 mm width × 12 mm length with membrane thickness as variable t, respectively. The rectangular chamber between the body and the membrane created a fluidic domain having a chamber with length L (input variable) × 1.8 mm width × 150 μm height. It should be noted that the balloon expansion is constrained to the rectangular chamber, and hence the inlet channel and tubing part was removed in the computational domain to minimize the computational cost. A 2.5 μl/s fluid flow was generated in the rectangular chamber’s right end to model the fluid entry through the inlet of the microchannel. The following boundary conditions were used:

Fluid domain (Inside the chamber):

Inlet: volume flow rate $V_{0}$ ;

All other walls: no slip with u⋅n=0 ;

Initial conditions: u=0 and p=0.

Solid domain (flexible body and expandable membrane):

Right ends: x=0 ;

All other outer boundaries: free boundaries and zero stress.

Initial conditions: x=0 and $\frac{\partial x}{\partial t}=0.$

The moving mesh was applied for the fluid domain, and the physics of the fluid domain were calculated using the laminar flow module. The physics of the solid domain were solved using a solid mechanics module with the experimentally obtained Ogden parameters. The fluid-structure interaction was applied to the coupled interfaces between the fluid and solid domain. All other estimated values of physical parameters are summarized in Supplementary Table S1 and a detailed COMSOL report is provided in Supplementary Information.

### Experimental validation of bending angle

To experimentally validate the COMSOL simulation findings of the bending movement, various balloon actuators were prepared. Experiments were carried out to investigate differences in bending motion as a result of changes in chamber length, membrane thickness, and body/membrane materials with different mechanical properties. The experimental groups are summarized in Table 2.

**TABLE 2:** Balloon actuator design for each experimental group.

	Chamber length variation	Membrane thickness variation	Body/membrane materials variation
Body Materials	PDMS (1:10)	PDMS (1:10)	PDMS (1:10), PDMS (1:15), Ecoflex 00-30
Chamber Length	4.1, 7.1, 11.1, 15.1 mm	7.1 mm	7.1 mm
Membrane Materials	Ecoflex 00-30	Ecoflex 00-30	PDMS (1:15)
			Ecoflex 00-30
Membrane Thickness	0.5 mm	0.25, 0.5, 1 mm	0.5 mm

The balloon expansion was enabled using a syringe pump (Harvard Apparatus, Inc.) with a fluid feeding at rate of 2.5 μl/s through a microchannel inlet. The corresponding actuations were video recorded for 10 s and quantified every 1 s using ImageJ software (ImageJ software, NIH) (Supplementary Figure S2).

### Bending force measurement

The bending force of the balloon actuator was quantified by force equilibrium using a custom designed experimental approach. The balloon actuator and an elastomeric rubber band (6.35 cm × 0.15 cm) were connected. The actuator prior to fluid introduction (i.e., unactuated) and the unstretched rubber band were initially maintained in a straight line. A bending force was generated by a fluid flow at a feed rate of 2.5 μl/s through the microchannel inlet, and the stretch length was measured by pulling the rubber band every second. Stretch force was estimated by using Hooke’s law (Eq. 2).

$$\left|F_{\text{bending}}\right|=kx$$ (2)

Where, k is the elastic constant of rubber and x is the change in length of the rubber band. The constant k was quantified and was shown in Supplementary Figure S3.

## Results and discussion

### The operation principles of bending type balloon actuator

**FIGURE 2:**
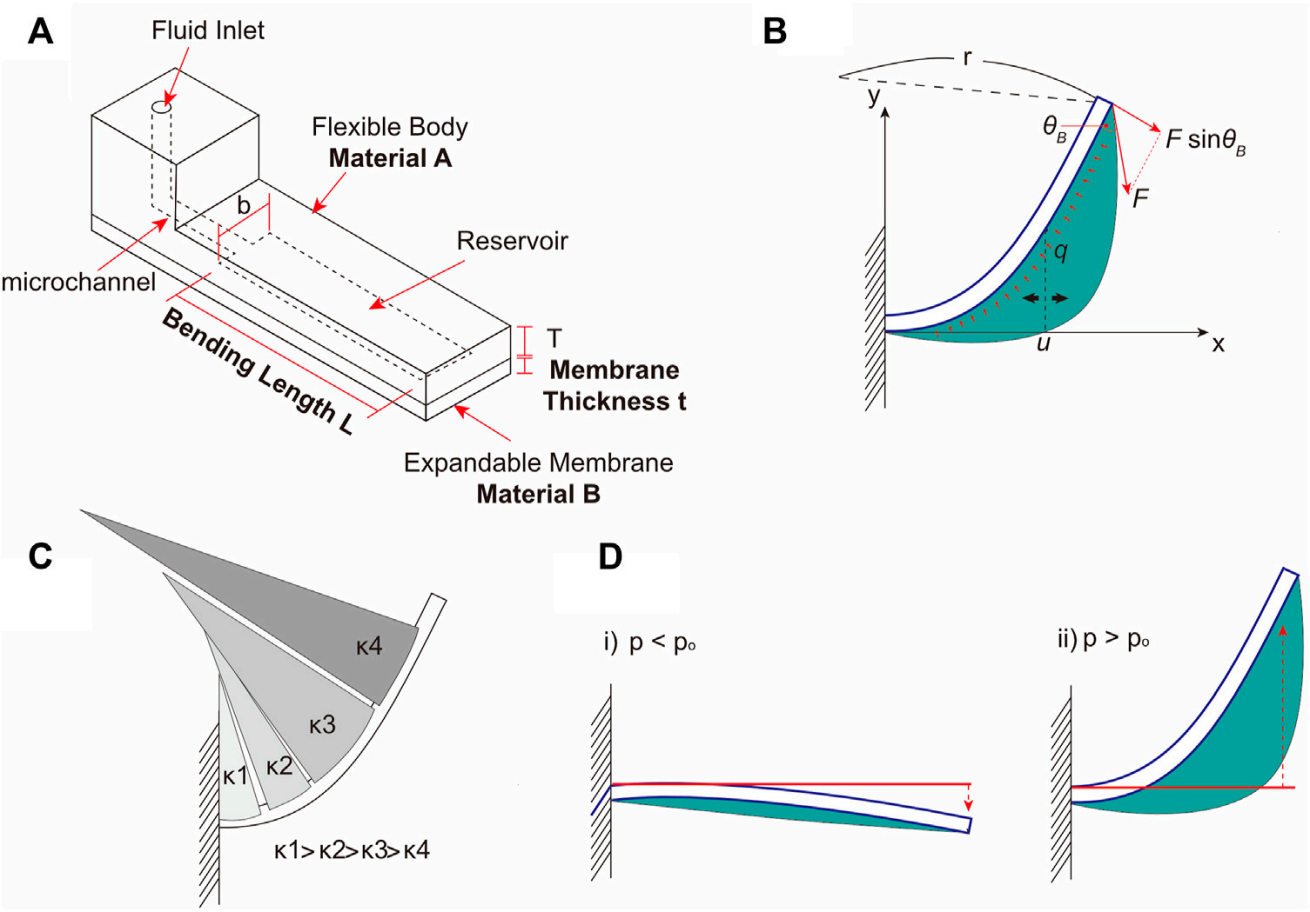
The design and working of balloon actuator. **(A)** Schematic of balloon actuator showing various components – Chamber length L, membrane thickness t, Materials A and B are the materials constituting the Body A and Body B, respectively. **(B)** Free body diagram of balloon actuator. The thick blue line indicates the flexible body, and the green area indicates the surface of the expandable membrane. Red arrows represent the forces acting on a flexible body. **(C)** Bending shape of the balloon actuator according to the change in curvature. **(D)** Illustration of two bending cases during membrane expansion. The bending direction of the body is decided by the degree of membrane expansion.

The bending motion of the balloon actuator was produced by an asymmetric deflection of the two silicone elastomer layers, that are bonded to each other, with differential mechanical properties and thicknesses. Figure 2A shows the schematic of the balloon actuator that was used for the theoretical and experimental analyses. When fluid is injected through a microfluidic channel, the expandable membrane (body B) is inflated by a hydraulic pressure, creating a balloon in the chamber. With the membrane expansion, the flexible body A is subjected to a hydraulic pressure from the fluid inside the balloon and a simultaneous pulling force from the elasticity of the membrane (*i.e.*, body B) (Figure 2B). Gorissen *et al.* (Gorissen et al., 2011) demonstrated that the flexible body can be modeled as a cantilever beam, and by the Euler-Bernoulli beam equation, the bending curvature can be calculated as follows,

$$\frac{M\left(u\right)}{E_{B}I_{B}}=\kappa \left(u\right)$$ (3)

Where u is the *x*-axis value between 0 to L of the chamber, M(u) is the local bending moment and membrane pulling force F , $E_{B}$ is the Young’s modulus of the flexible body (i.e, body A), $I_{B}$ is the second moment of inertia of the flexible body A and κ(u) is the local curvature.

From Eq. 3 and Figure 2B, as the value of u increases, the length of moment arm decreases, and thereby the value of M(u) decreases. Therefore, the local curvature of the flexible body A decreases as u increases and becomes 0 at u=L . The bending shape can be estimated as shown in Figure 2C. In addition, by the 2D Laplace equation of expandable membrane (i.e., body B), p and F can be written as follows,

$$p=\frac{\gamma }{r},F=\gamma b$$ (4)

where b is the width of the chamber, p is the pressure in the actuator, r the radius of membrane curvature, and γ the surface tension.

It has been shown previously that when the balloon is small and the pressure inside the balloon is lower than a certain transition pressure, (p < p_o_, with p_o_ being the transition pressure), the flexible body (i. e., body A) bends towards the balloon (Konishi et al., 2006; Gorissen et al., 2011). However, when the balloon is large and the transition pressure is exceeded (p > p_o_), then the flexible body (i.e., body A) bends towards the opposite side of the balloon (Figure 2D). Herein, we focused on p > p_o_ for all experimental conditions because the expandable membrane (i.e., body B) inflates rapidly, and the duration of p < p_o_ can be considered negligible.

### Effect of chamber length on bending angle

Balloon actuators with varying chamber lengths (L), 4.1, 5.1, 7.1, 9.1, 11.1, 13.1, 15.1, and 17.1 mm, were simulated to assess the bending angle changes. From the analyses, we have arbitrarily chosen a balloon actuator with 7.1 mm chamber length (L), 1 mm thickness of the body A (T), 0.5 mm thickness of the body B (t), and 1.8 mm chamber width (b) as a standard and compared against for all other experimental groups (Table 3).

**TABLE 3:** Geometrical description and material composition of the balloon actuator that was set as the standard.

Material A	Material B	L (mm)	T	t (mm)	b (mm)
PDMS (1:10)	Ecoflex 00-30	7.1	1	0.5	1.8

**FIGURE 3:**
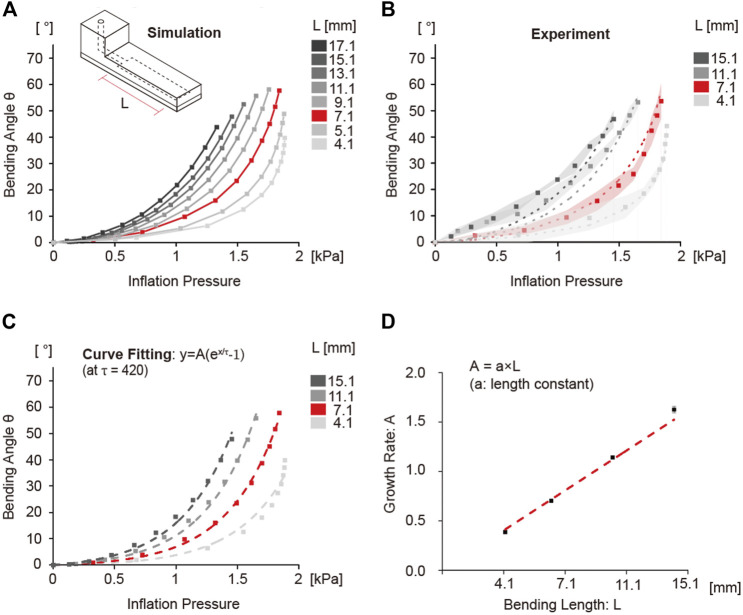
Simulation and experimental results of bending angle change as a function of chamber length. **(A)** Simulation plot showing the bending angle as a function of inflation pressure. The difference in the bending angle was confirmed by gradually increasing the chamber length from 4.1 to 17.1 mm. Curve for 7.1 mm shown in red was considered the standard control condition. **(B)** Experimental results of bending angle with 4.1, 7.1, 11.1, 15.1 mm chamber length. Dots indicate the average values of bending angle, and the shaded area represents the standard deviation of each data point. Dotted lines are the simulation results from **(A)**. **(C)** Curve fitting analysis results using the exponential growth model. The dashed line indicates the fitting curve graph using estimated variables. **(D)** Change of amplification constant A according to change in bending length. The constant A showed a linearly increasing relationship with chamber length.

As suggested by the COMSOL solution, for a fixed pressure, increasing the chamber length resulted in an increased bending angle (Figure 3A). This is attributed to the increase in moment arm with increasing chamber length, which will result in greater moment and curvature (Eq. 3) at the same inflation pressure. Beyond a chamber length of 7.1 mm, an increase in input pressure had a smaller effect on bending angle. This is because with increasing chamber length more fluid is required to generate the same inflation pressure compared to chambers with lower chamber length, resulting in slower response. The simulation results were verified by experiments. As shown in Figure 3B, all the balloon actuators with varying chamber lengths showed similar bending behavior as the simulation.

The curves of the bending angle with different inflation pressures were mathematically modeled to characterize the effect of chamber length on bending angle by using an exponential function (Eq. 5).

$$y=A\left(e^{\frac{x}{\tau }}-1\right)$$ (5)

where y is the bending angle, x the inflation pressure, A the amplification constant, and τ the pressure constant. The curve of the bending angle with various chamber lengths were fitted with a constant τ value of 420 to demonstrate the effects of increasing chamber length on the amplification constant A. The initial values of τ and A were estimated from the exponential function curve fitting of the standard curve of the balloon actuator without any constraints. The amplification constant A in Table 4 was calculated from the curve fitting data in Figure 3C.

**TABLE 4:** The amplification constants A for balloon actuators with different chamber lengths were calculated by the exponential growth curve fitting with high R^2^ values.

	L: 4.1 mm	L: 7.1 mm	L: 11.1 mm	L: 15.1 mm
A	0.387 ± 0.014	0.704 ± 0.007	1.142 ± 0.016	1.626 ± 0.032
R^2^	0.959	0.998	0.996	0.992

The pressure constant τ was fixed instead of the amplification constant A because the curve fitting for the latter resulted in poorer fit with the exponential growth model as shown in Supplementary Table S2. For a fixed τ value, the amplification constant A increased proportionally with the chamber length (L), and the values of A can be predicted from the slope of the linear relationship between A and L. (Figure 3D). We defined this slope as the length constant “a,” whose value was found to be 0.101 ± 0.002 with an R^2^ = 0.991. In contrast, if A values were fixed at 0.65, the R^2^ values for curve fitting data were lower (Supplementary Table S2). These results suggest that with increasing the chamber length the amplification constant increases, which results in vertical amplification of the bending angle curves.

### Effect of membrane thickness on bending angle

**FIGURE 4:**
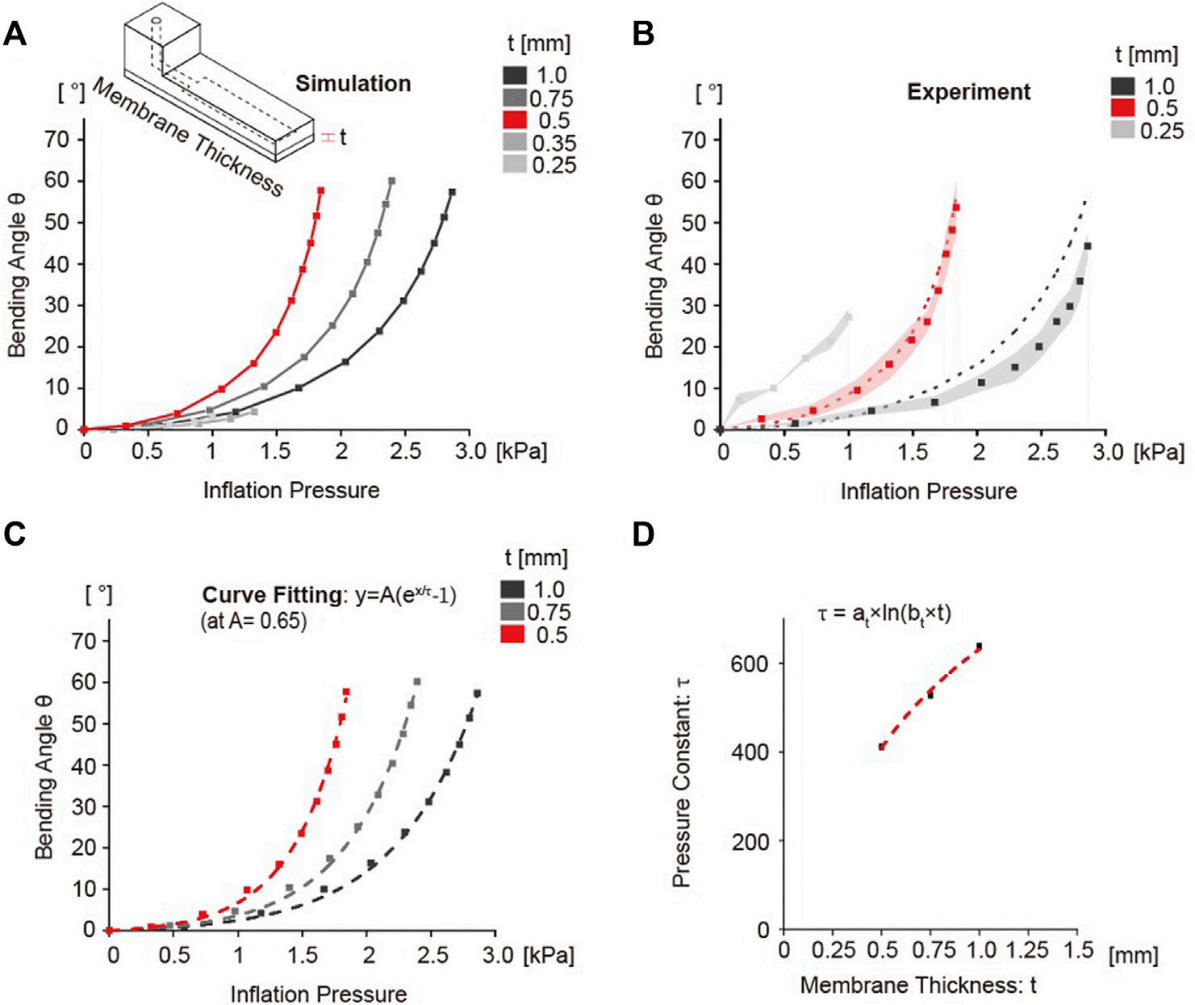
Simulation and experimental results of bending angle as a function of membrane thickness. **(A)** Simulation graph showing the bending angle as a function of inflation pressure change. The difference in the bending angle was confirmed by gradually increasing the membrane thickness from 0.25 to 1.0 mm. 0.5 mm curve shown in red was considered the standard control condition. **(B)** Experimental results of bending angle with 0.25, 0.5, 1.0 mm. Dots indicates the average values of bending angle, and the shaded area represented the standard deviation of each data point. Dotted lines are the simulation results from **(A)**. **(C)** Curve fitting analysis results using the exponential growth model. The dashed line indicates the fitting curve graph using estimated variables. **(D)** Change of pressure constant τ according to change in membrane thickness. The constant τ showed a logarithmically increasing relationship with membrane thickness.

The balloon actuators with varying membrane thickness of body B, 0.5, 0.75, or 1.0 mm, were simulated to assess its effect on bending angle. All variables except membrane thickness were fixed as the standard as described in Table 3. As expected, we found that thicker membranes required more pressure to generate the same bending angle compared to those with thinner membranes (Figure 4A); this resulted in a shifting of the bending curve along the *x*-axis as the membrane thickness increased. These results were verified experimentally, which showed the same trend with similar values (Figure 4B).

When the thickness of the membrane was decreased below 0.5 mm, the formation of a node in the middle of the chamber was observed and the expanded balloon separated into two smaller balloons. Corresponding experimental analyses validated the stimulation results (Supplementary Figure S4). This is likely due to inability of the thinner membranes to withstand large strains as the formation of two separate balloons results in smaller deformation. The balloon actuators with membrane thickness that leads to formation of two small balloons displayed minimal bending (Figure 4A). Furthermore, thinner membranes were also more vulnerable to ruptures during balloon expansion. Balloon actuators exhibiting this phenomenon could compromise functionality when used in soft robotics. Hence, we used 0.5 mm as the lower limit for the membrane thickness and further analysis was performed only for membrane thickness >0.5 mm.

The simulation data for various membrane thicknesses were fitted by using an exponential growth function with the fixed amplification constant A of 0.65 (Figure 4C). The initial value of A was estimated from the exponential function curve fitting of the standard without any constraints. From the results, the pressure constants τ were estimated as reported in Table 5. The shifting of the bending curve along the *x*-axis with varying membrane thickness was captured in the exponential growth model when the pressure constant τ was increased.

**TABLE 5:** The pressure constant τ in different membrane thicknesses of the balloon actuators were calculated by exponential growth curve fitting with high R^2^ values.

	0.5 mm	0.75 mm	1 mm
τ	412.2 ± 0.902	528.6 ± 1.07	638.3 ± 1.17
R^2^	0.998	0.998	0.998

The pressure constant τ was found to increase with membrane thickness. Because the τ value must be an infinite value for 0 mm thickness, we employed the natural logarithm model (Eq. 6) to predict the τ value when the membrane thicknesses were changed (Figure 4D).

$$\tau =a_{t}\times \ln\left(b_{t}\times t\right)$$ (6)

where t is the thickness of the membrane.

The a_t_ and b_t_ values for the curve in Figure 4D were 731.2 ± 57.11 and 7.179 ± 0.896, respectively, with an R^2^ of 0.988. In contrast, if τ value was fixed at 420, the R^2^ value of curve fitting was lower (Supplementary Table S3), suggesting a smaller influence of amplification constant A in predicting the relationship between membrane thickness and curve of bending angle.

### Effect of mechanical properties of the two layers on bending angle

**FIGURE 5:**
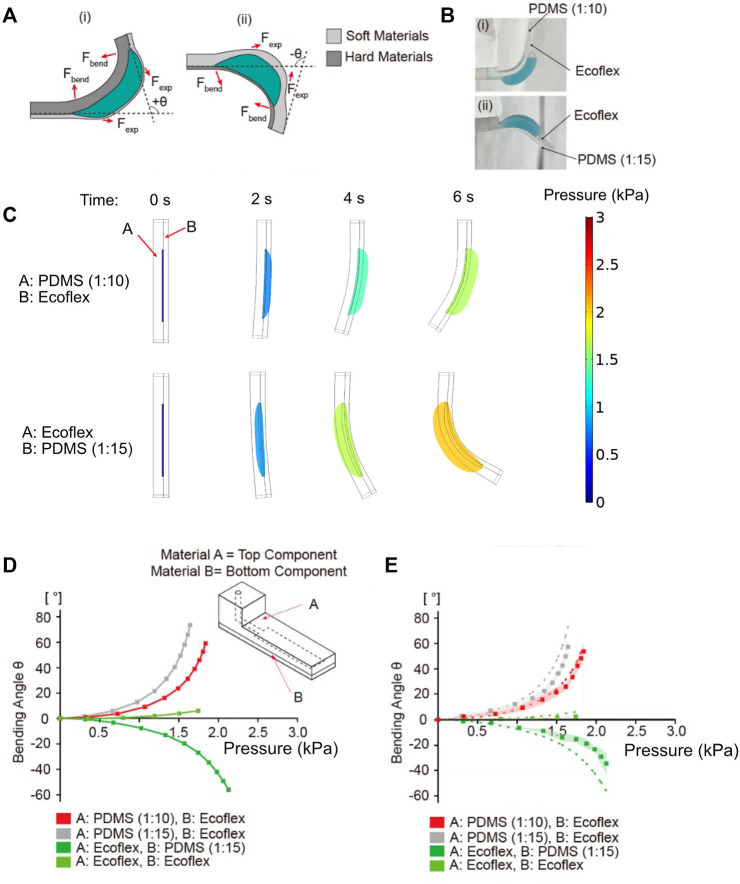
Simulation and experimental results for bending angle dependence on the materials A and B. **(A)** Change in bending motion due to difference in stiffness of materials A and B. **(B)** The experimental results of bending motion according to the stiffness of materials A and B: (i) When material B (expandable membrane) was softer than material A, the balloon actuator bent upwards and (ii) when material A was softer than material B, the flexible body (material A) underwent balloon expansion, causing reversed bending downward. **(C)** Images from COMSOL results showing the shape of actuators and the generated pressure at different time points. **(D)** Simulation results showing the bending angle as a function of inflation pressure. Various materials A and B of balloon actuator were tested (PDMS 1:10, 1:15, Ecoflex 00-30) to verify the effect of material properties on bending angle. **(E)** Experimental results of bending angle with different materials A and B (PDMS 1:10, 1:15, Ecoflex 00-30). Dots indicate the average values of bending angle, and the shaded area represents the standard deviation of each data point. Dotted lines are the simulation results from **(D)**.

Next, we studied the role of mechanical properties in determining the bending actuation. We tested various combination of materials A and B for the two layers of the balloon actuator. Among the two layers, it is the softer layer that inflates and forms the balloon. This expansion causes the direction of expansion force F_exp_ to induce an asymmetric bending towards the stiffer layer side (Figure 5A). This control over direction was verified experimentally (Figure 5B). If PDMS (1:10) and Ecoflex 00-30 were used for Material A and B for body A and B, respectively, the bending occurred toward Material A (PDMS 1:10). In contrast, if Ecoflex 00-30 and PDMS (1:15) were used as Material A and B for body A and B, respectively, the bending occurred towards Material B (PDMS 1:15). The Young’s moduli of PDMS (1:10), PDMS (1:15) and Ecoflex are approximately 2 MPa, 1 MPa and 0.125 MPa respectively (Park et al., 2010; Wang et al., 2014; Shintake et al., 2017).

To verify the effect of mechanical properties on bending angle and direction of bending, we simulated the bending angle using PDMS with 1:10 and 1:15 ratios of base to curing agent and Ecoflex 00-30 as combinations for materials A and B (Figures 5C,D). As evident from the results, the bending angle was increased when PDMS (1:15) was used as a material for body A compared to the standard. Although the membrane generates the same F_exp_ using Ecoflex 00-30, the small Young’s modulus $E_{B}$ of PDMS (1:15) caused an increased curvature according to Eq. 3. However, when both bodies A and B were generated from Ecoflex 00-30 with similar Young’s moduli, a minimum bending towards the body with higher thickness was observed. These results suggest that the bending angle was not only regulated by individual stiffnesses of body A and B, but also the difference in stiffness between the two. As expected, the bending direction was changed when Ecoflex 00-30 and PDMS (1:15) were used for body A and B, respectively. Results from the stimulation were verified experimentally which showed a good agreement (Figure 5E).

### Quantifying the bending force as a function of chamber length

**FIGURE 6:**
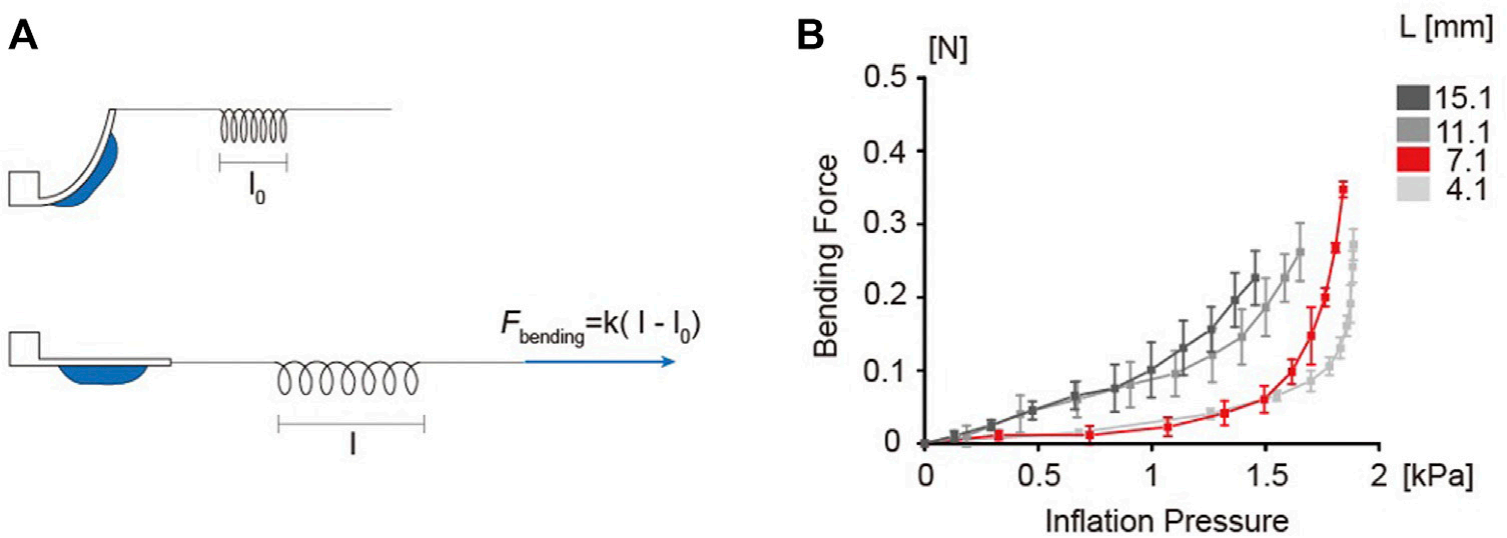
Experimental setup for bending force measurement. **(A)** Illustration of force measurement method using a rubber band. The rubber band is described as a spring in the figure. The rubber band was pulled to estimate the bending force until the flexible body became parallel with the pulling direction. **(B)** Bending force curves as a function of inflation pressure. 4.1, 7.1, 11.1 and 15.1 mm chamber lengths were tested.

The balloon actuators with different chamber lengths (L) 4.1, 7.1, 11.1, 15.1 mm were used to quantify the bending force. All variables except chamber length were fixed same as the standard (Table 3). As described in Materials and Methods, a custom designed equilibrium system was used to estimate the bending force (Figure 6A). The results showed that the bending force increased exponentially with increase in inflation pressure (Figure 6B). The bending force curve showed a trend similar to the bending angle curve, with increased bending force correlating with higher pressure. Such analysis is crucial in applications that require the actuators to perform functions such as pulling or lifting.

## Conclusion

In this study, we used a comprehensive theoretical analyses and experiments to describe the behavior of bending type balloon actuator and studied the effect of mechanical properties of the material and geometrical parameters of the actuator on its performance. Specifically, we found that increasing the length of the chamber amplified the bending angle and an increase in membrane thickness caused smaller bending angles, which were explained by an exponential growth model. Our analyses showed a key influence of the mechanical properties of the materials and its differences on the degree and direction of the bending angle of the actuator. Furthermore, employing a simple, customized setup, we have determined the force applied by the actuators during their function. In this proof-of-concept study, we used chamber length as a parameter to estimate the forces. Similarly, other design parameters can be varied to study their effect on the force which needs to be verified in future studies. While the analytical model described here utilizes an exponential growth model, it only validates functional actuators and does not account for the upper limit or failure of actuation. Nonetheless, the analytical model described in this study could serve as an important tool in designing soft robotics with controlled and versatile bending movements. Additionally, rather than identifying a universally optimum actuator, our approach allows selection of application-specific design to achieve targeted function (for example, higher bending angle, lower response times, directional bending etc). While actuators with only a single chamber have been analyzed in the current study, the framework described here can be extended to study actuators with multiple chambers in series or parallel as used in several soft robots (Shepherd et al., 2011; Maruthavanan et al., 2021). Such theoretical and predictive analysis can help reduce experimental time and resources associated with selecting optimal soft actuators for targeted functions such as designing a gripper with individual “fingers” requiring different extent of actuations.

## Data Availability

The raw data supporting the conclusions of this article will be made available by the authors, without undue reservation.
