# Supplementary material for: Characterization of bending balloon actuators
Source: Front Robot AI. 2022 Sep 19;9:991748. doi: 10.3389/frobt.2022.991748 (PMC9528995; doi:10.3389/frobt.2022.991748)
Supplement: Supplementary file 1 [file DataSheet1.docx]

Supplementary Material

**Supplementary Figures**

**Figure S1.**

**
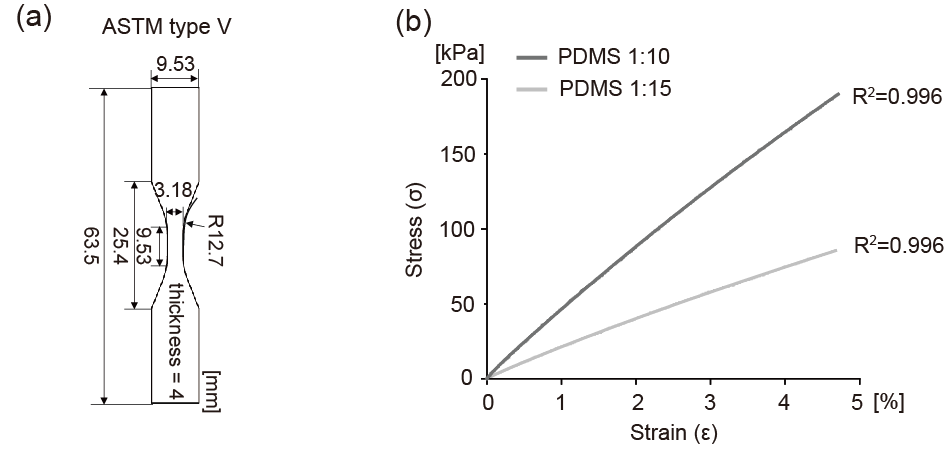
**

**Figure S3 Estimating the hyperelastic properties of PDMS 1:10, 1:15 (a) The PDMS block was manufactured to ASTM type V shape to perform the uniaxial tensile test. (b) The hyperelastic properties of PDMS were calculated by analyzing the stress-strain curve. The stress-strain curve was fitted by using the two-term Ogden model.**

**Figure S1.** Estimating the hyperelastic properties of PDMS 1:10, 1:15 (a) The PDMS block was manufactured to ASTM type V shape to perform the uniaxial tensile test. (b) The hyperelastic properties of PDMS were calculated by analyzing the stress-strain curve. The stress-strain curve was fitted by using the two-term Ogden model.

**Figure S2.**

**
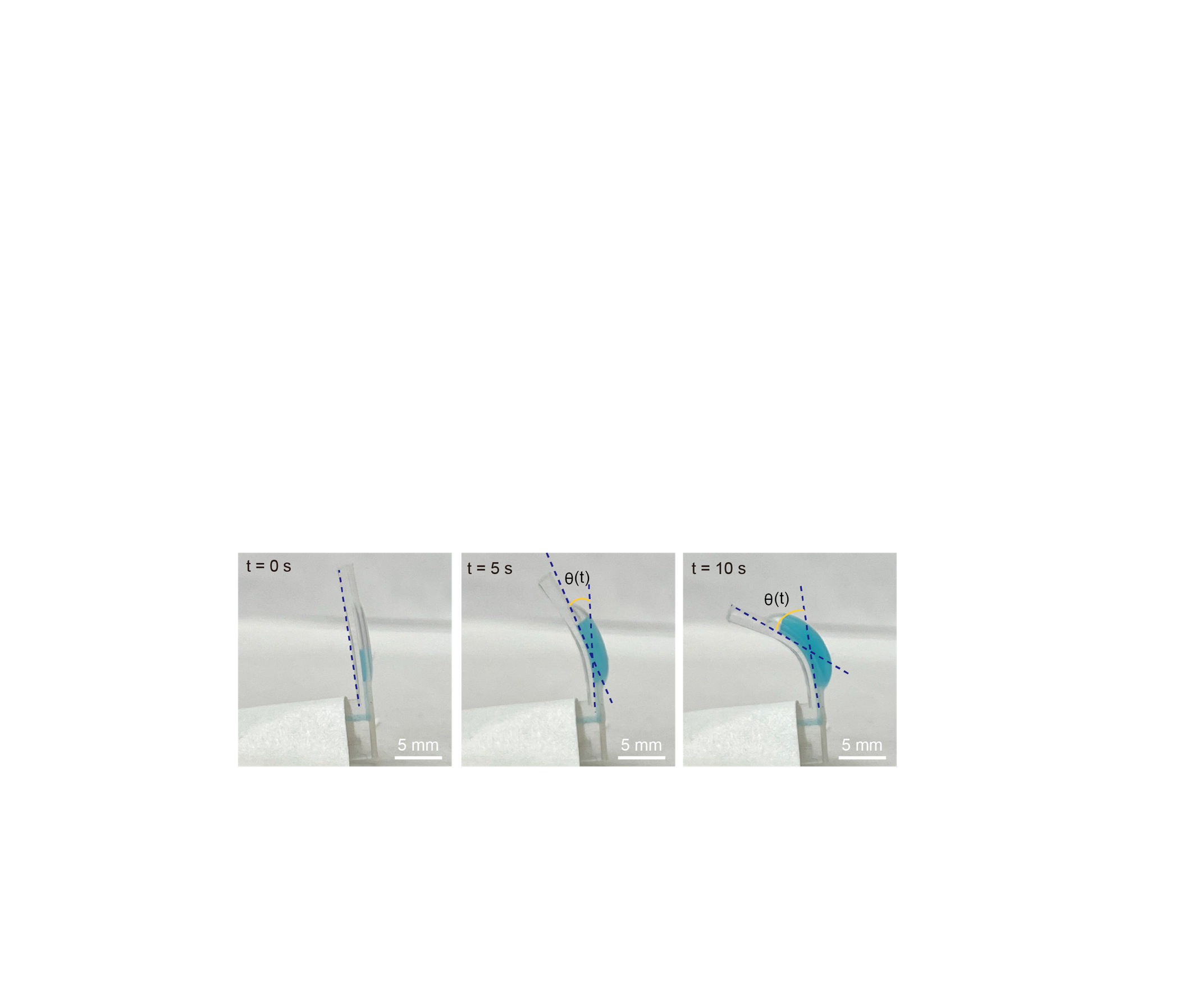
**

**Figure S2.** Bending angle quantification using ImageJ software. θ(t) was defined as the bending angle over time. The standard control balloon actuator images at 0, 5 and 10 s during bending motion are shown here as representative images.

**Figure S3.**

**Figure S2.** Force-displacement graph of rubber band. Spring constant (in N/mm) was calculated by measuring the displacement of rubber when 0.1, 0.2, 0.3, 0.4, 0.5 N load was applied.

**Figure S4.**

**
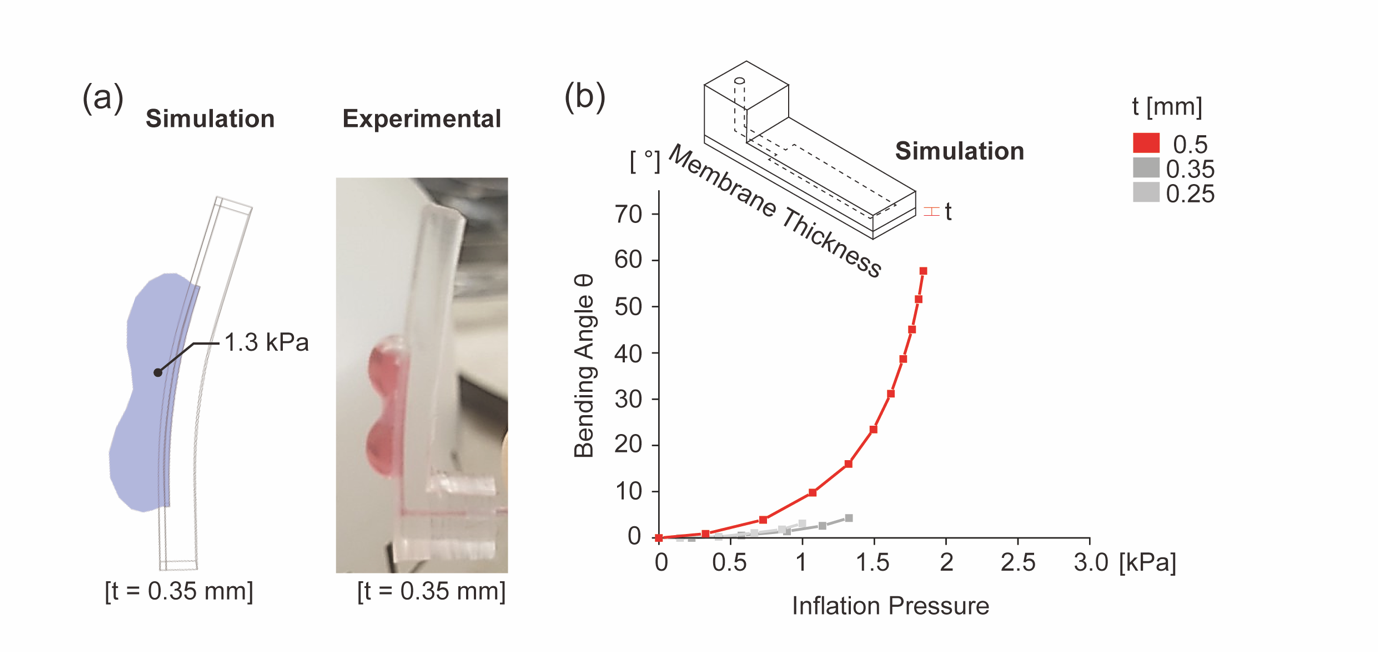
**

**Figure S4.** Simulation and experimental results for lower thicknesses of body B. (a) simulation and experimental results of balloon actuation when t = 0.35mm. Because of small thick- ness of body B, the membrane expansion creates two balloons, resulting bending angle decreases. (b) simulation results of bending angle when t = 0.25 mm, 0.35 mm, 0.5 mm. By the balloon separation, the maximum bending angle is dramatically reduced, and body B eventually tears when a certain pressure is exceeded.

**Supplementary Tables**

**Table S1.** Physical parameters used in the COMSOL simulation

| Component | Symbol | Value | Description |
| --- | --- | --- | --- |
| Fluid domain | $\eta_{f}$ | $8.9 \times{10}^{-4} kg/ms$ | Dynamic viscosity of water |
|  | $\rho_{f}$ | 1000 $kg/m^{3}$ | Water density |
| PDMS | $\rho_{p}$ | 970 $kg/m^{3}$ | Density of PDMS |
|  | $K_{p}$ | $9.62 \times{10}^{8} \mathrm{Pa} ADDIN ZOTERO\_TEMP ADDIN ZOTERO\_TEMP$ | Bulk modulus^1^ |
| Ecoflex membrane | $\rho_{e}$ | 1070 $kg/m^{3}$ | Density of membrane |
|  | $K_{e}$ | $5.5 \times{10}^{4} \mathrm{Pa}$ | Bulk modulus |

**Table S2.** The pressure constant $\tau$ in different reservoir length of balloon actuator were calculated by exponential growth curve fitting. The A value from Eq (5) was fixed at 0.65 to perform curve fitting.

|  | L: 4.1 mm | L: 7.1 mm | L: 11.1 mm | L: 15.1 mm |
| --- | --- | --- | --- | --- |
| $\tau$ | 475.4 ± 4.967 | 412.2 ± 0.902 | 366.0 ± 2.421 | 330.4 ± 3.725 |
| R^2^ | 0.945 | 0.998 | 0.983 | 0.956 |

**Table S3.** The amplification constant $A$ in different membrane thickness of balloon actuator were calculated by exponential growth curve fitting. The $\tau$ value from Eq (5) was fixed at 420 to perform curve fitting.

|  | 0.5 mm | 0.75 mm | 1 mm |
| --- | --- | --- | --- |
| A | 0.704 ± 0.007 | 0.211 ± 0.006 | 0.069 ± 0.003 |
| R^2^ | 0.998 | 0.980 | 0.943 |

**References:**

1. Kim TK, Kim JK, Jeong OC. Measurement of Nonlinear Mechanical Properties of PDMS Elastomer. Microelectron. Eng. 2011;88(8):1982–1985.

**COMSOL Report**

Contents

[1. Global Definitions](#cs9393678)

[1.1. Parameters](#cs4542033)

[2. Component 1](#cs6307543)

[2.1. Definitions](#cs5668389)

[2.2. Geometry 1](#cs9869335)

[2.3. Materials](#cs7635728)

[2.4. Laminar Flow](#cs5478702)

[2.5. Solid Mechanics](#cs9251917)

[2.6. Multiphysics](#cs6600336)

[2.7. Mesh 1](#cs7572002)

[3. Study 1](#cs9313594)

[3.1. Time Dependent](#cs8894568)

[3.2. Solver Configurations](#cs3109802)

[4. Results](#cs2732099)

[4.1. Data Sets](#cs7730187)

[4.2. Plot Groups](#cs4916199)

1. Global Definitions

Global settings

| Name | Balloon_actuator |
| --- | --- |
| Path | C:\Users\vk64\Desktop\Balloon_actuator.mph |
| Version | COMSOL Multiphysics 5.5 (Build: 359) |
| Unit system | SI |

Used products

| COMSOL Multiphysics |
| --- |
| Nonlinear Structural Materials Module |
| Structural Mechanics Module |

- 1. Parameters

Parameters 1

| **Name** | **Expression** | **Value** | **Description** |
| --- | --- | --- | --- |
| Wpdms | 3. [mm] | 0.003 m | width of PDMS |
| Lb | 7.1 [mm] | 0.0071 m | Length of balloon |

1. Component 1

| Date | Dec 16, 2019 12:21:55 PM |
| --- | --- |

Settings

| **Description** | **Value** |
| --- | --- |
| Unit system | Same as global system |
| Geometry shape order | Automatic |
| Avoid inverted elements by curving interior domain elements | Off |

Spatial frame coordinates

| **First** | **Second** | **Third** |
| --- | --- | --- |
| x | y | z |

Material frame coordinates

| **First** | **Second** | **Third** |
| --- | --- | --- |
| X | Y | Z |

Geometry frame coordinates

| **First** | **Second** | **Third** |
| --- | --- | --- |
| Xg | Yg | Zg |

Mesh frame coordinates

| **First** | **Second** | **Third** |
| --- | --- | --- |
| Xm | Ym | Zm |

- 1. Definitions
     1. Functions

#### Step 1

| Function name | step1 |
| --- | --- |
| Function type | Step |


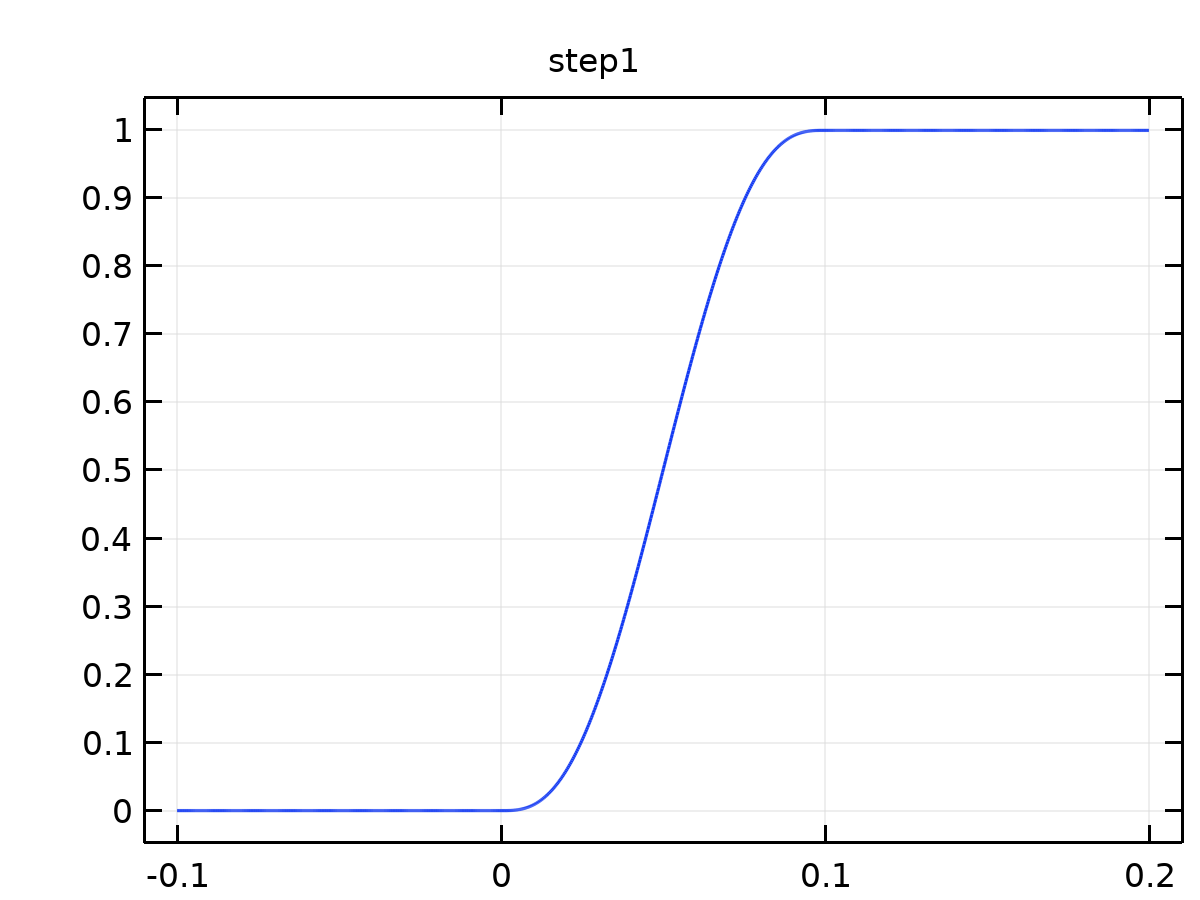


Step 1

Parameters

| **Description** | **Value** |
| --- | --- |
| Location | 0.05 |
| From | 0 |
| To | 1 |

- - 1. Coordinate Systems

#### Boundary System 1

| Coordinate system type | Boundary system |
| --- | --- |
| Tag | sys1 |

Coordinate names

| **First** | **Second** | **Third** |
| --- | --- | --- |
| t1 | t2 | n |

- - 1. Moving Mesh

#### Deforming Domain 1

| Tag | free1 |
| --- | --- |

Selection

| Geometric entity level | Domain |
| --- | --- |
| Selection | Geometry geom1: Dimension 3: Domain 3 |


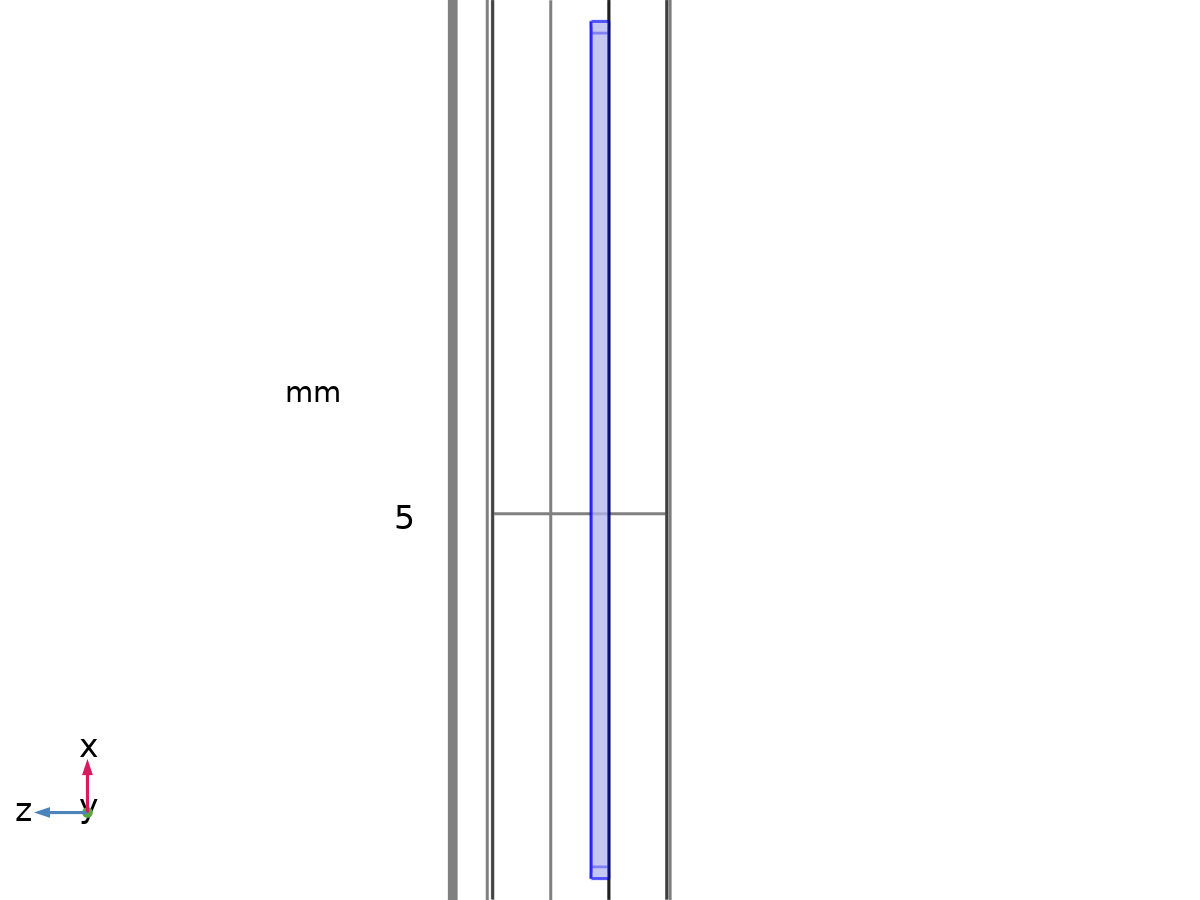


Selection

Smoothing

| **Description** | **Value** |
| --- | --- |
| Mesh smoothing type | Hyperelastic |

Initial deformation

| **Description** | **Value** |
| --- | --- |
| Initial deformation | {0, 0, 0} |

- 1. Geometry 1


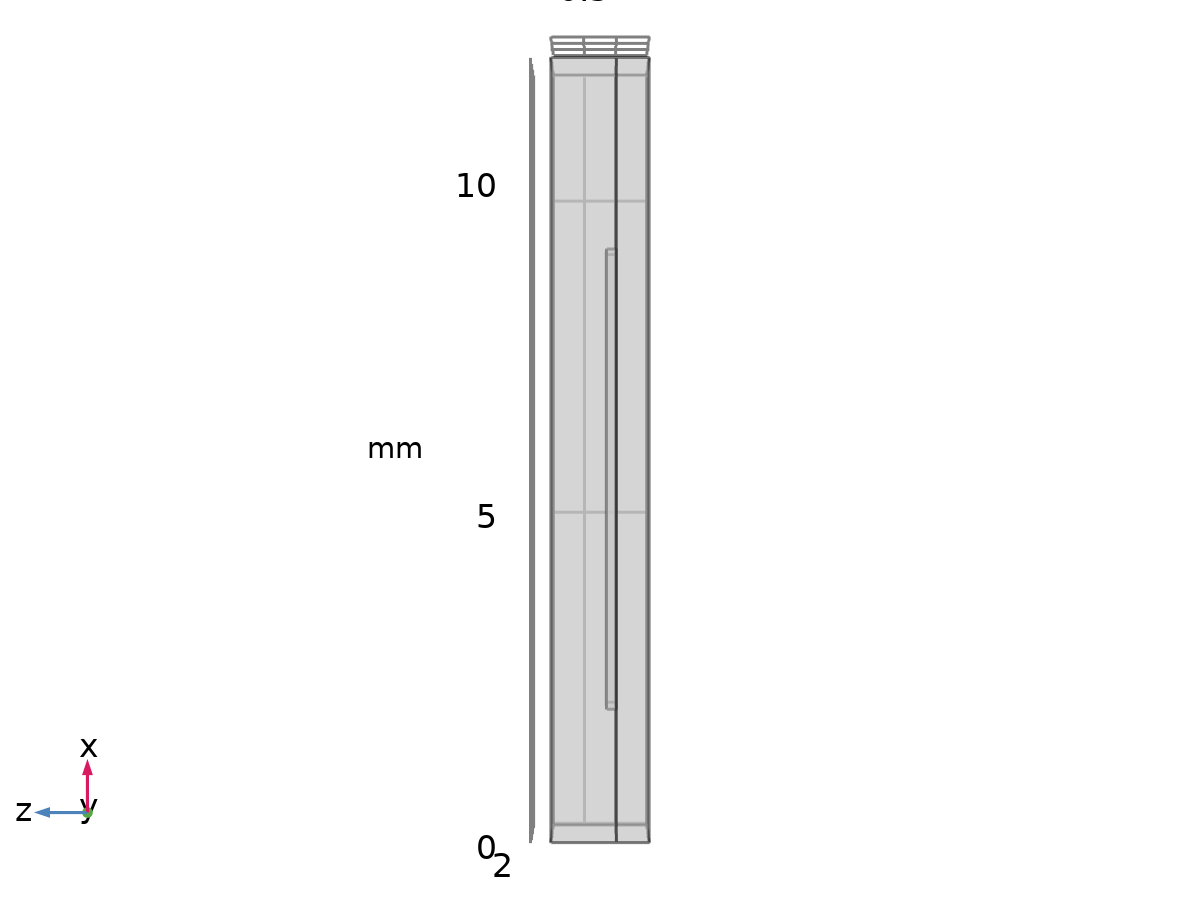


Geometry 1

Units

| Length unit | mm |
| --- | --- |
| Angular unit | deg |

Geometry statistics

| **Description** | **Value** |
| --- | --- |
| Space dimension | 3 |
| Number of domains | 3 |
| Number of boundaries | 17 |
| Number of edges | 32 |
| Number of vertices | 20 |

- - 1. Block 1 (blk1)

Position

| **Description** | **Value** |
| --- | --- |
| Position | {0, 0, 0} |

Axis

| **Description** | **Value** |
| --- | --- |
| Axis type | z - axis |

Size and shape

| **Description** | **Value** |
| --- | --- |
| Width | 12 |
| Depth | Wpdms |
| Height | 1.0 |

- - 1. Block 2 (blk2)

Position

| **Description** | **Value** |
| --- | --- |
| Position | {2.5 - 0.5, (Wpdms - 1.8)/2.0, 0} |

Axis

| **Description** | **Value** |
| --- | --- |
| Axis type | z - axis |

Size and shape

| **Description** | **Value** |
| --- | --- |
| Width | Lb |
| Depth | 1.8 |
| Height | 0.15 |

- - 1. Block 3 (blk3)

Position

| **Description** | **Value** |
| --- | --- |
| Position | {2.5 + Lb, (Wpdms - 0.2)/2.0, 0} |

Axis

| **Description** | **Value** |
| --- | --- |
| Axis type | z - axis |

Size and shape

| **Description** | **Value** |
| --- | --- |
| Width | 2.4 |
| Depth | 0.2 |
| Height | 0.15 |

- - 1. Block 4 (blk4)

Position

| **Description** | **Value** |
| --- | --- |
| Position | {9, 0, 1} |

Axis

| **Description** | **Value** |
| --- | --- |
| Axis type | z - axis |

Size and shape

| **Description** | **Value** |
| --- | --- |
| Width | 3 |
| Depth | Wpdms |
| Height | 0.8 |

- - 1. Cylinder 1 (cyl1)

Position

| **Description** | **Value** |
| --- | --- |
| Position | {10.5, Wpdms/2, 0} |

Axis

| **Description** | **Value** |
| --- | --- |
| Axis type | z - axis |

Size and shape

| **Description** | **Value** |
| --- | --- |
| Radius | 0.15 |
| Height | 1.0 |

- - 1. Union 1 (uni1)

Compose

| **Description** | **Value** |
| --- | --- |
| Keep interior boundaries | Off |

- - 1. Union 2 (uni2)

Compose

| **Description** | **Value** |
| --- | --- |
| Keep interior boundaries | Off |

- - 1. Difference 1 (dif1)

Settings

| **Description** | **Value** |
| --- | --- |
| Keep input objects | On |

Compose

| **Description** | **Value** |
| --- | --- |
| Keep interior boundaries | Off |

- - 1. Block 5 (blk5)

Position

| **Description** | **Value** |
| --- | --- |
| Position | {0, 0, -0.5} |

Axis

| **Description** | **Value** |
| --- | --- |
| Axis type | z - axis |

Size and shape

| **Description** | **Value** |
| --- | --- |
| Width | 12 |
| Depth | Wpdms |
| Height | 0.5 |

- 1. Materials
     1. Water


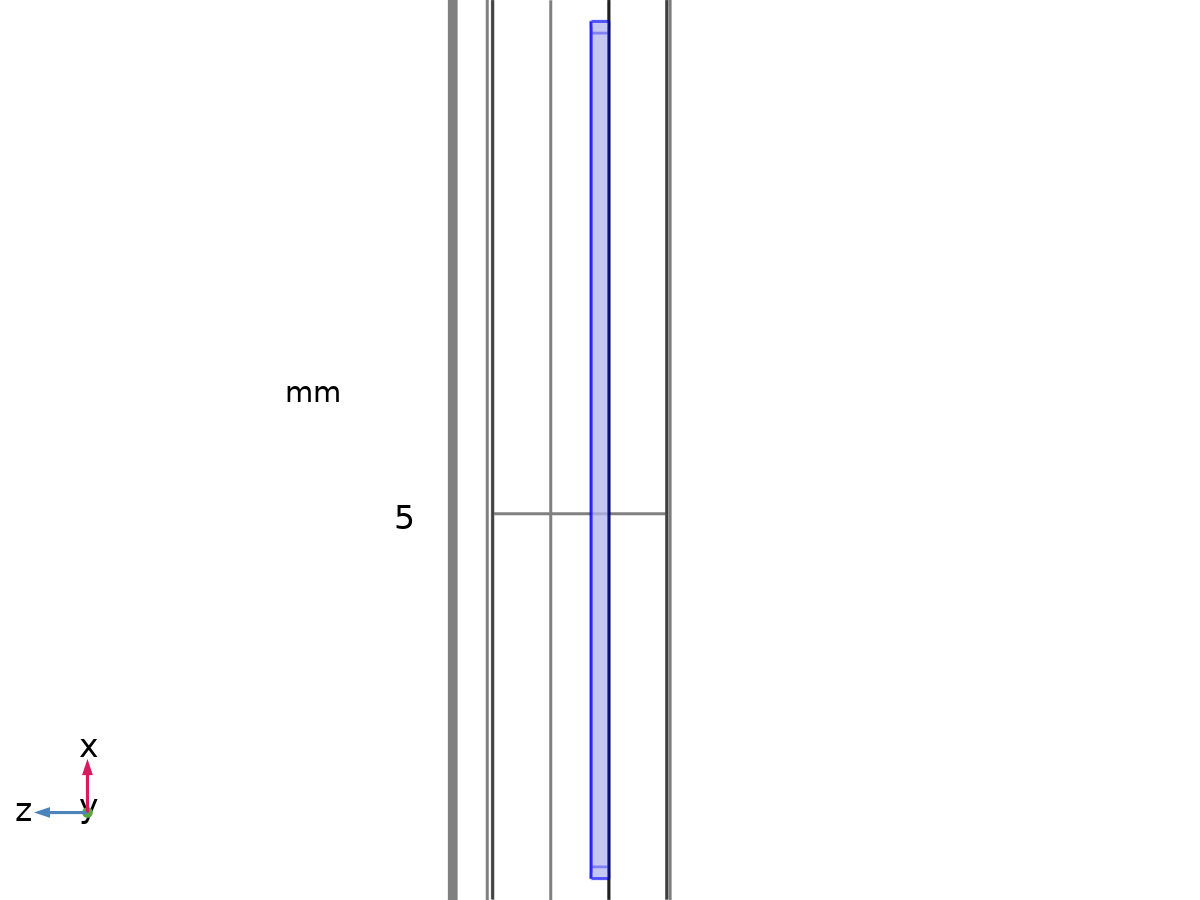


Water

Selection

| Geometric entity level | Domain |
| --- | --- |
| Selection | Geometry geom1: Dimension 3: Domain 3 |

Material parameters

| **Name** | **Value** | **Unit** |
| --- | --- | --- |
| Dynamic viscosity | eta(T[1/K])[Pa*s] | Pa·s |
| Density | rho(T[1/K])[kg/m^3] | kg/m³ |

Basic Settings

| **Description** | **Value** |
| --- | --- |
| Dynamic viscosity | eta(T[1/K])[Pa*s] |
| Ratio of specific heats | 1.0 |
| Electrical conductivity | {{5.5e-6[S/m], 0, 0}, {0, 5.5e-6[S/m], 0}, {0, 0, 5.5e-6[S/m]}} |
| Heat capacity at constant pressure | Cp(T[1/K])[J/(kg*K)] |
| Density | rho(T[1/K])[kg/m^3] |
| Thermal conductivity | {{k(T[1/K])[W/(m*K)], 0, 0}, {0, k(T[1/K])[W/(m*K)], 0}, {0, 0, k(T[1/K])[W/(m*K)]}} |
| Speed of sound | cs(T[1/K])[m/s] |
| Poisson's ratio | 0 |

Functions

| **Function name** | **Type** |
| --- | --- |
| eta | Piecewise |
| Cp | Piecewise |
| rho | Piecewise |
| k | Piecewise |
| cs | Interpolation |


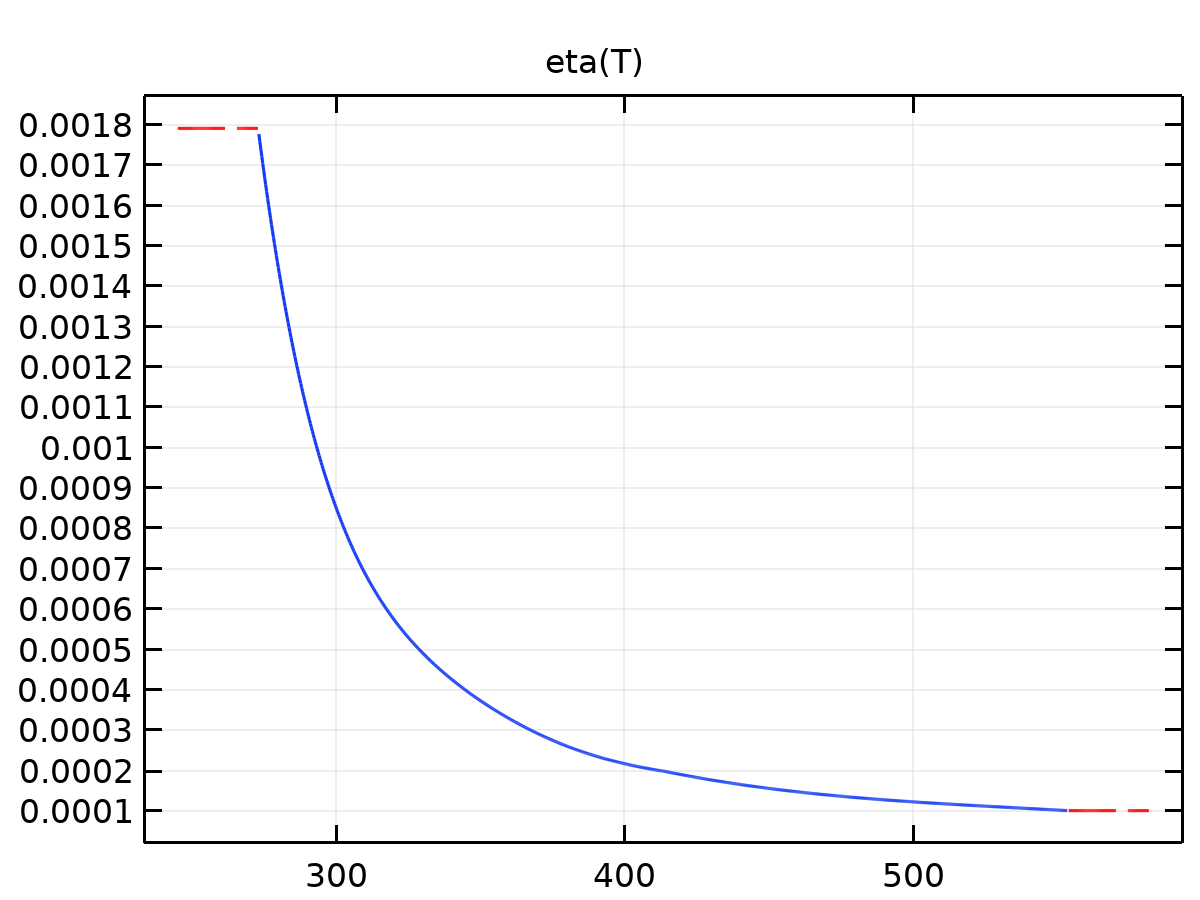


eta


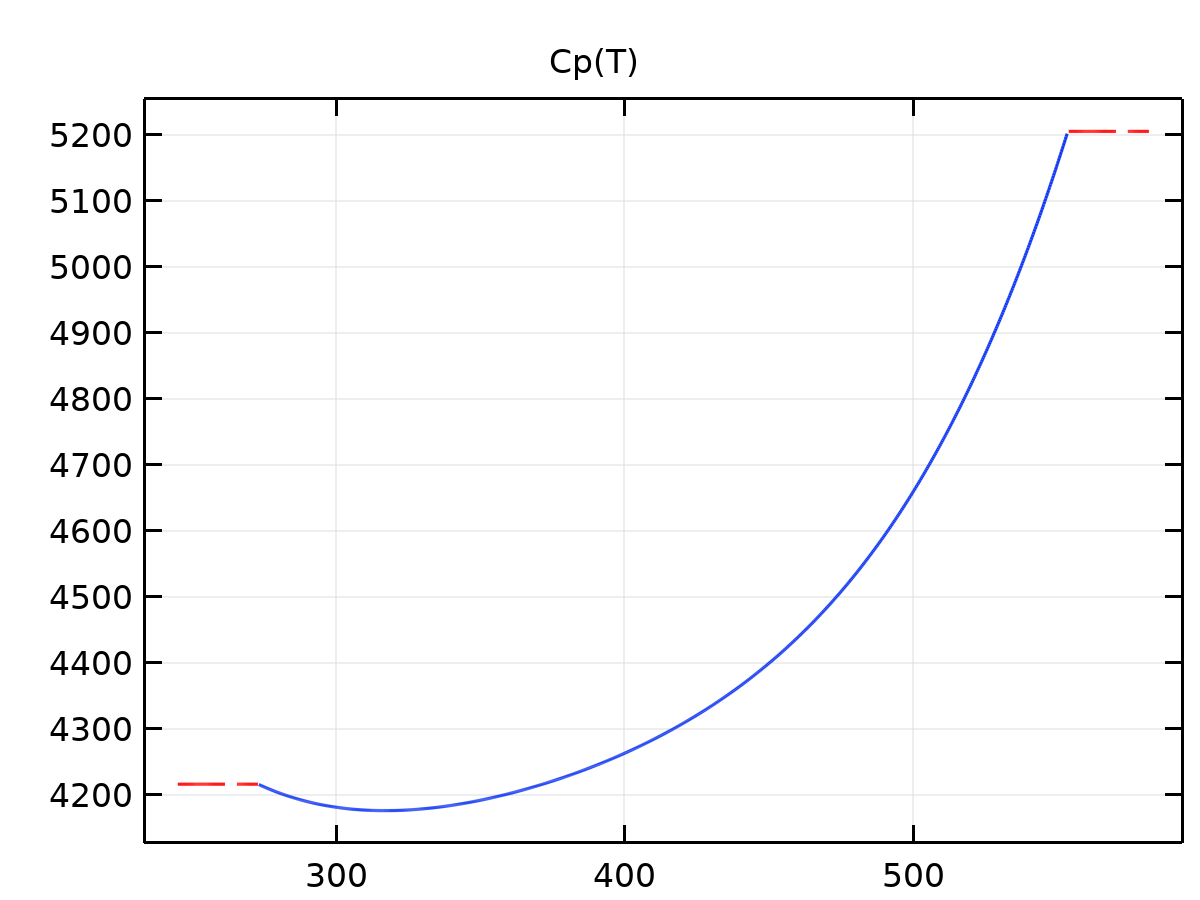


Cp


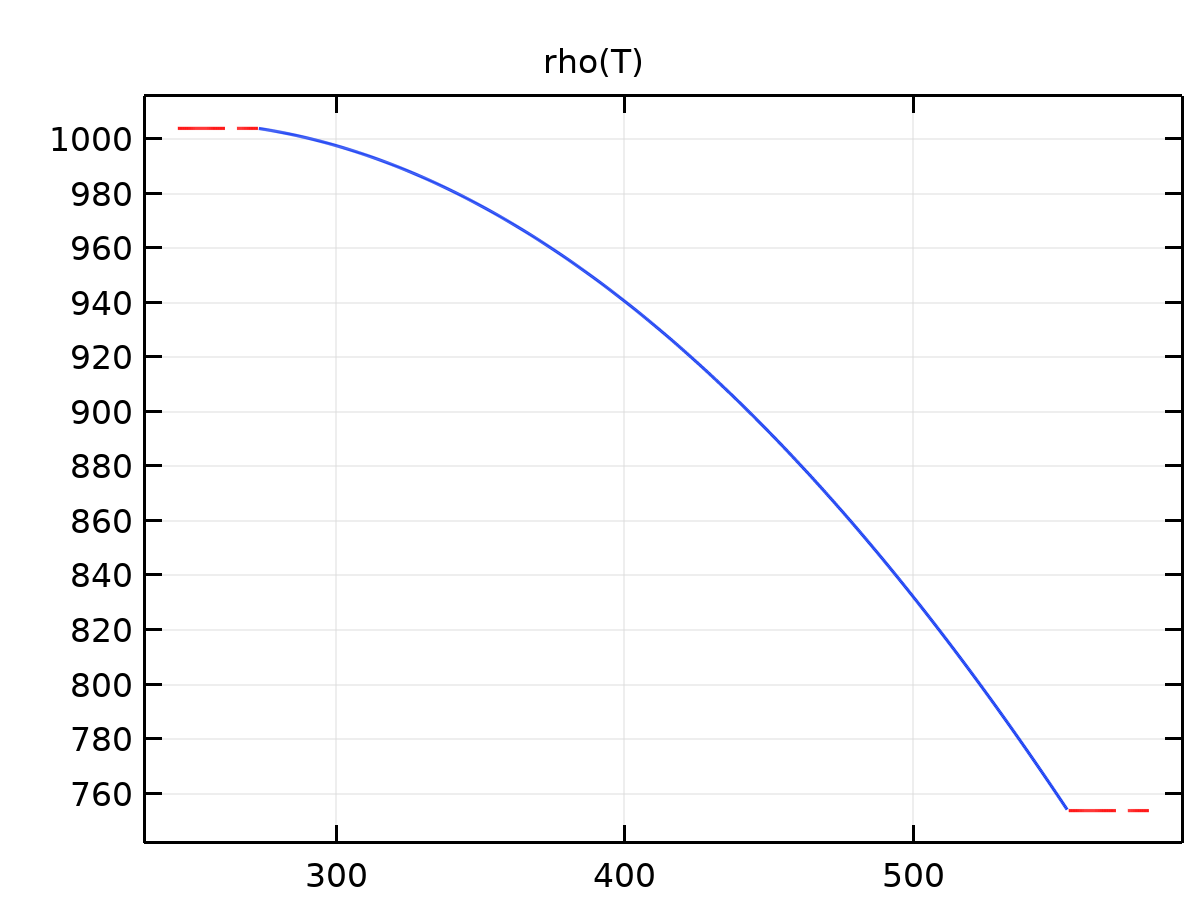


rho


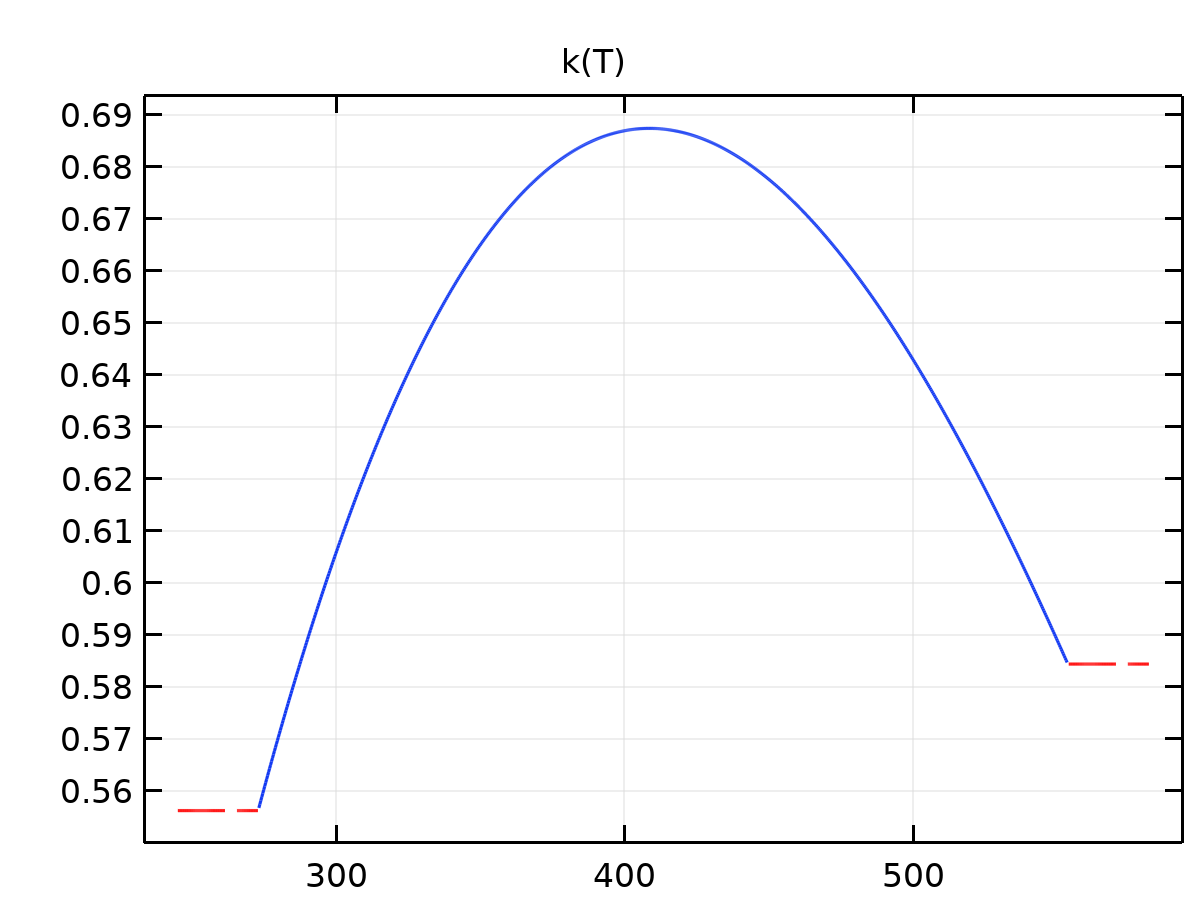


k


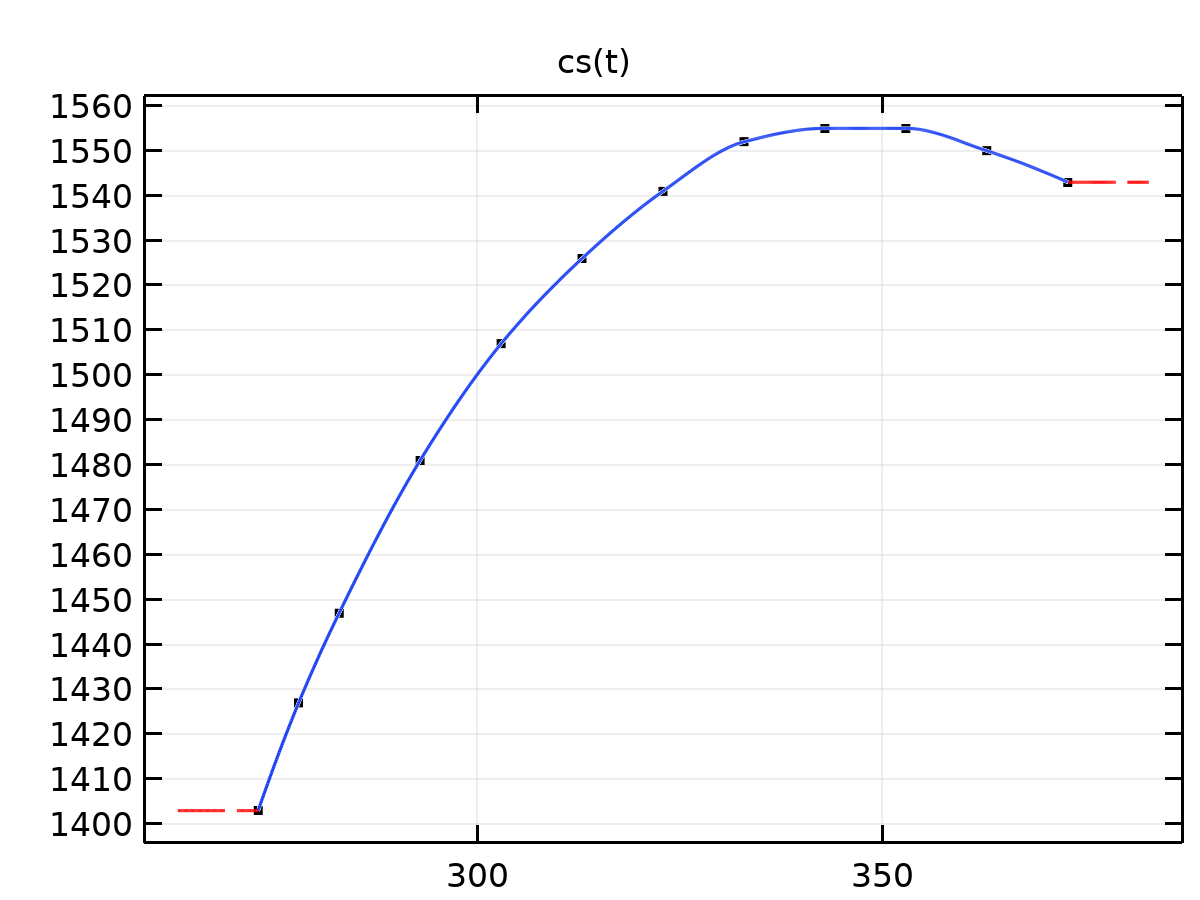


cs

- - 1. PDMS - Polydimethylsiloxane


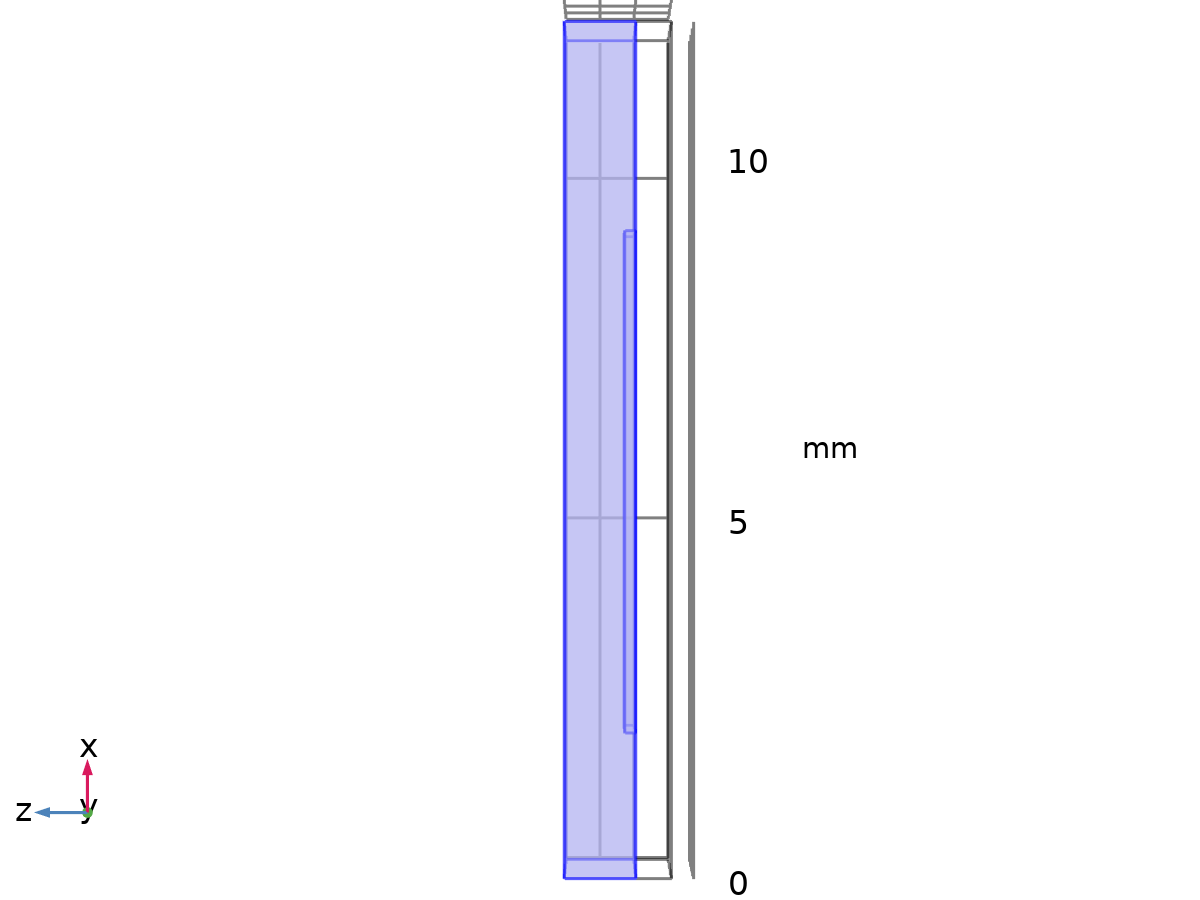


PDMS - Polydimethylsiloxane

Selection

| Geometric entity level | Domain |
| --- | --- |
| Selection | Geometry geom1: Dimension 3: Domain 2 |

Material parameters

| **Name** | **Value** | **Unit** |
| --- | --- | --- |
| Density | 970[kg/m^3] | kg/m³ |

Basic Settings

| **Description** | **Value** |
| --- | --- |
| Coefficient of thermal expansion | {{9e-4[1/K], 0, 0}, {0, 9e-4[1/K], 0}, {0, 0, 9e-4[1/K]}} |
| Heat capacity at constant pressure | 1460[J/(kg*K)] |
| Relative permittivity | {{2.75, 0, 0}, {0, 2.75, 0}, {0, 0, 2.75}} |
| Density | 970[kg/m^3] |
| Thermal conductivity | {{0.16[W/(m*K)], 0, 0}, {0, 0.16[W/(m*K)], 0}, {0, 0, 0.16[W/(m*K)]}} |

Young's modulus and Poisson's ratio Settings

| **Description** | **Value** |
| --- | --- |
| Young's modulus | 750[kPa] |
| Poisson's ratio | 0.49 |

- 1. Laminar Flow

Used products

| COMSOL Multiphysics |
| --- |


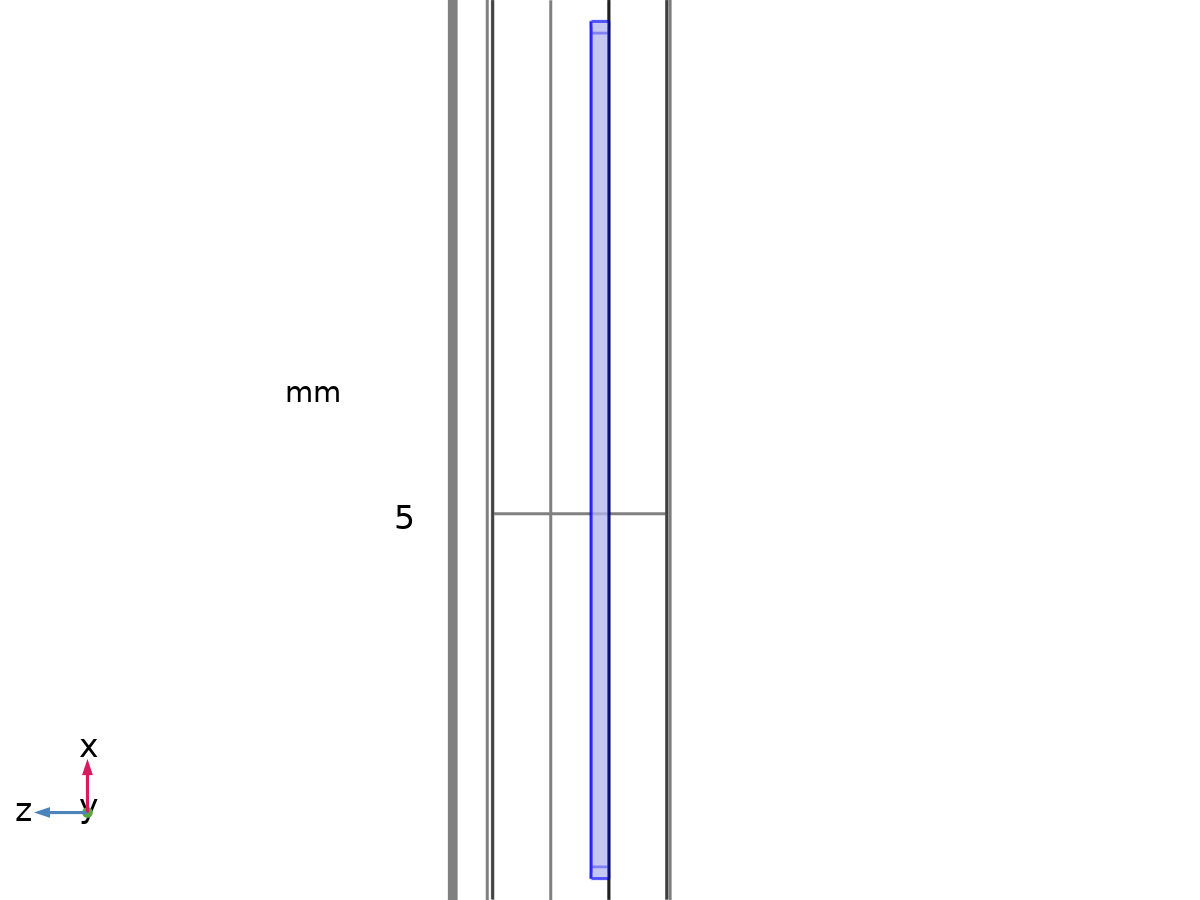


Laminar Flow

Selection

| Geometric entity level | Domain |
| --- | --- |
| Selection | Geometry geom1: Dimension 3: Domain 3 |

Equations


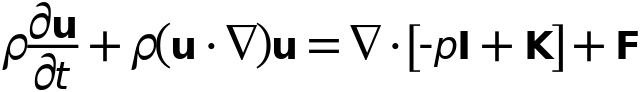


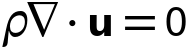


- - 1. Interface settings

#### Discretization

Settings

| **Description** | **Value** |
| --- | --- |
| Discretization of fluids | P1 + P1 |

#### Physical model

Settings

| **Description** | **Value** |
| --- | --- |
| Neglect inertial term (Stokes flow) | Off |
| Compressibility | Incompressible flow |
| Enable porous media domains | Off |
| Include gravity | Off |
| Reference temperature | 293.15[K] |
| Reference pressure level | 1[atm] |
| Reference temperature | User defined |

#### Turbulence

Settings

| **Description** | **Value** |
| --- | --- |
| Turbulence model type | None |

- - 1. Variables

| **Name** | **Expression** | **Unit** | **Description** | **Selection** | **Details** |
| --- | --- | --- | --- | --- | --- |
| spf.usePseudoTimeStepping | 0 | 1 | Help variable | Global | + operation |
| spf.Tref | model.input.Tref | K | Reference temperature | Global | Meta |
| spf.dz | 1 | m | Thickness | Domain 3 |  |
| spf.pref | 1[atm] | Pa | Reference pressure level | Domain 3 |  |
| spf.pA | p+spf.pref | Pa | Absolute pressure | Domain 3 |  |
| spf.hasWF | 0 |  | Help variable | Boundaries 10–15 |  |
| spf.dt_CFL | 1/max(spf.maxOp(sqrt(emetric_spatial(u-d(x,TIME),v-d(y,TIME),w-d(z,TIME)))),eps) | s | Time step, CFL=1 | Global |  |
| spf.CFL_number | timestep/spf.dt_CFL |  | CFL number | Global |  |
| spf.nx | dnx | 1 | Normal vector, x component | Boundaries 10–15 |  |
| spf.ny | dny | 1 | Normal vector, y component | Boundaries 10–15 |  |
| spf.nz | dnz | 1 | Normal vector, z component | Boundaries 10–15 |  |
| spf.nxmesh | dnxmesh | 1 | Normal vector, x component | Boundaries 10–15 |  |
| spf.nymesh | dnymesh | 1 | Normal vector, y component | Boundaries 10–15 |  |
| spf.nzmesh | dnzmesh | 1 | Normal vector, z component | Boundaries 10–15 |  |

- - 1. Fluid Properties 1


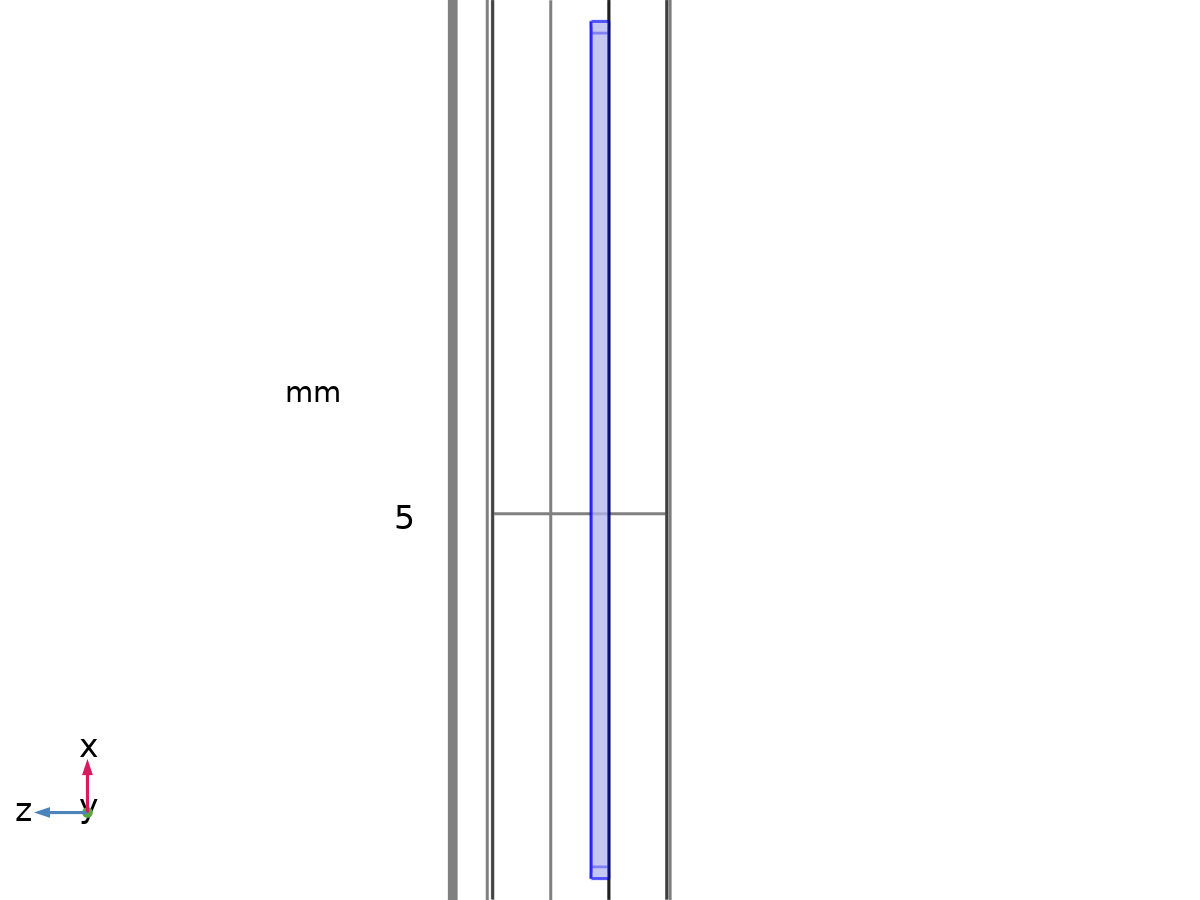


Fluid Properties 1

Selection

| Geometric entity level | Domain |
| --- | --- |
| Selection | Geometry geom1: Dimension 3: All domains |

Equations


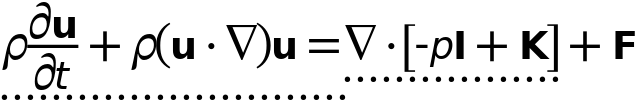


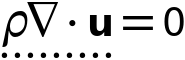


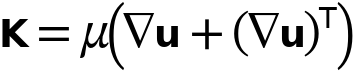


#### Fluid properties

Settings

| **Description** | **Value** |
| --- | --- |
| Density | From material |
|  | Newtonian |
| Dynamic viscosity | From material |

#### Model input

Settings

| **Description** | **Value** |
| --- | --- |
| Temperature | User defined |
| Temperature | 293.15[K] |

Properties from material

| **Property** | **Material** | **Property group** |
| --- | --- | --- |
| Density | Water | Basic |
| Dynamic viscosity | Water | Basic |

#### Variables

| **Name** | **Expression** | **Unit** | **Description** | **Selection** | **Details** |
| --- | --- | --- | --- | --- | --- |
| spf.mu | material.mu | Pa·s | Dynamic viscosity | Domain 3 | Meta |
| spf.rho | subst(material.rho,spf.fp1.minput_temperature,spf.Trho,spf.fp1.minput_pressure,spf.prho) | kg/m³ | Density | Domain 3 | Meta |
| uxt | uxTIME-uxx*d(x,TIME)-uxy*d(y,TIME)-uxz*d(z,TIME) | 1/s² | Gradient of u, x component, first time derivative | Domain 3 |  |
| uyt | uyTIME-uyx*d(x,TIME)-uyy*d(y,TIME)-uyz*d(z,TIME) | 1/s² | Gradient of u, y component, first time derivative | Domain 3 |  |
| uzt | uzTIME-uzx*d(x,TIME)-uzy*d(y,TIME)-uzz*d(z,TIME) | 1/s² | Gradient of u, z component, first time derivative | Domain 3 |  |
| uxtt | d(uxTIME-uxx*d(x,TIME)-uxy*d(y,TIME)-uxz*d(z,TIME),TIME)-d(uxTIME-uxx*d(x,TIME)-uxy*d(y,TIME)-uxz*d(z,TIME),x)*d(x,TIME)-d(uxTIME-uxx*d(x,TIME)-uxy*d(y,TIME)-uxz*d(z,TIME),y)*d(y,TIME)-d(uxTIME-uxx*d(x,TIME)-uxy*d(y,TIME)-uxz*d(z,TIME),z)*d(z,TIME) | 1/s³ | Gradient of u, x component, second time derivative | Domain 3 |  |
| uytt | d(uyTIME-uyx*d(x,TIME)-uyy*d(y,TIME)-uyz*d(z,TIME),TIME)-d(uyTIME-uyx*d(x,TIME)-uyy*d(y,TIME)-uyz*d(z,TIME),x)*d(x,TIME)-d(uyTIME-uyx*d(x,TIME)-uyy*d(y,TIME)-uyz*d(z,TIME),y)*d(y,TIME)-d(uyTIME-uyx*d(x,TIME)-uyy*d(y,TIME)-uyz*d(z,TIME),z)*d(z,TIME) | 1/s³ | Gradient of u, y component, second time derivative | Domain 3 |  |
| uztt | d(uzTIME-uzx*d(x,TIME)-uzy*d(y,TIME)-uzz*d(z,TIME),TIME)-d(uzTIME-uzx*d(x,TIME)-uzy*d(y,TIME)-uzz*d(z,TIME),x)*d(x,TIME)-d(uzTIME-uzx*d(x,TIME)-uzy*d(y,TIME)-uzz*d(z,TIME),y)*d(y,TIME)-d(uzTIME-uzx*d(x,TIME)-uzy*d(y,TIME)-uzz*d(z,TIME),z)*d(z,TIME) | 1/s³ | Gradient of u, z component, second time derivative | Domain 3 |  |
| vxt | vxTIME-vxx*d(x,TIME)-vxy*d(y,TIME)-vxz*d(z,TIME) | 1/s² | Gradient of v, x component, first time derivative | Domain 3 |  |
| vyt | vyTIME-vyx*d(x,TIME)-vyy*d(y,TIME)-vyz*d(z,TIME) | 1/s² | Gradient of v, y component, first time derivative | Domain 3 |  |
| vzt | vzTIME-vzx*d(x,TIME)-vzy*d(y,TIME)-vzz*d(z,TIME) | 1/s² | Gradient of v, z component, first time derivative | Domain 3 |  |
| vxtt | d(vxTIME-vxx*d(x,TIME)-vxy*d(y,TIME)-vxz*d(z,TIME),TIME)-d(vxTIME-vxx*d(x,TIME)-vxy*d(y,TIME)-vxz*d(z,TIME),x)*d(x,TIME)-d(vxTIME-vxx*d(x,TIME)-vxy*d(y,TIME)-vxz*d(z,TIME),y)*d(y,TIME)-d(vxTIME-vxx*d(x,TIME)-vxy*d(y,TIME)-vxz*d(z,TIME),z)*d(z,TIME) | 1/s³ | Gradient of v, x component, second time derivative | Domain 3 |  |
| vytt | d(vyTIME-vyx*d(x,TIME)-vyy*d(y,TIME)-vyz*d(z,TIME),TIME)-d(vyTIME-vyx*d(x,TIME)-vyy*d(y,TIME)-vyz*d(z,TIME),x)*d(x,TIME)-d(vyTIME-vyx*d(x,TIME)-vyy*d(y,TIME)-vyz*d(z,TIME),y)*d(y,TIME)-d(vyTIME-vyx*d(x,TIME)-vyy*d(y,TIME)-vyz*d(z,TIME),z)*d(z,TIME) | 1/s³ | Gradient of v, y component, second time derivative | Domain 3 |  |
| vztt | d(vzTIME-vzx*d(x,TIME)-vzy*d(y,TIME)-vzz*d(z,TIME),TIME)-d(vzTIME-vzx*d(x,TIME)-vzy*d(y,TIME)-vzz*d(z,TIME),x)*d(x,TIME)-d(vzTIME-vzx*d(x,TIME)-vzy*d(y,TIME)-vzz*d(z,TIME),y)*d(y,TIME)-d(vzTIME-vzx*d(x,TIME)-vzy*d(y,TIME)-vzz*d(z,TIME),z)*d(z,TIME) | 1/s³ | Gradient of v, z component, second time derivative | Domain 3 |  |
| wxt | wxTIME-wxx*d(x,TIME)-wxy*d(y,TIME)-wxz*d(z,TIME) | 1/s² | Gradient of w, x component, first time derivative | Domain 3 |  |
| wyt | wyTIME-wyx*d(x,TIME)-wyy*d(y,TIME)-wyz*d(z,TIME) | 1/s² | Gradient of w, y component, first time derivative | Domain 3 |  |
| wzt | wzTIME-wzx*d(x,TIME)-wzy*d(y,TIME)-wzz*d(z,TIME) | 1/s² | Gradient of w, z component, first time derivative | Domain 3 |  |
| wxtt | d(wxTIME-wxx*d(x,TIME)-wxy*d(y,TIME)-wxz*d(z,TIME),TIME)-d(wxTIME-wxx*d(x,TIME)-wxy*d(y,TIME)-wxz*d(z,TIME),x)*d(x,TIME)-d(wxTIME-wxx*d(x,TIME)-wxy*d(y,TIME)-wxz*d(z,TIME),y)*d(y,TIME)-d(wxTIME-wxx*d(x,TIME)-wxy*d(y,TIME)-wxz*d(z,TIME),z)*d(z,TIME) | 1/s³ | Gradient of w, x component, second time derivative | Domain 3 |  |
| wytt | d(wyTIME-wyx*d(x,TIME)-wyy*d(y,TIME)-wyz*d(z,TIME),TIME)-d(wyTIME-wyx*d(x,TIME)-wyy*d(y,TIME)-wyz*d(z,TIME),x)*d(x,TIME)-d(wyTIME-wyx*d(x,TIME)-wyy*d(y,TIME)-wyz*d(z,TIME),y)*d(y,TIME)-d(wyTIME-wyx*d(x,TIME)-wyy*d(y,TIME)-wyz*d(z,TIME),z)*d(z,TIME) | 1/s³ | Gradient of w, y component, second time derivative | Domain 3 |  |
| wztt | d(wzTIME-wzx*d(x,TIME)-wzy*d(y,TIME)-wzz*d(z,TIME),TIME)-d(wzTIME-wzx*d(x,TIME)-wzy*d(y,TIME)-wzz*d(z,TIME),x)*d(x,TIME)-d(wzTIME-wzx*d(x,TIME)-wzy*d(y,TIME)-wzz*d(z,TIME),y)*d(y,TIME)-d(wzTIME-wzx*d(x,TIME)-wzy*d(y,TIME)-wzz*d(z,TIME),z)*d(z,TIME) | 1/s³ | Gradient of w, z component, second time derivative | Domain 3 |  |
| ut | uTIME-ux*d(x,TIME)-uy*d(y,TIME)-uz*d(z,TIME) | m/s² | Velocity field, first time derivative, x component | Domain 3 |  |
| vt | vTIME-vx*d(x,TIME)-vy*d(y,TIME)-vz*d(z,TIME) | m/s² | Velocity field, first time derivative, y component | Domain 3 |  |
| wt | wTIME-wx*d(x,TIME)-wy*d(y,TIME)-wz*d(z,TIME) | m/s² | Velocity field, first time derivative, z component | Domain 3 |  |
| utt | d(uTIME-ux*d(x,TIME)-uy*d(y,TIME)-uz*d(z,TIME),TIME)-d(uTIME-ux*d(x,TIME)-uy*d(y,TIME)-uz*d(z,TIME),x)*d(x,TIME)-d(uTIME-ux*d(x,TIME)-uy*d(y,TIME)-uz*d(z,TIME),y)*d(y,TIME)-d(uTIME-ux*d(x,TIME)-uy*d(y,TIME)-uz*d(z,TIME),z)*d(z,TIME) | m/s³ | Velocity field, second time derivative, x component | Domain 3 |  |
| vtt | d(vTIME-vx*d(x,TIME)-vy*d(y,TIME)-vz*d(z,TIME),TIME)-d(vTIME-vx*d(x,TIME)-vy*d(y,TIME)-vz*d(z,TIME),x)*d(x,TIME)-d(vTIME-vx*d(x,TIME)-vy*d(y,TIME)-vz*d(z,TIME),y)*d(y,TIME)-d(vTIME-vx*d(x,TIME)-vy*d(y,TIME)-vz*d(z,TIME),z)*d(z,TIME) | m/s³ | Velocity field, second time derivative, y component | Domain 3 |  |
| wtt | d(wTIME-wx*d(x,TIME)-wy*d(y,TIME)-wz*d(z,TIME),TIME)-d(wTIME-wx*d(x,TIME)-wy*d(y,TIME)-wz*d(z,TIME),x)*d(x,TIME)-d(wTIME-wx*d(x,TIME)-wy*d(y,TIME)-wz*d(z,TIME),y)*d(y,TIME)-d(wTIME-wx*d(x,TIME)-wy*d(y,TIME)-wz*d(z,TIME),z)*d(z,TIME) | m/s³ | Velocity field, second time derivative, z component | Domain 3 |  |
| pxt | pxTIME-pxx*d(x,TIME)-pxy*d(y,TIME)-pxz*d(z,TIME) | kg/(m²·s³) | Gradient of p, x component, first time derivative | Domain 3 |  |
| pyt | pyTIME-pyx*d(x,TIME)-pyy*d(y,TIME)-pyz*d(z,TIME) | kg/(m²·s³) | Gradient of p, y component, first time derivative | Domain 3 |  |
| pzt | pzTIME-pzx*d(x,TIME)-pzy*d(y,TIME)-pzz*d(z,TIME) | kg/(m²·s³) | Gradient of p, z component, first time derivative | Domain 3 |  |
| pxtt | d(pxTIME-pxx*d(x,TIME)-pxy*d(y,TIME)-pxz*d(z,TIME),TIME)-d(pxTIME-pxx*d(x,TIME)-pxy*d(y,TIME)-pxz*d(z,TIME),x)*d(x,TIME)-d(pxTIME-pxx*d(x,TIME)-pxy*d(y,TIME)-pxz*d(z,TIME),y)*d(y,TIME)-d(pxTIME-pxx*d(x,TIME)-pxy*d(y,TIME)-pxz*d(z,TIME),z)*d(z,TIME) | kg/(m²·s⁴) | Gradient of p, x component, second time derivative | Domain 3 |  |
| pytt | d(pyTIME-pyx*d(x,TIME)-pyy*d(y,TIME)-pyz*d(z,TIME),TIME)-d(pyTIME-pyx*d(x,TIME)-pyy*d(y,TIME)-pyz*d(z,TIME),x)*d(x,TIME)-d(pyTIME-pyx*d(x,TIME)-pyy*d(y,TIME)-pyz*d(z,TIME),y)*d(y,TIME)-d(pyTIME-pyx*d(x,TIME)-pyy*d(y,TIME)-pyz*d(z,TIME),z)*d(z,TIME) | kg/(m²·s⁴) | Gradient of p, y component, second time derivative | Domain 3 |  |
| pztt | d(pzTIME-pzx*d(x,TIME)-pzy*d(y,TIME)-pzz*d(z,TIME),TIME)-d(pzTIME-pzx*d(x,TIME)-pzy*d(y,TIME)-pzz*d(z,TIME),x)*d(x,TIME)-d(pzTIME-pzx*d(x,TIME)-pzy*d(y,TIME)-pzz*d(z,TIME),y)*d(y,TIME)-d(pzTIME-pzx*d(x,TIME)-pzy*d(y,TIME)-pzz*d(z,TIME),z)*d(z,TIME) | kg/(m²·s⁴) | Gradient of p, z component, second time derivative | Domain 3 |  |
| pt | pTIME-px*d(x,TIME)-py*d(y,TIME)-pz*d(z,TIME) | Pa/s | Pressure, first time derivative | Domain 3 |  |
| ptt | d(pTIME-px*d(x,TIME)-py*d(y,TIME)-pz*d(z,TIME),TIME)-d(pTIME-px*d(x,TIME)-py*d(y,TIME)-pz*d(z,TIME),x)*d(x,TIME)-d(pTIME-px*d(x,TIME)-py*d(y,TIME)-pz*d(z,TIME),y)*d(y,TIME)-d(pTIME-px*d(x,TIME)-py*d(y,TIME)-pz*d(z,TIME),z)*d(z,TIME) | Pa/s² | Pressure, second time derivative | Domain 3 |  |
| spf.Trho | spf.Tref | K | Temperature for density evaluation | Domain 3 |  |
| spf.prho | spf.pref | Pa | Pressure for the evaluation of density | Domain 3 |  |
| spf.rhoref | subst(material.rho,spf.fp1.minput_temperature,spf.Tref,spf.fp1.minput_pressure,spf.pref) | kg/m³ | Reference density | Domain 3 | Meta |
| spf.mumat | material.mu | Pa·s | Dynamic viscosity | Domain 3 | Meta |
| spf.srijxx | ux | 1/s | Strain rate tensor, xx component | Domain 3 |  |
| spf.srijyx | 0.5*(vx+uy) | 1/s | Strain rate tensor, yx component | Domain 3 |  |
| spf.srijzx | 0.5*(wx+uz) | 1/s | Strain rate tensor, zx component | Domain 3 |  |
| spf.srijxy | 0.5*(uy+vx) | 1/s | Strain rate tensor, xy component | Domain 3 |  |
| spf.srijyy | vy | 1/s | Strain rate tensor, yy component | Domain 3 |  |
| spf.srijzy | 0.5*(wy+vz) | 1/s | Strain rate tensor, zy component | Domain 3 |  |
| spf.srijxz | 0.5*(uz+wx) | 1/s | Strain rate tensor, xz component | Domain 3 |  |
| spf.srijyz | 0.5*(vz+wy) | 1/s | Strain rate tensor, yz component | Domain 3 |  |
| spf.srijzz | wz | 1/s | Strain rate tensor, zz component | Domain 3 |  |
| spf.rrijxx | 0 | 1/s | Rotation rate tensor, xx component | Domain 3 |  |
| spf.rrijyx | 0.5*(vx-uy) | 1/s | Rotation rate tensor, yx component | Domain 3 |  |
| spf.rrijzx | 0.5*(wx-uz) | 1/s | Rotation rate tensor, zx component | Domain 3 |  |
| spf.rrijxy | 0.5*(uy-vx) | 1/s | Rotation rate tensor, xy component | Domain 3 |  |
| spf.rrijyy | 0 | 1/s | Rotation rate tensor, yy component | Domain 3 |  |
| spf.rrijzy | 0.5*(wy-vz) | 1/s | Rotation rate tensor, zy component | Domain 3 |  |
| spf.rrijxz | 0.5*(uz-wx) | 1/s | Rotation rate tensor, xz component | Domain 3 |  |
| spf.rrijyz | 0.5*(vz-wy) | 1/s | Rotation rate tensor, yz component | Domain 3 |  |
| spf.rrijzz | 0 | 1/s | Rotation rate tensor, zz component | Domain 3 |  |
| spf.sr | sqrt(2*spf.srijxx^2+2*spf.srijxy^2+2*spf.srijxz^2+2*spf.srijyx^2+2*spf.srijyy^2+2*spf.srijyz^2+2*spf.srijzx^2+2*spf.srijzy^2+2*spf.srijzz^2+eps) | 1/s | Shear rate | Domain 3 |  |
| spf.rr | sqrt(2*spf.rrijxx^2+2*spf.rrijxy^2+2*spf.rrijxz^2+2*spf.rrijyx^2+2*spf.rrijyy^2+2*spf.rrijyz^2+2*spf.rrijzx^2+2*spf.rrijzy^2+2*spf.rrijzz^2+eps) | 1/s | Rotation rate | Domain 3 |  |
| spf.divu | ux+vy+wz | 1/s | Divergence of velocity field | Domain 3 |  |
| spf.Fx | 0 | N/m³ | Volume force, x component | Domain 3 | + operation |
| spf.Fy | 0 | N/m³ | Volume force, y component | Domain 3 | + operation |
| spf.Fz | 0 | N/m³ | Volume force, z component | Domain 3 | + operation |
| spf.U | sqrt(u^2+v^2+w^2) | m/s | Velocity magnitude | Domain 3 |  |
| spf.vorticityx | wy-vz | 1/s | Vorticity field, x component | Domain 3 |  |
| spf.vorticityy | -wx+uz | 1/s | Vorticity field, y component | Domain 3 |  |
| spf.vorticityz | vx-uy | 1/s | Vorticity field, z component | Domain 3 |  |
| spf.vort_magn | sqrt(spf.vorticityx^2+spf.vorticityy^2+spf.vorticityz^2) | 1/s | Vorticity magnitude | Domain 3 |  |
| spf.cellRe | 0.25*spf.rho*sqrt(emetric_spatial(u-d(x,TIME),v-d(y,TIME),w-d(z,TIME))/emetric2_spatial)/spf.mu | 1 | Cell Reynolds number | Domain 3 |  |
| spf.nu | spf.mu/spf.rho | m²/s | Kinematic viscosity | Domain 3 |  |
| spf.betaT | 0 | 1/Pa | Isothermal compressibility coefficient | Domain 3 |  |
| spf.Qm | 0 | kg/(m³·s) | Source term | Domain 3 | + operation |
| spf.Fgtotx | 0 | N/m³ | Gravity force, x component | Domain 3 | + operation |
| spf.Fgtoty | 0 | N/m³ | Gravity force, y component | Domain 3 | + operation |
| spf.Fgtotz | 0 | N/m³ | Gravity force, z component | Domain 3 | + operation |
| spf.mu_eff | spf.mu+spf.muT | Pa·s | Dynamic viscosity | Domain 3 |  |
| spf.muT | 0 | Pa·s | Turbulent dynamic viscosity | Domain 3 |  |
| spf.T_stressx | spf.K_stressx-p*spf.nxmesh | N/m² | Total stress, x component | Boundaries 10–15 | + operation |
| spf.T_stressy | spf.K_stressy-p*spf.nymesh | N/m² | Total stress, y component | Boundaries 10–15 | + operation |
| spf.T_stressz | spf.K_stressz-p*spf.nzmesh | N/m² | Total stress, z component | Boundaries 10–15 | + operation |
| spf.K_stressx | spf.mu_eff*(2*ux*spf.nxmesh+(uy+vx)*spf.nymesh+(uz+wx)*spf.nzmesh) | N/m² | Viscous stress, x component | Boundaries 10–15 | + operation |
| spf.K_stressy | spf.mu_eff*((vx+uy)*spf.nxmesh+2*vy*spf.nymesh+(vz+wy)*spf.nzmesh) | N/m² | Viscous stress, y component | Boundaries 10–15 | + operation |
| spf.K_stressz | spf.mu_eff*((wx+uz)*spf.nxmesh+(wy+vz)*spf.nymesh+2*wz*spf.nzmesh) | N/m² | Viscous stress, z component | Boundaries 10–15 | + operation |
| spf.K_stress_tensorxx | 2*spf.mu_eff*ux | N/m² | Viscous stress tensor, xx component | Domain 3 | + operation |
| spf.K_stress_tensoryx | spf.mu_eff*(vx+uy) | N/m² | Viscous stress tensor, yx component | Domain 3 | + operation |
| spf.K_stress_tensorzx | spf.mu_eff*(wx+uz) | N/m² | Viscous stress tensor, zx component | Domain 3 | + operation |
| spf.K_stress_tensorxy | spf.mu_eff*(uy+vx) | N/m² | Viscous stress tensor, xy component | Domain 3 | + operation |
| spf.K_stress_tensoryy | 2*spf.mu_eff*vy | N/m² | Viscous stress tensor, yy component | Domain 3 | + operation |
| spf.K_stress_tensorzy | spf.mu_eff*(wy+vz) | N/m² | Viscous stress tensor, zy component | Domain 3 | + operation |
| spf.K_stress_tensorxz | spf.mu_eff*(uz+wx) | N/m² | Viscous stress tensor, xz component | Domain 3 | + operation |
| spf.K_stress_tensoryz | spf.mu_eff*(vz+wy) | N/m² | Viscous stress tensor, yz component | Domain 3 | + operation |
| spf.K_stress_tensorzz | 2*spf.mu_eff*wz | N/m² | Viscous stress tensor, zz component | Domain 3 | + operation |
| spf.K_stress_tensor_testxx | 2*spf.mu_eff*test(ux) | N/m² | Viscous stress tensor test, xx component | Domain 3 | + operation |
| spf.K_stress_tensor_testyx | spf.mu_eff*(test(vx)+test(uy)) | N/m² | Viscous stress tensor test, yx component | Domain 3 | + operation |
| spf.K_stress_tensor_testzx | spf.mu_eff*(test(wx)+test(uz)) | N/m² | Viscous stress tensor test, zx component | Domain 3 | + operation |
| spf.K_stress_tensor_testxy | spf.mu_eff*(test(uy)+test(vx)) | N/m² | Viscous stress tensor test, xy component | Domain 3 | + operation |
| spf.K_stress_tensor_testyy | 2*spf.mu_eff*test(vy) | N/m² | Viscous stress tensor test, yy component | Domain 3 | + operation |
| spf.K_stress_tensor_testzy | spf.mu_eff*(test(wy)+test(vz)) | N/m² | Viscous stress tensor test, zy component | Domain 3 | + operation |
| spf.K_stress_tensor_testxz | spf.mu_eff*(test(uz)+test(wx)) | N/m² | Viscous stress tensor test, xz component | Domain 3 | + operation |
| spf.K_stress_tensor_testyz | spf.mu_eff*(test(vz)+test(wy)) | N/m² | Viscous stress tensor test, yz component | Domain 3 | + operation |
| spf.K_stress_tensor_testzz | 2*spf.mu_eff*test(wz) | N/m² | Viscous stress tensor test, zz component | Domain 3 | + operation |
| spf.upwind_helpx | u-d(x,TIME) | m/s | Upwind term, x component | Domain 3 | + operation |
| spf.upwind_helpy | v-d(y,TIME) | m/s | Upwind term, y component | Domain 3 | + operation |
| spf.upwind_helpz | w-d(z,TIME) | m/s | Upwind term, z component | Domain 3 | + operation |
| spf.tau_vdxx | 2*spf.mu*spf.srijxx | Pa | Viscous stress tensor, xx component | Domain 3 | + operation |
| spf.tau_vdyx | 2*spf.mu*spf.srijyx | Pa | Viscous stress tensor, yx component | Domain 3 | + operation |
| spf.tau_vdzx | 2*spf.mu*spf.srijzx | Pa | Viscous stress tensor, zx component | Domain 3 | + operation |
| spf.tau_vdxy | 2*spf.mu*spf.srijxy | Pa | Viscous stress tensor, xy component | Domain 3 | + operation |
| spf.tau_vdyy | 2*spf.mu*spf.srijyy | Pa | Viscous stress tensor, yy component | Domain 3 | + operation |
| spf.tau_vdzy | 2*spf.mu*spf.srijzy | Pa | Viscous stress tensor, zy component | Domain 3 | + operation |
| spf.tau_vdxz | 2*spf.mu*spf.srijxz | Pa | Viscous stress tensor, xz component | Domain 3 | + operation |
| spf.tau_vdyz | 2*spf.mu*spf.srijyz | Pa | Viscous stress tensor, yz component | Domain 3 | + operation |
| spf.tau_vdzz | 2*spf.mu*spf.srijzz | Pa | Viscous stress tensor, zz component | Domain 3 | + operation |
| spf.Qvd | spf.tau_vdxx*ux+spf.tau_vdxy*uy+spf.tau_vdxz*uz+spf.tau_vdyx*vx+spf.tau_vdyy*vy+spf.tau_vdyz*vz+spf.tau_vdzx*wx+spf.tau_vdzy*wy+spf.tau_vdzz*wz | W/m³ | Viscous dissipation | Domain 3 | + operation |
| spf.epsilon_p | 1 | 1 | Porosity | Domain 3 |  |
| spf.Fst_tensorxx | 0 | N/m² | Surface tension force, xx component | Domain 3 | + operation |
| spf.Fst_tensoryx | 0 | N/m² | Surface tension force, yx component | Domain 3 | + operation |
| spf.Fst_tensorzx | 0 | N/m² | Surface tension force, zx component | Domain 3 | + operation |
| spf.Fst_tensorxy | 0 | N/m² | Surface tension force, xy component | Domain 3 | + operation |
| spf.Fst_tensoryy | 0 | N/m² | Surface tension force, yy component | Domain 3 | + operation |
| spf.Fst_tensorzy | 0 | N/m² | Surface tension force, zy component | Domain 3 | + operation |
| spf.Fst_tensorxz | 0 | N/m² | Surface tension force, xz component | Domain 3 | + operation |
| spf.Fst_tensoryz | 0 | N/m² | Surface tension force, yz component | Domain 3 | + operation |
| spf.Fst_tensorzz | 0 | N/m² | Surface tension force, zz component | Domain 3 | + operation |
| spf.continuityEquation | spf.rho*spf.divu | kg/(m³·s) | Continuity equation | Domain 3 |  |
| spf.contCoeff | spf.rho | kg/m³ | Help variable | Domain 3 |  |
| spf.res_u | spf.rho*(uTIME-d(u,x)*d(x,TIME)-d(u,y)*d(y,TIME)-d(u,z)*d(z,TIME))+px+spf.rho*u*ux+spf.rho*v*uy+spf.rho*w*uz-(d(2*ux,x)+d(uy+vx,y)+d(uz+wx,z))*spf.mu-spf.Fx | N/m³ | Equation residual | Domain 3 |  |
| spf.res_v | spf.rho*(vTIME-d(v,x)*d(x,TIME)-d(v,y)*d(y,TIME)-d(v,z)*d(z,TIME))+spf.rho*u*vx+py+spf.rho*v*vy+spf.rho*w*vz-(d(vx+uy,x)+d(2*vy,y)+d(vz+wy,z))*spf.mu-spf.Fy | N/m³ | Equation residual | Domain 3 |  |
| spf.res_w | spf.rho*(wTIME-d(w,x)*d(x,TIME)-d(w,y)*d(y,TIME)-d(w,z)*d(z,TIME))+spf.rho*u*wx+spf.rho*v*wy+pz+spf.rho*w*wz-(d(wx+uz,x)+d(wy+vz,y)+d(2*wz,z))*spf.mu-spf.Fz | N/m³ | Equation residual | Domain 3 |  |
| spf.res_p | spf.rho*spf.divu | kg/(m³·s) | Pressure equation residual | Domain 3 |  |

#### Shape functions

| **Name** | **Shape function** | **Unit** | **Description** | **Shape frame** | **Selection** |
| --- | --- | --- | --- | --- | --- |
| u | Lagrange (Linear) | m/s | Velocity field, x component | Spatial | Domain 3 |
| v | Lagrange (Linear) | m/s | Velocity field, y component | Spatial | Domain 3 |
| w | Lagrange (Linear) | m/s | Velocity field, z component | Spatial | Domain 3 |
| p | Lagrange (Linear) | Pa | Pressure | Spatial | Domain 3 |

#### Weak expressions

| **Weak expression** | **Integration order** | **Integration frame** | **Selection** |
| --- | --- | --- | --- |
| spf.rho*(-(uTIME-ux*d(x,TIME)-uy*d(y,TIME)-uz*d(z,TIME))*test(u)-(vTIME-vx*d(x,TIME)-vy*d(y,TIME)-vz*d(z,TIME))*test(v)-(wTIME-wx*d(x,TIME)-wy*d(y,TIME)-wz*d(z,TIME))*test(w)) | 2 | Spatial | Domain 3 |
| (p-spf.K_stress_tensorxx)*test(ux)-spf.K_stress_tensorxy*test(uy)-spf.K_stress_tensorxz*test(uz)-spf.K_stress_tensoryx*test(vx)+(p-spf.K_stress_tensoryy)*test(vy)-spf.K_stress_tensoryz*test(vz)-spf.K_stress_tensorzx*test(wx)-spf.K_stress_tensorzy*test(wy)+(p-spf.K_stress_tensorzz)*test(wz) | 2 | Spatial | Domain 3 |
| spf.Fx*test(u)+spf.Fy*test(v)+spf.Fz*test(w) | 2 | Spatial | Domain 3 |
| spf.rho*(-(d(u,x)*u+d(u,y)*v+d(u,z)*w)*test(u)-(d(v,x)*u+d(v,y)*v+d(v,z)*w)*test(v)-(d(w,x)*u+d(w,y)*v+d(w,z)*w)*test(w)) | 2 | Spatial | Domain 3 |
| -spf.continuityEquation*test(p) | 2 | Spatial | Domain 3 |
| spf.streamlinens | 2 | Spatial | Domain 3 |
| spf.crosswindns | 2 | Spatial | Domain 3 |

- - 1. Initial Values 1


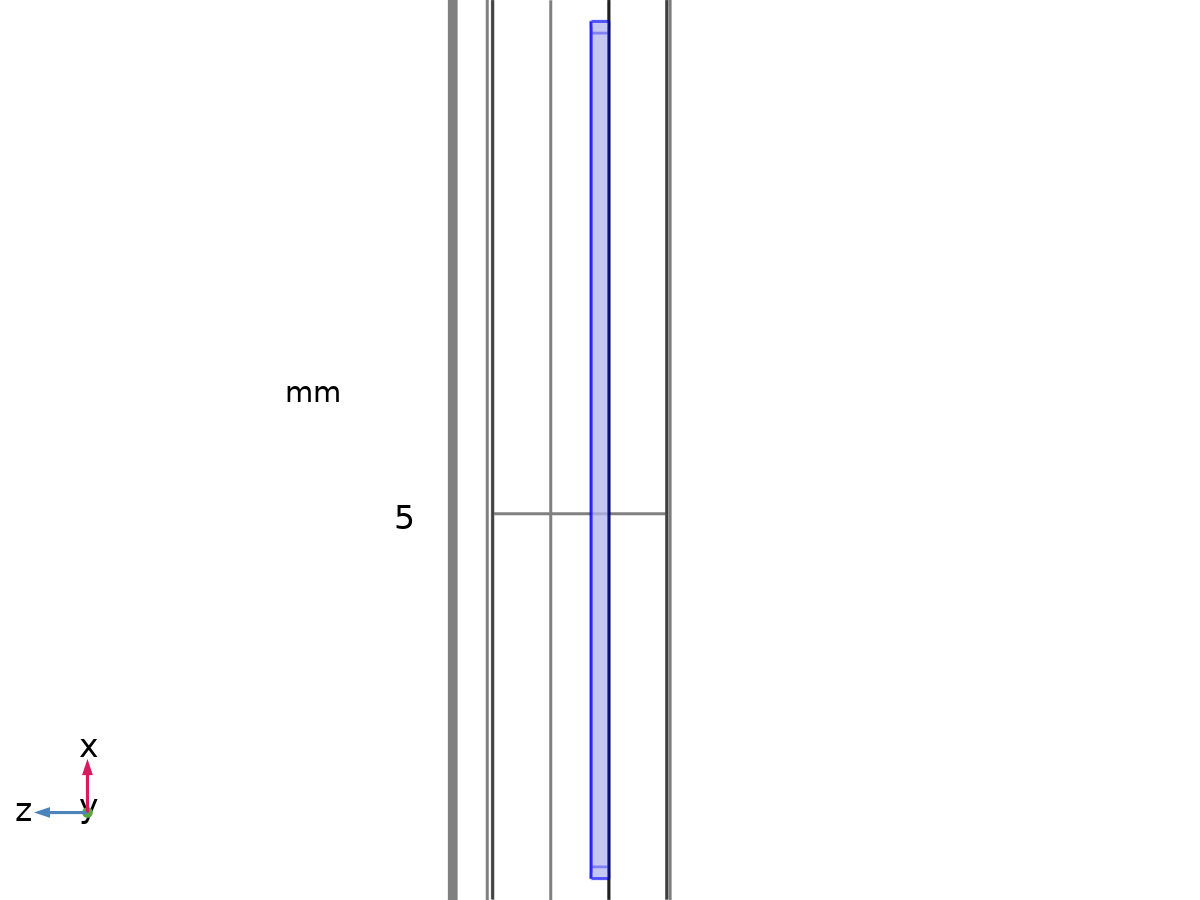


Initial Values 1

Selection

| Geometric entity level | Domain |
| --- | --- |
| Selection | Geometry geom1: Dimension 3: All domains |

#### Initial values

Settings

| **Description** | **Value** |
| --- | --- |
| Velocity field, x component | 0 |
| Velocity field, y component | 0 |
| Velocity field, z component | 0 |
| Pressure | 0.05 |

#### Coordinate system selection

Settings

| **Description** | **Value** |
| --- | --- |
| Coordinate system | Global coordinate system |

#### Variables

| **Name** | **Expression** | **Unit** | **Description** | **Selection** |
| --- | --- | --- | --- | --- |
| spf.u_initx | 0 | m/s | Velocity field, x component | Domain 3 |
| spf.u_inity | 0 | m/s | Velocity field, y component | Domain 3 |
| spf.u_initz | 0 | m/s | Velocity field, z component | Domain 3 |
| spf.p_init | 0.05 | Pa | Pressure | Domain 3 |

- - 1. Wall 1


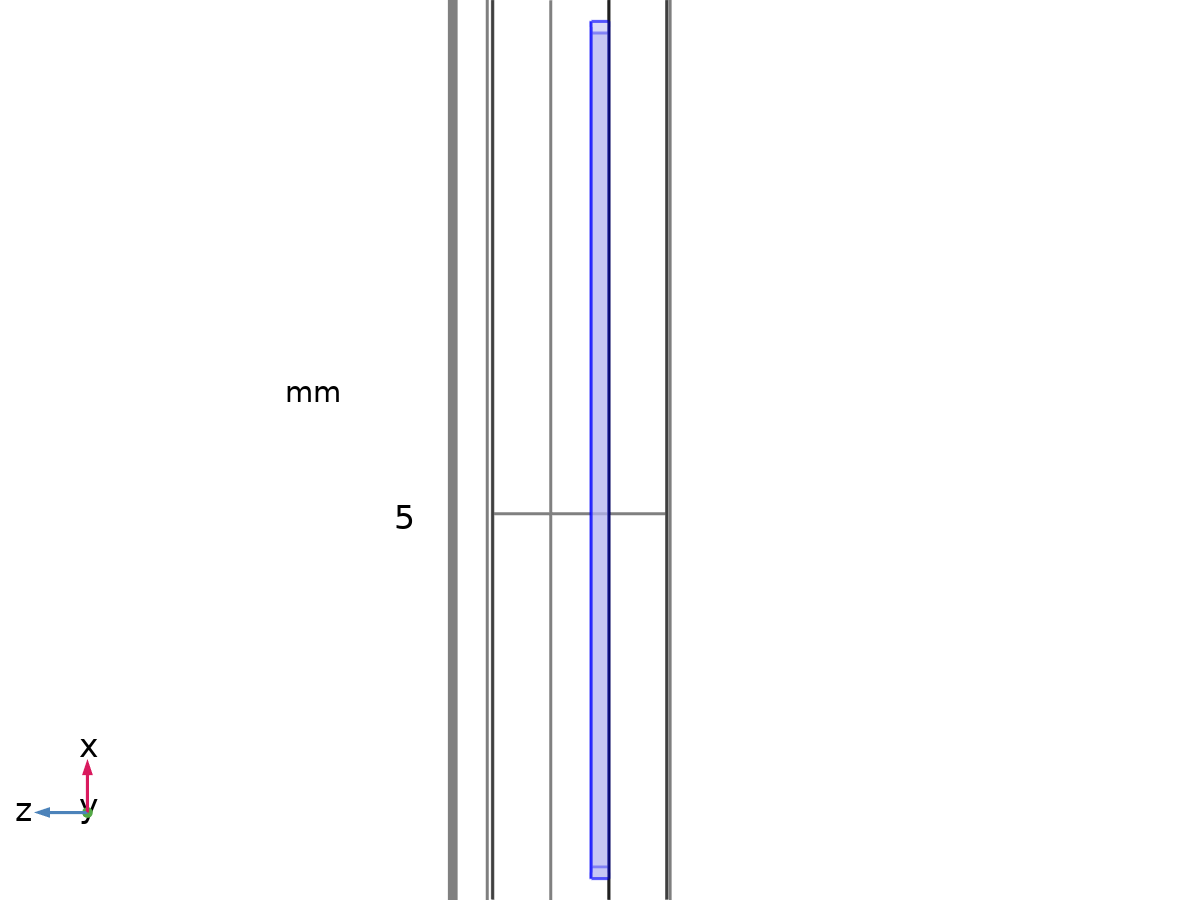


Wall 1

Selection

| Geometric entity level | Boundary |
| --- | --- |
| Selection | Geometry geom1: Dimension 2: All boundaries |

Equations


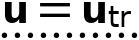


#### Boundary condition

Settings

| **Description** | **Value** |
| --- | --- |
| Wall condition | No slip |

#### Wall movement

Settings

| **Description** | **Value** |
| --- | --- |
| Translational velocity | Automatic from frame |
| Sliding wall | Off |

#### Variables

| **Name** | **Expression** | **Unit** | **Description** | **Selection** | **Details** |
| --- | --- | --- | --- | --- | --- |
| spf.ubndx | spf.utrx+spf.usx | m/s | Velocity at boundary, x component | Boundaries 10–14 |  |
| spf.ubndy | spf.utry+spf.usy | m/s | Velocity at boundary, y component | Boundaries 10–14 |  |
| spf.ubndz | spf.utrz+spf.usz | m/s | Velocity at boundary, z component | Boundaries 10–14 |  |
| spf.usx | 0 | m/s | Velocity of sliding wall, x component | Boundaries 10–14 |  |
| spf.usy | 0 | m/s | Velocity of sliding wall, y component | Boundaries 10–14 |  |
| spf.usz | 0 | m/s | Velocity of sliding wall, z component | Boundaries 10–14 |  |
| spf.utrx | d(x,TIME) | m/s | Velocity of moving wall, x component | Boundaries 10–14 |  |
| spf.utry | d(y,TIME) | m/s | Velocity of moving wall, y component | Boundaries 10–14 |  |
| spf.utrz | d(z,TIME) | m/s | Velocity of moving wall, z component | Boundaries 10–14 |  |
| spf.uLeakagex | 0 | m/s | Leakage velocity, x component | Boundaries 10–14 | + operation |
| spf.uLeakagey | 0 | m/s | Leakage velocity, y component | Boundaries 10–14 | + operation |
| spf.uLeakagez | 0 | m/s | Leakage velocity, z component | Boundaries 10–14 | + operation |
| spf.noSlipWall | 1 | 1 | Help variable | Boundaries 10–14 |  |
| spf.u_herex | u | m/s | Intermediate variable, x component | Boundaries 10–14 |  |
| spf.u_herey | v | m/s | Intermediate variable, y component | Boundaries 10–14 |  |
| spf.u_herez | w | m/s | Intermediate variable, z component | Boundaries 10–14 |  |
| spf.u_therex | spf.ubndx+spf.uLeakagex | m/s | Intermediate variable, x component | Boundaries 10–14 |  |
| spf.u_therey | spf.ubndy+spf.uLeakagey | m/s | Intermediate variable, y component | Boundaries 10–14 |  |
| spf.u_therez | spf.ubndz+spf.uLeakagez | m/s | Intermediate variable, z component | Boundaries 10–14 |  |
| spf.KStressn_avx | spf.K_stress_tensorxx*spf.nxmesh+spf.K_stress_tensorxy*spf.nymesh+spf.K_stress_tensorxz*spf.nzmesh | N/m² | Average viscous stress, x component | Boundaries 10–14 |  |
| spf.KStressn_avy | spf.K_stress_tensoryx*spf.nxmesh+spf.K_stress_tensoryy*spf.nymesh+spf.K_stress_tensoryz*spf.nzmesh | N/m² | Average viscous stress, y component | Boundaries 10–14 |  |
| spf.KStressn_avz | spf.K_stress_tensorzx*spf.nxmesh+spf.K_stress_tensorzy*spf.nymesh+spf.K_stress_tensorzz*spf.nzmesh | N/m² | Average viscous stress, z component | Boundaries 10–14 |  |
| spf.KStressTestn_avx | spf.K_stress_tensor_testxx*spf.nxmesh+spf.K_stress_tensor_testxy*spf.nymesh+spf.K_stress_tensor_testxz*spf.nzmesh | N/m² | Average viscous stress, x component | Boundaries 10–14 |  |
| spf.KStressTestn_avy | spf.K_stress_tensor_testyx*spf.nxmesh+spf.K_stress_tensor_testyy*spf.nymesh+spf.K_stress_tensor_testyz*spf.nzmesh | N/m² | Average viscous stress, y component | Boundaries 10–14 |  |
| spf.KStressTestn_avz | spf.K_stress_tensor_testzx*spf.nxmesh+spf.K_stress_tensor_testzy*spf.nymesh+spf.K_stress_tensor_testzz*spf.nzmesh | N/m² | Average viscous stress, z component | Boundaries 10–14 |  |
| spf.ujumpx | spf.u_herex-spf.u_therex | m/s | Velocity jump, x component | Boundaries 10–14 |  |
| spf.ujumpy | spf.u_herey-spf.u_therey | m/s | Velocity jump, y component | Boundaries 10–14 |  |
| spf.ujumpz | spf.u_herez-spf.u_therez | m/s | Velocity jump, z component | Boundaries 10–14 |  |
| spf.meshVol | meshvol_spatial | m² |  | Boundaries 10–14 |  |
| spf.meshVolInt | down(meshvol_spatial) | m³ | Volume of interior mesh element | Boundaries 10–14 |  |
| spf.c_here | 36*nojac(down(spf.mu+spf.muT))*spf.meshVol/spf.meshVolInt | Pa·s/m | Intermediate variable | Boundaries 10–14 |  |
| spf.sigma_dg_ns | 4*spf.c_here | Pa·s/m |  | Boundaries 10–14 |  |
| spf.rhoFace | down(spf.rho) | kg/m³ | Density face value | Boundaries 10–14 |  |
| spf.contCoeffFace | down(spf.contCoeff) | kg/m³ | Help variable | Boundaries 10–14 |  |
| spf.umxTnFace | (spf.upwind_helpx*spf.nxmesh+spf.upwind_helpy*spf.nymesh+spf.upwind_helpz*spf.nzmesh<0)*(spf.upwind_helpx*spf.nxmesh+spf.upwind_helpy*spf.nymesh+spf.upwind_helpz*spf.nzmesh) | m/s | Relative velocity on face | Boundaries 10–14 |  |
| spf.upwind_ns | spf.rhoFace*spf.umxTnFace*(spf.ujumpx*test(spf.u_herex)+spf.ujumpy*test(spf.u_herey)+spf.ujumpz*test(spf.u_herez)) | W/m² | Upwind term | Boundaries 10–14 |  |
| spf.upwindCont | spf.contCoeffFace*(spf.ujumpx*spf.nxmesh+spf.ujumpy*spf.nymesh+spf.ujumpz*spf.nzmesh)*test(p) | kg²/(m³·s³) | Upwind term for continuity equation | Boundaries 10–14 |  |
| spf.pFace | p | Pa | Pressure face value | Boundaries 10–14 |  |
| spf.consFlux | spf.pFace*(-test(spf.u_herex)*spf.nxmesh-test(spf.u_herey)*spf.nymesh-test(spf.u_herez)*spf.nzmesh) | W/m² | Conservative flux | Boundaries 10–14 | + operation |

#### Weak expressions

| **Weak expression** | **Integration order** | **Integration frame** | **Selection** |
| --- | --- | --- | --- |
| spf.KStressn_avx*test(spf.u_herex)+spf.KStressn_avy*test(spf.u_herey)+spf.KStressn_avz*test(spf.u_herez)+spf.KStressTestn_avx*spf.ujumpx+spf.KStressTestn_avy*spf.ujumpy+spf.KStressTestn_avz*spf.ujumpz-spf.sigma_dg_ns*spf.ujumpx*test(spf.u_herex)-spf.sigma_dg_ns*spf.ujumpy*test(spf.u_herey)-spf.sigma_dg_ns*spf.ujumpz*test(spf.u_herez)+spf.upwind_ns+spf.upwindCont+spf.consFlux | 2 | Spatial | Boundaries 10–14 |

#### Constraints

| **Constraint** | **Constraint force** | **Shape function** | **Selection** | **Details** |
| --- | --- | --- | --- | --- |
| -u+spf.ubndx+spf.uLeakagex | test(-u) | Lagrange (Linear) | No boundaries | Elemental |
| -v+spf.ubndy+spf.uLeakagey | test(-v) | Lagrange (Linear) | No boundaries | Elemental |
| -w+spf.ubndz+spf.uLeakagez | test(-w) | Lagrange (Linear) | No boundaries | Elemental |

- - 1. Inlet 1


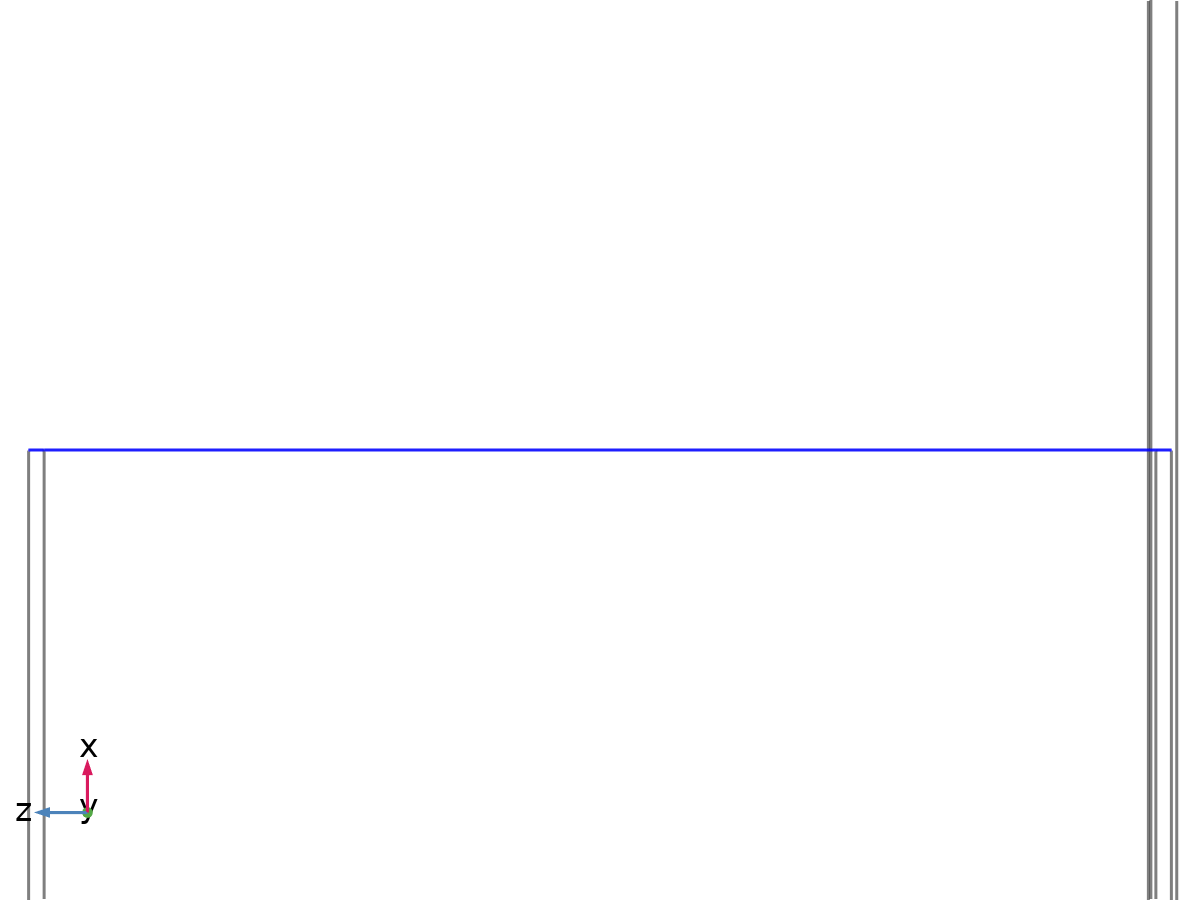


Inlet 1

Selection

| Geometric entity level | Boundary |
| --- | --- |
| Selection | Geometry geom1: Dimension 2: Boundary 15 |

Equations


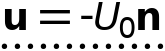


#### Boundary condition

Settings

| **Description** | **Value** |
| --- | --- |
| Boundary condition | Velocity |

#### Velocity

Settings

| **Description** | **Value** |
| --- | --- |
| Velocity field componentwise | Normal inflow velocity |
| Normal inflow velocity | 0.009 |

#### Variables

| **Name** | **Expression** | **Unit** | **Description** | **Selection** |
| --- | --- | --- | --- | --- |
| spf.ubndx | -nojac(spf.nxmesh)*spf.U0in | m/s | Velocity at boundary, x component | Boundary 15 |
| spf.ubndy | -nojac(spf.nymesh)*spf.U0in | m/s | Velocity at boundary, y component | Boundary 15 |
| spf.ubndz | -nojac(spf.nzmesh)*spf.U0in | m/s | Velocity at boundary, z component | Boundary 15 |
| spf.U0in | 0.009 | m/s | Normal inflow velocity | Boundary 15 |
| spf.inl1.Te0xx | 0 |  | Viscoelastic stress tensor, xx component | Boundary 15 |
| spf.inl1.Te0xy | 0 |  | Viscoelastic stress tensor, xy component | Boundary 15 |
| spf.inl1.Te0xz | 0 |  | Viscoelastic stress tensor, xz component | Boundary 15 |
| spf.inl1.Te0yy | 0 |  | Viscoelastic stress tensor, yy component | Boundary 15 |
| spf.inl1.Te0yz | 0 |  | Viscoelastic stress tensor, yz component | Boundary 15 |
| spf.inl1.Te0zz | 0 |  | Viscoelastic stress tensor, zz component | Boundary 15 |

#### Constraints

| **Constraint** | **Constraint force** | **Shape function** | **Selection** | **Details** |
| --- | --- | --- | --- | --- |
| -u+spf.ubndx | test(-u+spf.ubndx) | Lagrange (Linear) | Boundary 15 | Elemental |
| -v+spf.ubndy | test(-v+spf.ubndy) | Lagrange (Linear) | Boundary 15 | Elemental |
| -w+spf.ubndz | test(-w+spf.ubndz) | Lagrange (Linear) | Boundary 15 | Elemental |

- 1. Solid Mechanics

Used products

| COMSOL Multiphysics |
| --- |
| Nonlinear Structural Materials Module |
| Structural Mechanics Module |


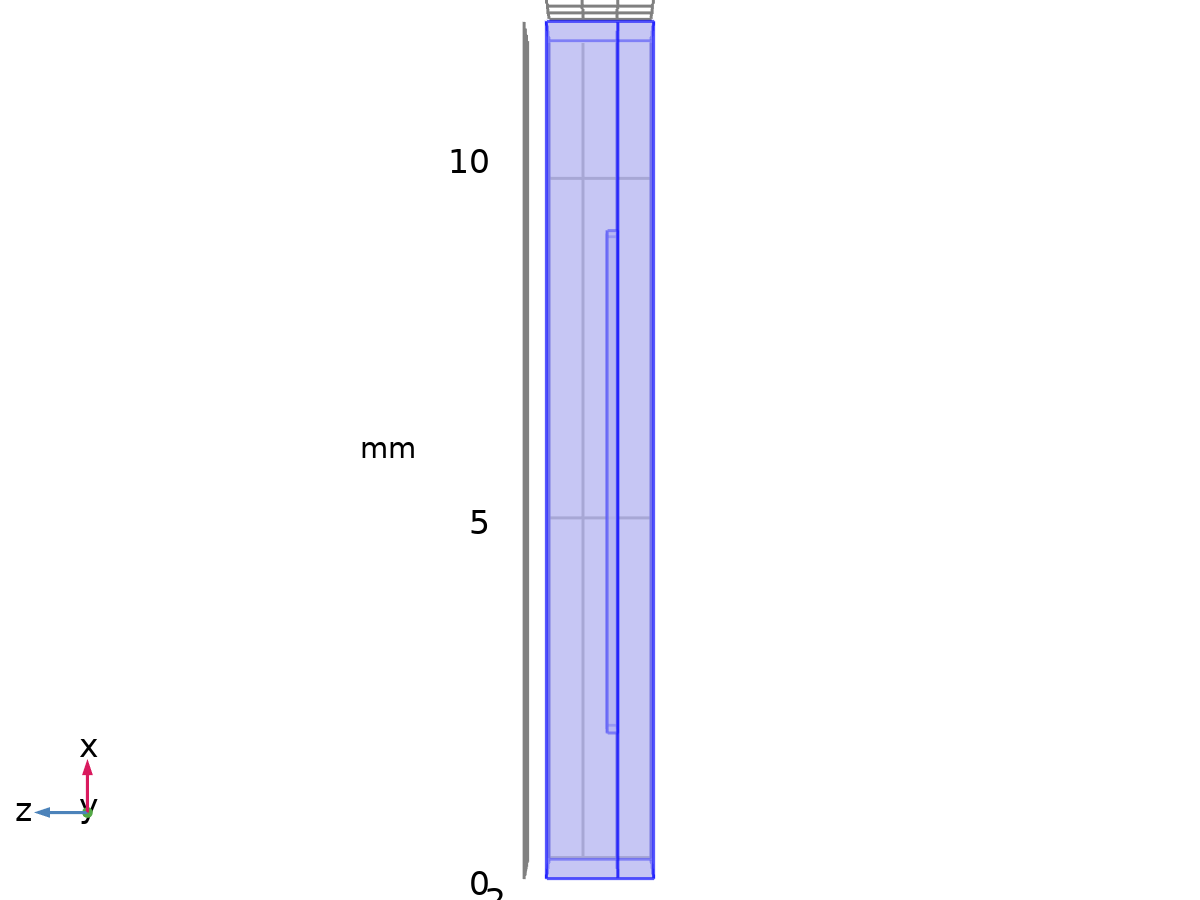


Solid Mechanics

Selection

| Geometric entity level | Domain |
| --- | --- |
| Selection | Geometry geom1: Dimension 3: Domains 1–2 |

Equations


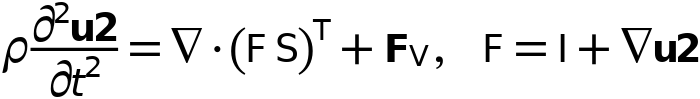


- - 1. Interface settings

#### Physics symbols

Settings

| **Description** | **Value** |
| --- | --- |
| Enable physics symbols | Off |

#### Discretization

Settings

| **Description** | **Value** |
| --- | --- |
| Displacement field | Quadratic Lagrange |

#### Typical wave speed for perfectly matched layers

Settings

| **Description** | **Value** |
| --- | --- |
| Typical wave speed for perfectly matched layers | solid.cp |

- - 1. Variables

| **Name** | **Expression** | **Unit** | **Description** | **Selection** | **Details** |
| --- | --- | --- | --- | --- | --- |
| solid.nX | nX | 1 | Normal vector, X component | Boundary 6 |  |
| solid.nY | nY | 1 | Normal vector, Y component | Boundary 6 |  |
| solid.nZ | nZ | 1 | Normal vector, Z component | Boundary 6 |  |
| solid.nX | unX | 1 | Normal vector, X component | Boundaries 10–15 |  |
| solid.nY | unY | 1 | Normal vector, Y component | Boundaries 10–15 |  |
| solid.nZ | unZ | 1 | Normal vector, Z component | Boundaries 10–15 |  |
| solid.nX | dnX | 1 | Normal vector, X component | Boundaries 1–5, 7–9, 16–17 |  |
| solid.nY | dnY | 1 | Normal vector, Y component | Boundaries 1–5, 7–9, 16–17 |  |
| solid.nZ | dnZ | 1 | Normal vector, Z component | Boundaries 1–5, 7–9, 16–17 |  |
| solid.nx | nx | 1 | Normal vector, x component | Boundary 6 |  |
| solid.ny | ny | 1 | Normal vector, y component | Boundary 6 |  |
| solid.nz | nz | 1 | Normal vector, z component | Boundary 6 |  |
| solid.nx | unx | 1 | Normal vector, x component | Boundaries 10–15 |  |
| solid.ny | uny | 1 | Normal vector, y component | Boundaries 10–15 |  |
| solid.nz | unz | 1 | Normal vector, z component | Boundaries 10–15 |  |
| solid.nx | dnx | 1 | Normal vector, x component | Boundaries 1–5, 7–9, 16–17 |  |
| solid.ny | dny | 1 | Normal vector, y component | Boundaries 1–5, 7–9, 16–17 |  |
| solid.nz | dnz | 1 | Normal vector, z component | Boundaries 1–5, 7–9, 16–17 |  |
| solid.nXmesh | nXmesh | 1 | Normal vector (mesh), X component | Boundary 6 |  |
| solid.nYmesh | nYmesh | 1 | Normal vector (mesh), Y component | Boundary 6 |  |
| solid.nZmesh | nZmesh | 1 | Normal vector (mesh), Z component | Boundary 6 |  |
| solid.nXmesh | unXmesh | 1 | Normal vector (mesh), X component | Boundaries 10–15 |  |
| solid.nYmesh | unYmesh | 1 | Normal vector (mesh), Y component | Boundaries 10–15 |  |
| solid.nZmesh | unZmesh | 1 | Normal vector (mesh), Z component | Boundaries 10–15 |  |
| solid.nXmesh | dnXmesh | 1 | Normal vector (mesh), X component | Boundaries 1–5, 7–9, 16–17 |  |
| solid.nYmesh | dnYmesh | 1 | Normal vector (mesh), Y component | Boundaries 1–5, 7–9, 16–17 |  |
| solid.nZmesh | dnZmesh | 1 | Normal vector (mesh), Z component | Boundaries 1–5, 7–9, 16–17 |  |
| solid.nxmesh | nxmesh | 1 | Normal vector (mesh), x component | Boundary 6 |  |
| solid.nymesh | nymesh | 1 | Normal vector (mesh), y component | Boundary 6 |  |
| solid.nzmesh | nzmesh | 1 | Normal vector (mesh), z component | Boundary 6 |  |
| solid.nxmesh | unxmesh | 1 | Normal vector (mesh), x component | Boundaries 10–15 |  |
| solid.nymesh | unymesh | 1 | Normal vector (mesh), y component | Boundaries 10–15 |  |
| solid.nzmesh | unzmesh | 1 | Normal vector (mesh), z component | Boundaries 10–15 |  |
| solid.nxmesh | dnxmesh | 1 | Normal vector (mesh), x component | Boundaries 1–5, 7–9, 16–17 |  |
| solid.nymesh | dnymesh | 1 | Normal vector (mesh), y component | Boundaries 1–5, 7–9, 16–17 |  |
| solid.nzmesh | dnzmesh | 1 | Normal vector (mesh), z component | Boundaries 1–5, 7–9, 16–17 |  |
| solid.refpntx | 0 | m | Reference point for moment computation, x component | Global |  |
| solid.refpnty | 0 | m | Reference point for moment computation, y component | Global |  |
| solid.refpntz | 0 | m | Reference point for moment computation, z component | Global |  |
| solid.cref | solid.cp | m/s | Typical wave speed for perfectly matched layers | Domains 1–2 |  |
| solid.isGeomNon | 1 | 1 | Geometric nonlinearity variable | Global |  |
| solid.RFtotalx | solid.sumreaction(solid.RFx)+solid.RFfsx+solid.RFfdx | N | Total reaction force, x component | Global | + operation |
| solid.RFtotaly | solid.sumreaction(solid.RFy)+solid.RFfsy+solid.RFfdy | N | Total reaction force, y component | Global | + operation |
| solid.RFtotalz | solid.sumreaction(solid.RFz)+solid.RFfsz+solid.RFfdz | N | Total reaction force, z component | Global | + operation |
| solid.RMtotalx | solid.sumreaction(solid.RMx)+solid.RMmsx+solid.RMmdx | N·m | Total reaction moment, x component | Global | + operation |
| solid.RMtotaly | solid.sumreaction(solid.RMy)+solid.RMmsy+solid.RMmdy | N·m | Total reaction moment, y component | Global | + operation |
| solid.RMtotalz | solid.sumreaction(solid.RMz)+solid.RMmsz+solid.RMmdz | N·m | Total reaction moment, z component | Global | + operation |
| xt | d(x,TIME) | m/s | Mesh velocity, x component | Global |  |
| yt | d(y,TIME) | m/s | Mesh velocity, y component | Global |  |
| zt | d(z,TIME) | m/s | Mesh velocity, z component | Global |  |

- - 1. Linear Elastic Material 1


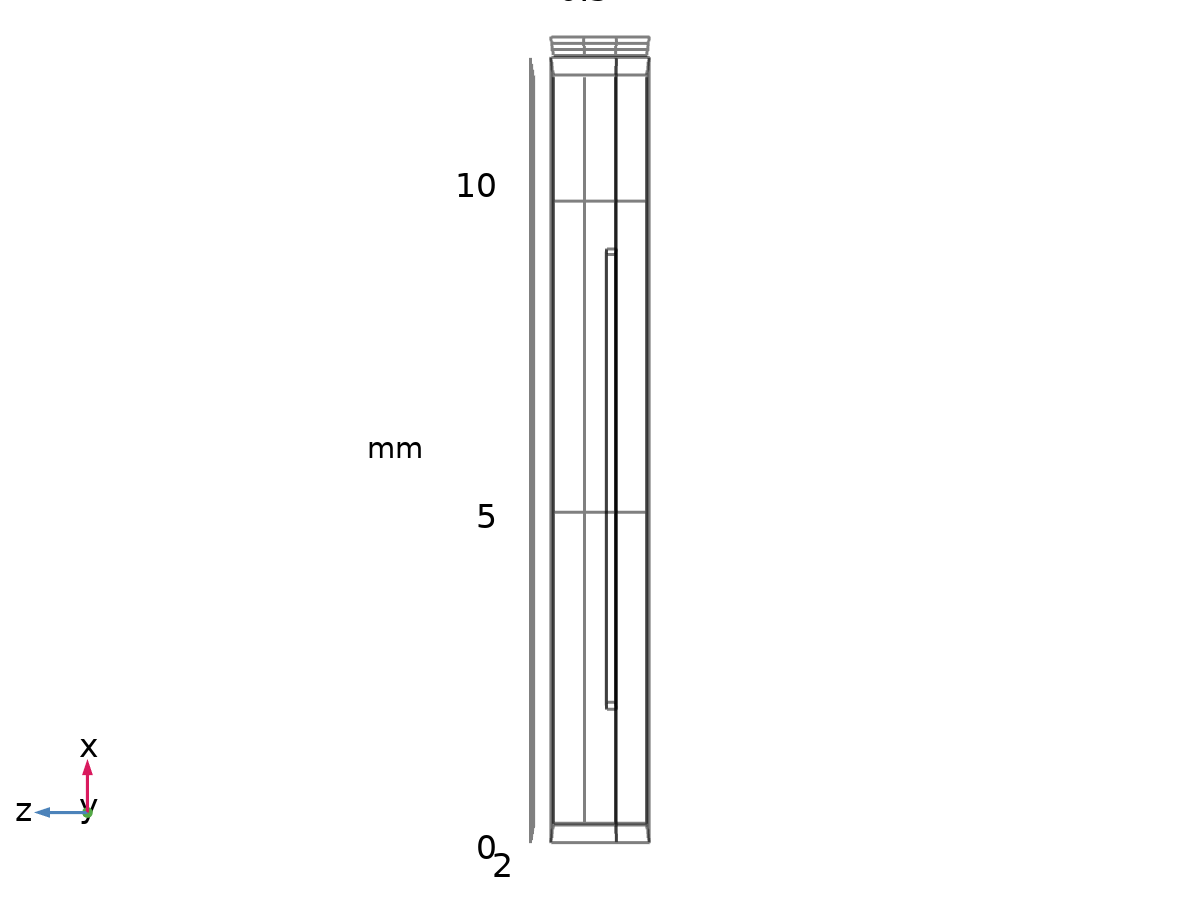


Linear Elastic Material 1

Selection

| Geometric entity level | Domain |
| --- | --- |
| Selection | Geometry geom1: Dimension 3: All domains |

Equations


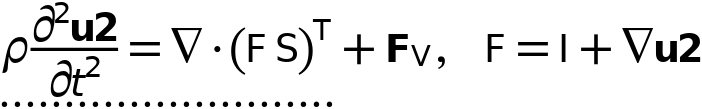


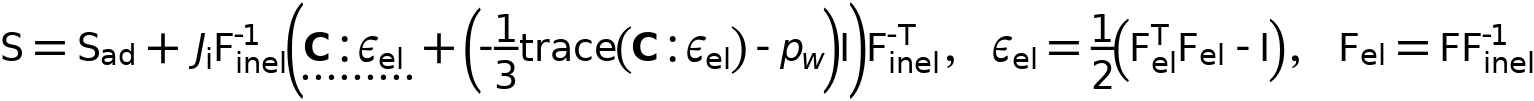


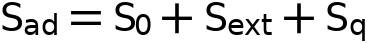


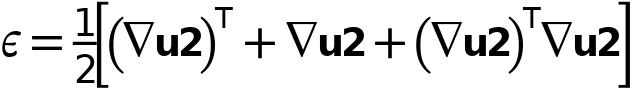


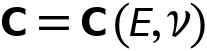


#### Linear elastic material

Settings

| **Description** | **Value** |
| --- | --- |
| Solid model | Isotropic |
| Specify | Young's modulus and Poisson's ratio |
| Young's modulus | From material |
| Poisson's ratio | From material |
| Density | From material |
| Use mixed formulation | Pressure formulation |

#### Geometric nonlinearity

Settings

| **Description** | **Value** |
| --- | --- |
| Force linear strains | Off |
| Additive strain decomposition | Off |

#### Coordinate system selection

Settings

| **Description** | **Value** |
| --- | --- |
| Coordinate system | Global coordinate system |

#### Model input

Settings

| **Description** | **Value** |
| --- | --- |
| Volume reference temperature | User defined |
| Volume reference temperature | 293.15[K] |
| Temperature | User defined |
| Temperature | 293.15[K] |

Used products

| COMSOL Multiphysics |
| --- |

#### Shape functions

| **Name** | **Shape function** | **Unit** | **Description** | **Shape frame** | **Selection** | **Details** |
| --- | --- | --- | --- | --- | --- | --- |
| u2 | Lagrange (Quadratic) | m | Displacement field, X component | Material | No domains |  |
| v2 | Lagrange (Quadratic) | m | Displacement field, Y component | Material | No domains |  |
| w2 | Lagrange (Quadratic) | m | Displacement field, Z component | Material | No domains |  |
| solid.pw | Lagrange (Linear) | N/m² | Auxiliary pressure | Material | No domains |  |
| solid.pw | Lagrange (Linear) | N/m² | Auxiliary pressure | Material | No boundaries | Slit |

- - 1. Free 1


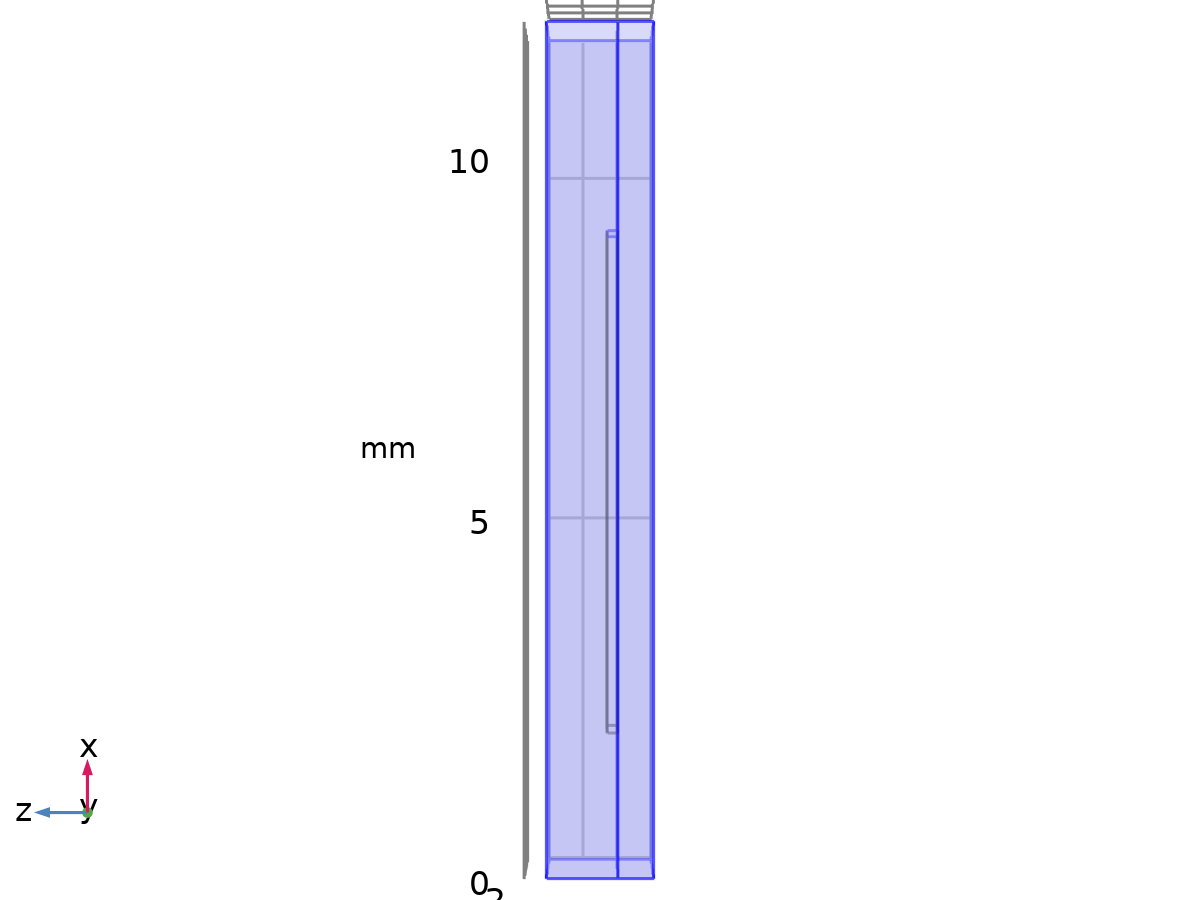


Free 1

Selection

| Geometric entity level | Boundary |
| --- | --- |
| Selection | Geometry geom1: Dimension 2: All boundaries |

Used products

| COMSOL Multiphysics |
| --- |

- - 1. Initial Values 1


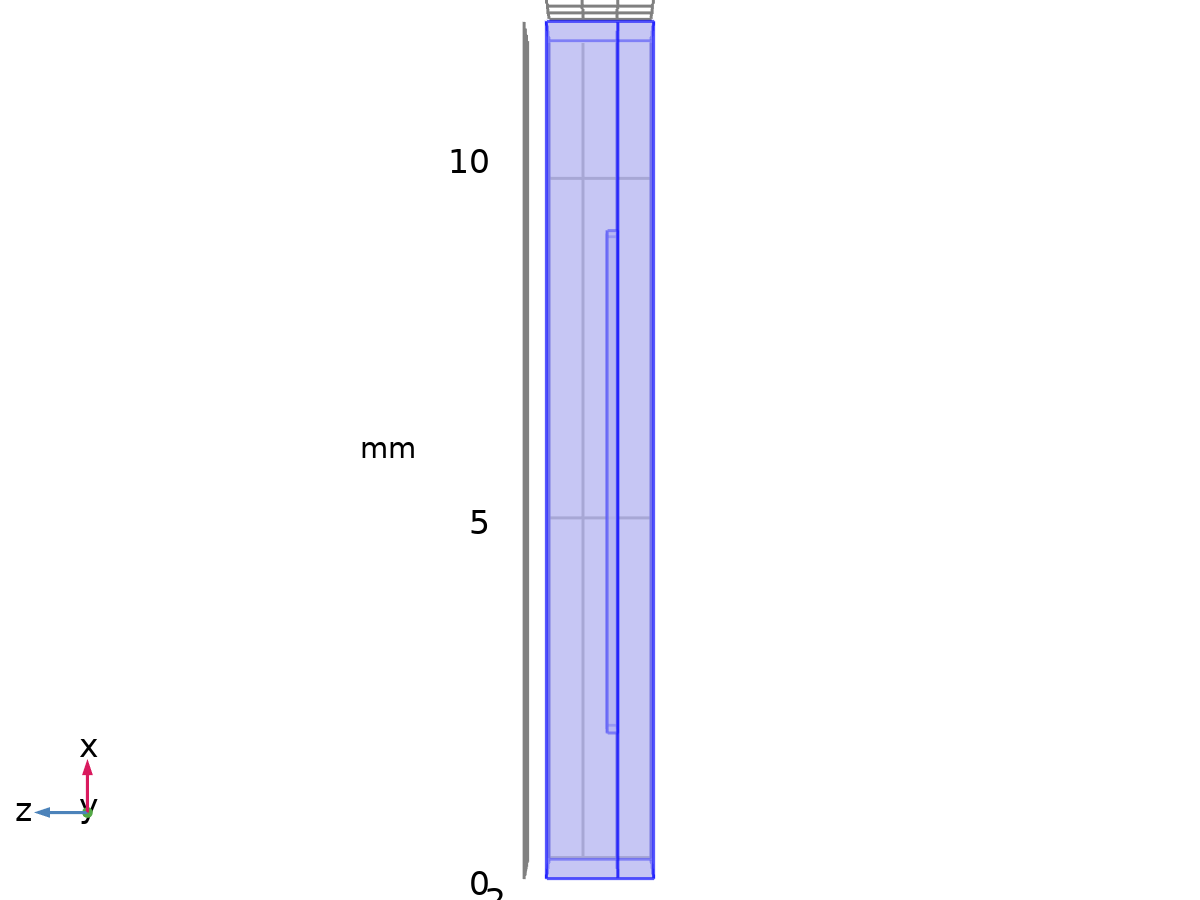


Initial Values 1

Selection

| Geometric entity level | Domain |
| --- | --- |
| Selection | Geometry geom1: Dimension 3: All domains |

#### Coordinate system selection

Settings

| **Description** | **Value** |
| --- | --- |
| Coordinate system | Global coordinate system |

Used products

| COMSOL Multiphysics |
| --- |

#### Variables

| **Name** | **Expression** | **Unit** | **Description** | **Selection** |
| --- | --- | --- | --- | --- |
| solid.uInitx | 0 | m | Initial value of displacement, x component | Domains 1–2 |
| solid.uInity | 0 | m | Initial value of displacement, y component | Domains 1–2 |
| solid.uInitz | 0 | m | Initial value of displacement, z component | Domains 1–2 |
| solid.utInitx | 0 | m/s | Initial value of structural velocity, x component | Domains 1–2 |
| solid.utInity | 0 | m/s | Initial value of structural velocity, y component | Domains 1–2 |
| solid.utInitz | 0 | m/s | Initial value of structural velocity, z component | Domains 1–2 |

- - 1. Hyperelastic Material 1


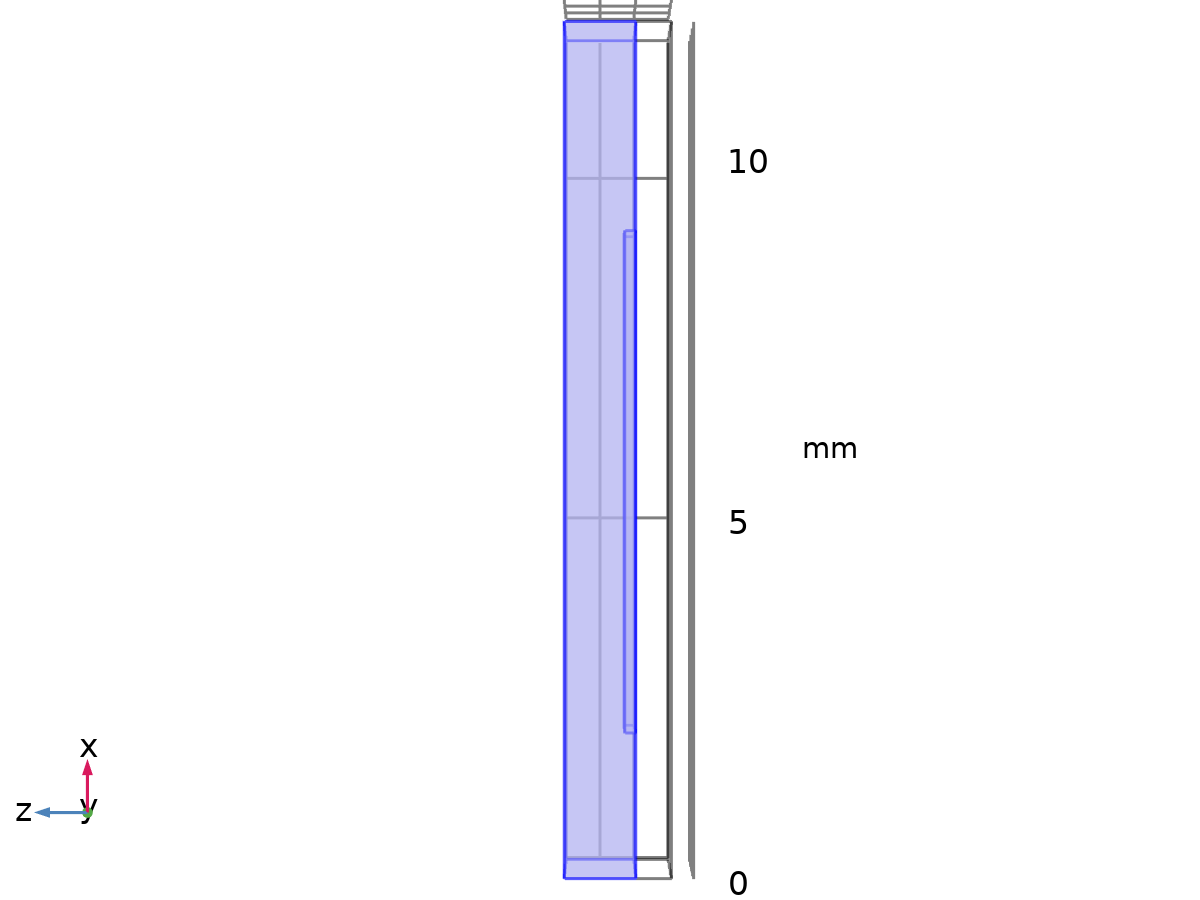


Hyperelastic Material 1

Selection

| Geometric entity level | Domain |
| --- | --- |
| Selection | Geometry geom1: Dimension 3: Domain 2 |

Equations


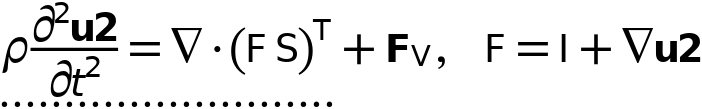


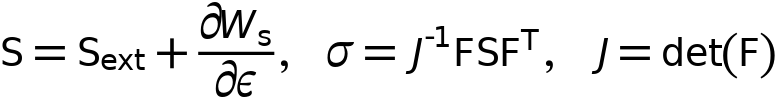


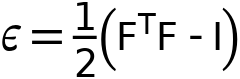


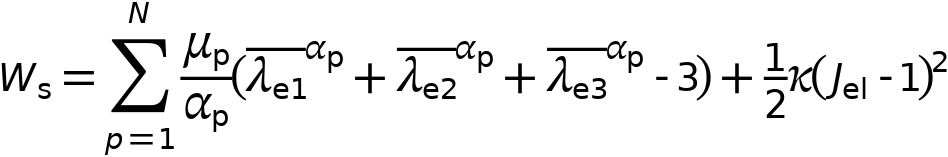


#### Hyperelastic material

Settings

| **Description** | **Value** |
| --- | --- |
| Material model | Ogden |
| Compressibility | Nearly incompressible material, quadratic volumetric strain energy |
| Bulk modulus | 9.62e8 |
| Density | From material |

| **p** | **Shear modulus** | **Alpha parameter** |
| --- | --- | --- |
| 1 | 1.11e-19 | 0 |
| 2 | 21660 | 3.268 |

#### Coordinate system selection

Settings

| **Description** | **Value** |
| --- | --- |
| Coordinate system | Global coordinate system |

#### Model input

Settings

| **Description** | **Value** |
| --- | --- |
| Volume reference temperature | User defined |
| Volume reference temperature | 293.15[K] |

Properties from material

| **Property** | **Material** | **Property group** |
| --- | --- | --- |
| Density | PDMS - Polydimethylsiloxane | Basic |

#### Variables

| **Name** | **Expression** | **Unit** | **Description** | **Selection** | **Details** |
| --- | --- | --- | --- | --- | --- |
| solid.u_tX | u2TIME | m/s | Velocity, X component | Domain 2 |  |
| solid.u_tY | v2TIME | m/s | Velocity, Y component | Domain 2 |  |
| solid.u_tZ | w2TIME | m/s | Velocity, Z component | Domain 2 |  |
| solid.Wk_tot | root.comp1.solid.hmm1.int9(solid.Wk) | J | Total kinetic energy | Global | + operation |
| solid.rho | material.rho | kg/m³ | Density | Domain 2 | Meta, * operation |
| u2TXt | u2TXTIME | 1/s | Tangential gradient of u2, X component, first time derivative | Boundaries 4–7, 9–11, 13–15, 17 |  |
| u2TYt | u2TYTIME | 1/s | Tangential gradient of u2, Y component, first time derivative | Boundaries 4–7, 9–11, 13–15, 17 |  |
| u2TZt | u2TZTIME | 1/s | Tangential gradient of u2, Z component, first time derivative | Boundaries 4–7, 9–11, 13–15, 17 |  |
| u2TXtt | u2TXTIMETIME | 1/s² | Tangential gradient of u2, X component, second time derivative | Boundaries 4–7, 9–11, 13–15, 17 |  |
| u2TYtt | u2TYTIMETIME | 1/s² | Tangential gradient of u2, Y component, second time derivative | Boundaries 4–7, 9–11, 13–15, 17 |  |
| u2TZtt | u2TZTIMETIME | 1/s² | Tangential gradient of u2, Z component, second time derivative | Boundaries 4–7, 9–11, 13–15, 17 |  |
| u2Xt | u2XTIME | 1/s | Gradient of u2, X component, first time derivative | Domain 2 |  |
| u2Yt | u2YTIME | 1/s | Gradient of u2, Y component, first time derivative | Domain 2 |  |
| u2Zt | u2ZTIME | 1/s | Gradient of u2, Z component, first time derivative | Domain 2 |  |
| u2Xtt | u2XTIMETIME | 1/s² | Gradient of u2, X component, second time derivative | Domain 2 |  |
| u2Ytt | u2YTIMETIME | 1/s² | Gradient of u2, Y component, second time derivative | Domain 2 |  |
| u2Ztt | u2ZTIMETIME | 1/s² | Gradient of u2, Z component, second time derivative | Domain 2 |  |
| v2TXt | v2TXTIME | 1/s | Tangential gradient of v2, X component, first time derivative | Boundaries 4–7, 9–11, 13–15, 17 |  |
| v2TYt | v2TYTIME | 1/s | Tangential gradient of v2, Y component, first time derivative | Boundaries 4–7, 9–11, 13–15, 17 |  |
| v2TZt | v2TZTIME | 1/s | Tangential gradient of v2, Z component, first time derivative | Boundaries 4–7, 9–11, 13–15, 17 |  |
| v2TXtt | v2TXTIMETIME | 1/s² | Tangential gradient of v2, X component, second time derivative | Boundaries 4–7, 9–11, 13–15, 17 |  |
| v2TYtt | v2TYTIMETIME | 1/s² | Tangential gradient of v2, Y component, second time derivative | Boundaries 4–7, 9–11, 13–15, 17 |  |
| v2TZtt | v2TZTIMETIME | 1/s² | Tangential gradient of v2, Z component, second time derivative | Boundaries 4–7, 9–11, 13–15, 17 |  |
| v2Xt | v2XTIME | 1/s | Gradient of v2, X component, first time derivative | Domain 2 |  |
| v2Yt | v2YTIME | 1/s | Gradient of v2, Y component, first time derivative | Domain 2 |  |
| v2Zt | v2ZTIME | 1/s | Gradient of v2, Z component, first time derivative | Domain 2 |  |
| v2Xtt | v2XTIMETIME | 1/s² | Gradient of v2, X component, second time derivative | Domain 2 |  |
| v2Ytt | v2YTIMETIME | 1/s² | Gradient of v2, Y component, second time derivative | Domain 2 |  |
| v2Ztt | v2ZTIMETIME | 1/s² | Gradient of v2, Z component, second time derivative | Domain 2 |  |
| w2TXt | w2TXTIME | 1/s | Tangential gradient of w2, X component, first time derivative | Boundaries 4–7, 9–11, 13–15, 17 |  |
| w2TYt | w2TYTIME | 1/s | Tangential gradient of w2, Y component, first time derivative | Boundaries 4–7, 9–11, 13–15, 17 |  |
| w2TZt | w2TZTIME | 1/s | Tangential gradient of w2, Z component, first time derivative | Boundaries 4–7, 9–11, 13–15, 17 |  |
| w2TXtt | w2TXTIMETIME | 1/s² | Tangential gradient of w2, X component, second time derivative | Boundaries 4–7, 9–11, 13–15, 17 |  |
| w2TYtt | w2TYTIMETIME | 1/s² | Tangential gradient of w2, Y component, second time derivative | Boundaries 4–7, 9–11, 13–15, 17 |  |
| w2TZtt | w2TZTIMETIME | 1/s² | Tangential gradient of w2, Z component, second time derivative | Boundaries 4–7, 9–11, 13–15, 17 |  |
| w2Xt | w2XTIME | 1/s | Gradient of w2, X component, first time derivative | Domain 2 |  |
| w2Yt | w2YTIME | 1/s | Gradient of w2, Y component, first time derivative | Domain 2 |  |
| w2Zt | w2ZTIME | 1/s | Gradient of w2, Z component, first time derivative | Domain 2 |  |
| w2Xtt | w2XTIMETIME | 1/s² | Gradient of w2, X component, second time derivative | Domain 2 |  |
| w2Ytt | w2YTIMETIME | 1/s² | Gradient of w2, Y component, second time derivative | Domain 2 |  |
| w2Ztt | w2ZTIMETIME | 1/s² | Gradient of w2, Z component, second time derivative | Domain 2 |  |
| u2t | u2TIME | m/s | Structural velocity field, X component | Domain 2 |  |
| v2t | v2TIME | m/s | Structural velocity field, Y component | Domain 2 |  |
| w2t | w2TIME | m/s | Structural velocity field, Z component | Domain 2 |  |
| u2tt | u2TIMETIME | m/s² | Acceleration field, X component | Domain 2 |  |
| v2tt | v2TIMETIME | m/s² | Acceleration field, Y component | Domain 2 |  |
| w2tt | w2TIMETIME | m/s² | Acceleration field, Z component | Domain 2 |  |
| solid.gradUxX | u2X | 1 | Displacement gradient, xX component | Domain 2 |  |
| solid.gradUyX | v2X | 1 | Displacement gradient, yX component | Domain 2 |  |
| solid.gradUzX | w2X | 1 | Displacement gradient, zX component | Domain 2 |  |
| solid.gradUxY | u2Y | 1 | Displacement gradient, xY component | Domain 2 |  |
| solid.gradUyY | v2Y | 1 | Displacement gradient, yY component | Domain 2 |  |
| solid.gradUzY | w2Y | 1 | Displacement gradient, zY component | Domain 2 |  |
| solid.gradUxZ | u2Z | 1 | Displacement gradient, xZ component | Domain 2 |  |
| solid.gradUyZ | v2Z | 1 | Displacement gradient, yZ component | Domain 2 |  |
| solid.gradUzZ | w2Z | 1 | Displacement gradient, zZ component | Domain 2 |  |
| solid.elogxx | log(solid.stchp1)*solid.LW11^2+log(solid.stchp2)*solid.LW12^2+log(solid.stchp3)*solid.LW13^2 | 1 | Logarithmic strain tensor, xx component | Domain 2 |  |
| solid.elogxy | log(solid.stchp1)*solid.LW11*solid.LW21+log(solid.stchp2)*solid.LW12*solid.LW22+log(solid.stchp3)*solid.LW13*solid.LW23 | 1 | Logarithmic strain tensor, xy component | Domain 2 |  |
| solid.elogxz | log(solid.stchp1)*solid.LW11*solid.LW31+log(solid.stchp2)*solid.LW12*solid.LW32+log(solid.stchp3)*solid.LW13*solid.LW33 | 1 | Logarithmic strain tensor, xz component | Domain 2 |  |
| solid.elogyy | log(solid.stchp1)*solid.LW21^2+log(solid.stchp2)*solid.LW22^2+log(solid.stchp3)*solid.LW23^2 | 1 | Logarithmic strain tensor, yy component | Domain 2 |  |
| solid.elogyz | log(solid.stchp1)*solid.LW21*solid.LW31+log(solid.stchp2)*solid.LW22*solid.LW32+log(solid.stchp3)*solid.LW23*solid.LW33 | 1 | Logarithmic strain tensor, yz component | Domain 2 |  |
| solid.elogzz | log(solid.stchp1)*solid.LW31^2+log(solid.stchp2)*solid.LW32^2+log(solid.stchp3)*solid.LW33^2 | 1 | Logarithmic strain tensor, zz component | Domain 2 |  |
| solid.FdxX | 1+solid.gradUxX | 1 | Deformation gradient, xX component | Domain 2 |  |
| solid.FdyX | solid.gradUyX | 1 | Deformation gradient, yX component | Domain 2 |  |
| solid.FdzX | solid.gradUzX | 1 | Deformation gradient, zX component | Domain 2 |  |
| solid.FdxY | solid.gradUxY | 1 | Deformation gradient, xY component | Domain 2 |  |
| solid.FdyY | 1+solid.gradUyY | 1 | Deformation gradient, yY component | Domain 2 |  |
| solid.FdzY | solid.gradUzY | 1 | Deformation gradient, zY component | Domain 2 |  |
| solid.FdxZ | solid.gradUxZ | 1 | Deformation gradient, xZ component | Domain 2 |  |
| solid.FdyZ | solid.gradUyZ | 1 | Deformation gradient, yZ component | Domain 2 |  |
| solid.FdzZ | 1+solid.gradUzZ | 1 | Deformation gradient, zZ component | Domain 2 |  |
| solid.Fdlx1 | solid.FdxX | 1 | Deformation gradient, local, x1 component | Domain 2 |  |
| solid.Fdly1 | solid.FdyX | 1 | Deformation gradient, local, y1 component | Domain 2 |  |
| solid.Fdlz1 | solid.FdzX | 1 | Deformation gradient, local, z1 component | Domain 2 |  |
| solid.Fdlx2 | solid.FdxY | 1 | Deformation gradient, local, x2 component | Domain 2 |  |
| solid.Fdly2 | solid.FdyY | 1 | Deformation gradient, local, y2 component | Domain 2 |  |
| solid.Fdlz2 | solid.FdzY | 1 | Deformation gradient, local, z2 component | Domain 2 |  |
| solid.Fdlx3 | solid.FdxZ | 1 | Deformation gradient, local, x3 component | Domain 2 |  |
| solid.Fdly3 | solid.FdyZ | 1 | Deformation gradient, local, y3 component | Domain 2 |  |
| solid.Fdlz3 | solid.FdzZ | 1 | Deformation gradient, local, z3 component | Domain 2 |  |
| solid.K | -d(solid.pm,solid.J) | N/m² | Bulk modulus | Domain 2 | * operation |
| solid.Eequ | 4.5*solid.kappa*(solid.mup1*solid.alphap1+solid.mup2*solid.alphap2)/(3*solid.kappa+0.5*solid.mup1*solid.alphap1+0.5*solid.mup2*solid.alphap2) | Pa | Equivalent Young's modulus | Domain 2 |  |
| solid.nuequ | (3*solid.kappa-solid.mup1*solid.alphap1-solid.mup2*solid.alphap2)/(6*solid.kappa+solid.mup1*solid.alphap1+solid.mup2*solid.alphap2) | 1 | Equivalent Poisson's ratio | Domain 2 |  |
| solid.cp | sqrt((solid.kappa+2*(solid.mup1*solid.alphap1+solid.mup2*solid.alphap2)/3)/solid.rho) | m/s | Equivalent speed of pressure wave | Domain 2 |  |
| solid.cs | sqrt(0.5*(solid.mup1*solid.alphap1+solid.mup2*solid.alphap2)/solid.rho) | m/s | Equivalent speed of shear wave | Domain 2 |  |
| solid.eXX | solid.el11 | 1 | Strain tensor, XX component | Domain 2 | + operation |
| solid.eXY | solid.el12 | 1 | Strain tensor, XY component | Domain 2 | + operation |
| solid.eXZ | solid.el13 | 1 | Strain tensor, XZ component | Domain 2 | + operation |
| solid.eYY | solid.el22 | 1 | Strain tensor, YY component | Domain 2 | + operation |
| solid.eYZ | solid.el23 | 1 | Strain tensor, YZ component | Domain 2 | + operation |
| solid.eZZ | solid.el33 | 1 | Strain tensor, ZZ component | Domain 2 | + operation |
| solid.el11 | 0.5*(-1+solid.Cl11) | 1 | Strain tensor, local coordinate system, 11 component | Domain 2 | + operation |
| solid.el12 | 0.5*solid.Cl12 | 1 | Strain tensor, local coordinate system, 12 component | Domain 2 | + operation |
| solid.el13 | 0.5*solid.Cl13 | 1 | Strain tensor, local coordinate system, 13 component | Domain 2 | + operation |
| solid.el22 | 0.5*(-1+solid.Cl22) | 1 | Strain tensor, local coordinate system, 22 component | Domain 2 | + operation |
| solid.el23 | 0.5*solid.Cl23 | 1 | Strain tensor, local coordinate system, 23 component | Domain 2 | + operation |
| solid.el33 | 0.5*(-1+solid.Cl33) | 1 | Strain tensor, local coordinate system, 33 component | Domain 2 | + operation |
| solid.J | sqrt(solid.Cl11*solid.Cl22*solid.Cl33+2*solid.Cl12*solid.Cl23*solid.Cl13-solid.Cl11*solid.Cl23^2-solid.Cl12^2*solid.Cl33-solid.Cl22*solid.Cl13^2) | 1 | Volume ratio | Domain 2 |  |
| solid.eX | solid.eXX | 1 | Strain tensor, X component | Domain 2 |  |
| solid.eY | solid.eYY | 1 | Strain tensor, Y component | Domain 2 |  |
| solid.eZ | solid.eZZ | 1 | Strain tensor, Z component | Domain 2 |  |
| solid.Cl11 | solid.Fdlx1^2+solid.Fdly1^2+solid.Fdlz1^2 | 1 | Cauchy-Green tensor, local coordinate system, 11 component | Domain 2 |  |
| solid.Cl12 | solid.Fdlx1*solid.Fdlx2+solid.Fdly1*solid.Fdly2+solid.Fdlz1*solid.Fdlz2 | 1 | Cauchy-Green tensor, local coordinate system, 12 component | Domain 2 |  |
| solid.Cl13 | solid.Fdlx1*solid.Fdlx3+solid.Fdly1*solid.Fdly3+solid.Fdlz1*solid.Fdlz3 | 1 | Cauchy-Green tensor, local coordinate system, 13 component | Domain 2 |  |
| solid.Cl22 | solid.Fdlx2^2+solid.Fdly2^2+solid.Fdlz2^2 | 1 | Cauchy-Green tensor, local coordinate system, 22 component | Domain 2 |  |
| solid.Cl23 | solid.Fdlx2*solid.Fdlx3+solid.Fdly2*solid.Fdly3+solid.Fdlz2*solid.Fdlz3 | 1 | Cauchy-Green tensor, local coordinate system, 23 component | Domain 2 |  |
| solid.Cl33 | solid.Fdlx3^2+solid.Fdly3^2+solid.Fdlz3^2 | 1 | Cauchy-Green tensor, local coordinate system, 33 component | Domain 2 |  |
| solid.eel11 | 0.5*(-1+solid.Cel11) | 1 | Elastic strain tensor, local coordinate system, 11 component | Domain 2 |  |
| solid.eel12 | 0.5*solid.Cel12 | 1 | Elastic strain tensor, local coordinate system, 12 component | Domain 2 |  |
| solid.eel13 | 0.5*solid.Cel13 | 1 | Elastic strain tensor, local coordinate system, 13 component | Domain 2 |  |
| solid.eel22 | 0.5*(-1+solid.Cel22) | 1 | Elastic strain tensor, local coordinate system, 22 component | Domain 2 |  |
| solid.eel23 | 0.5*solid.Cel23 | 1 | Elastic strain tensor, local coordinate system, 23 component | Domain 2 |  |
| solid.eel33 | 0.5*(-1+solid.Cel33) | 1 | Elastic strain tensor, local coordinate system, 33 component | Domain 2 |  |
| solid.Felx1 | solid.Fdlx1*solid.Fiil11+solid.Fdlx2*solid.Fiil21+solid.Fdlx3*solid.Fiil31 | 1 | Elastic deformation gradient, local coordinate system, x1 component | Domain 2 |  |
| solid.Fely1 | solid.Fdly1*solid.Fiil11+solid.Fdly2*solid.Fiil21+solid.Fdly3*solid.Fiil31 | 1 | Elastic deformation gradient, local coordinate system, y1 component | Domain 2 |  |
| solid.Felz1 | solid.Fdlz1*solid.Fiil11+solid.Fdlz2*solid.Fiil21+solid.Fdlz3*solid.Fiil31 | 1 | Elastic deformation gradient, local coordinate system, z1 component | Domain 2 |  |
| solid.Felx2 | solid.Fdlx1*solid.Fiil12+solid.Fdlx2*solid.Fiil22+solid.Fdlx3*solid.Fiil32 | 1 | Elastic deformation gradient, local coordinate system, x2 component | Domain 2 |  |
| solid.Fely2 | solid.Fdly1*solid.Fiil12+solid.Fdly2*solid.Fiil22+solid.Fdly3*solid.Fiil32 | 1 | Elastic deformation gradient, local coordinate system, y2 component | Domain 2 |  |
| solid.Felz2 | solid.Fdlz1*solid.Fiil12+solid.Fdlz2*solid.Fiil22+solid.Fdlz3*solid.Fiil32 | 1 | Elastic deformation gradient, local coordinate system, z2 component | Domain 2 |  |
| solid.Felx3 | solid.Fdlx1*solid.Fiil13+solid.Fdlx2*solid.Fiil23+solid.Fdlx3*solid.Fiil33 | 1 | Elastic deformation gradient, local coordinate system, x3 component | Domain 2 |  |
| solid.Fely3 | solid.Fdly1*solid.Fiil13+solid.Fdly2*solid.Fiil23+solid.Fdly3*solid.Fiil33 | 1 | Elastic deformation gradient, local coordinate system, y3 component | Domain 2 |  |
| solid.Felz3 | solid.Fdlz1*solid.Fiil13+solid.Fdlz2*solid.Fiil23+solid.Fdlz3*solid.Fiil33 | 1 | Elastic deformation gradient, local coordinate system, z3 component | Domain 2 |  |
| solid.Cel11 | (solid.Fiil11*solid.Cl11+solid.Fiil21*solid.Cl12+solid.Fiil31*solid.Cl13)*solid.Fiil11+(solid.Fiil11*solid.Cl12+solid.Fiil21*solid.Cl22+solid.Fiil31*solid.Cl23)*solid.Fiil21+(solid.Fiil11*solid.Cl13+solid.Fiil21*solid.Cl23+solid.Fiil31*solid.Cl33)*solid.Fiil31 | 1 | Elastic Cauchy-Green tensor, local coordinate system, 11 component | Domain 2 |  |
| solid.Cel12 | (solid.Fiil11*solid.Cl11+solid.Fiil21*solid.Cl12+solid.Fiil31*solid.Cl13)*solid.Fiil12+(solid.Fiil11*solid.Cl12+solid.Fiil21*solid.Cl22+solid.Fiil31*solid.Cl23)*solid.Fiil22+(solid.Fiil11*solid.Cl13+solid.Fiil21*solid.Cl23+solid.Fiil31*solid.Cl33)*solid.Fiil32 | 1 | Elastic Cauchy-Green tensor, local coordinate system, 12 component | Domain 2 |  |
| solid.Cel13 | (solid.Fiil11*solid.Cl11+solid.Fiil21*solid.Cl12+solid.Fiil31*solid.Cl13)*solid.Fiil13+(solid.Fiil11*solid.Cl12+solid.Fiil21*solid.Cl22+solid.Fiil31*solid.Cl23)*solid.Fiil23+(solid.Fiil11*solid.Cl13+solid.Fiil21*solid.Cl23+solid.Fiil31*solid.Cl33)*solid.Fiil33 | 1 | Elastic Cauchy-Green tensor, local coordinate system, 13 component | Domain 2 |  |
| solid.Cel22 | (solid.Fiil12*solid.Cl11+solid.Fiil22*solid.Cl12+solid.Fiil32*solid.Cl13)*solid.Fiil12+(solid.Fiil12*solid.Cl12+solid.Fiil22*solid.Cl22+solid.Fiil32*solid.Cl23)*solid.Fiil22+(solid.Fiil12*solid.Cl13+solid.Fiil22*solid.Cl23+solid.Fiil32*solid.Cl33)*solid.Fiil32 | 1 | Elastic Cauchy-Green tensor, local coordinate system, 22 component | Domain 2 |  |
| solid.Cel23 | (solid.Fiil12*solid.Cl11+solid.Fiil22*solid.Cl12+solid.Fiil32*solid.Cl13)*solid.Fiil13+(solid.Fiil12*solid.Cl12+solid.Fiil22*solid.Cl22+solid.Fiil32*solid.Cl23)*solid.Fiil23+(solid.Fiil12*solid.Cl13+solid.Fiil22*solid.Cl23+solid.Fiil32*solid.Cl33)*solid.Fiil33 | 1 | Elastic Cauchy-Green tensor, local coordinate system, 23 component | Domain 2 |  |
| solid.Cel33 | (solid.Fiil13*solid.Cl11+solid.Fiil23*solid.Cl12+solid.Fiil33*solid.Cl13)*solid.Fiil13+(solid.Fiil13*solid.Cl12+solid.Fiil23*solid.Cl22+solid.Fiil33*solid.Cl23)*solid.Fiil23+(solid.Fiil13*solid.Cl13+solid.Fiil23*solid.Cl23+solid.Fiil33*solid.Cl33)*solid.Fiil33 | 1 | Elastic Cauchy-Green tensor, local coordinate system, 33 component | Domain 2 |  |
| solid.Fiil11 | 1 | 1 | Inelastic deformation gradient inverse, local coordinate system, 11 component | Domain 2 | Matrix multiplication |
| solid.Fiil21 | 0 | 1 | Inelastic deformation gradient inverse, local coordinate system, 21 component | Domain 2 | Matrix multiplication |
| solid.Fiil31 | 0 | 1 | Inelastic deformation gradient inverse, local coordinate system, 31 component | Domain 2 | Matrix multiplication |
| solid.Fiil12 | 0 | 1 | Inelastic deformation gradient inverse, local coordinate system, 12 component | Domain 2 | Matrix multiplication |
| solid.Fiil22 | 1 | 1 | Inelastic deformation gradient inverse, local coordinate system, 22 component | Domain 2 | Matrix multiplication |
| solid.Fiil32 | 0 | 1 | Inelastic deformation gradient inverse, local coordinate system, 32 component | Domain 2 | Matrix multiplication |
| solid.Fiil13 | 0 | 1 | Inelastic deformation gradient inverse, local coordinate system, 13 component | Domain 2 | Matrix multiplication |
| solid.Fiil23 | 0 | 1 | Inelastic deformation gradient inverse, local coordinate system, 23 component | Domain 2 | Matrix multiplication |
| solid.Fiil33 | 1 | 1 | Inelastic deformation gradient inverse, local coordinate system, 33 component | Domain 2 | Matrix multiplication |
| solid.Ji | 1/(solid.Fiil11*solid.Fiil22*solid.Fiil33+solid.Fiil12*solid.Fiil23*solid.Fiil31+solid.Fiil13*solid.Fiil21*solid.Fiil32-solid.Fiil11*solid.Fiil23*solid.Fiil32-solid.Fiil12*solid.Fiil21*solid.Fiil33-solid.Fiil13*solid.Fiil22*solid.Fiil31) | 1 | Inelastic volume ratio | Domain 2 |  |
| solid.Ceil11 | (solid.Cel22*solid.Cel33-solid.Cel23^2)/(solid.Cel11*solid.Cel22*solid.Cel33+2*solid.Cel12*solid.Cel23*solid.Cel13-solid.Cel11*solid.Cel23^2-solid.Cel12^2*solid.Cel33-solid.Cel22*solid.Cel13^2) | 1 | Elastic Cauchy-Green tensor inverse, local coordinate system, 11 component | Domain 2 |  |
| solid.Ceil12 | (solid.Cel23*solid.Cel13-solid.Cel12*solid.Cel33)/(solid.Cel11*solid.Cel22*solid.Cel33+2*solid.Cel12*solid.Cel23*solid.Cel13-solid.Cel11*solid.Cel23^2-solid.Cel12^2*solid.Cel33-solid.Cel22*solid.Cel13^2) | 1 | Elastic Cauchy-Green tensor inverse, local coordinate system, 12 component | Domain 2 |  |
| solid.Ceil13 | (solid.Cel12*solid.Cel23-solid.Cel22*solid.Cel13)/(solid.Cel11*solid.Cel22*solid.Cel33+2*solid.Cel12*solid.Cel23*solid.Cel13-solid.Cel11*solid.Cel23^2-solid.Cel12^2*solid.Cel33-solid.Cel22*solid.Cel13^2) | 1 | Elastic Cauchy-Green tensor inverse, local coordinate system, 13 component | Domain 2 |  |
| solid.Ceil22 | (solid.Cel11*solid.Cel33-solid.Cel13^2)/(solid.Cel11*solid.Cel22*solid.Cel33+2*solid.Cel12*solid.Cel23*solid.Cel13-solid.Cel11*solid.Cel23^2-solid.Cel12^2*solid.Cel33-solid.Cel22*solid.Cel13^2) | 1 | Elastic Cauchy-Green tensor inverse, local coordinate system, 22 component | Domain 2 |  |
| solid.Ceil23 | (solid.Cel12*solid.Cel13-solid.Cel11*solid.Cel23)/(solid.Cel11*solid.Cel22*solid.Cel33+2*solid.Cel12*solid.Cel23*solid.Cel13-solid.Cel11*solid.Cel23^2-solid.Cel12^2*solid.Cel33-solid.Cel22*solid.Cel13^2) | 1 | Elastic Cauchy-Green tensor inverse, local coordinate system, 23 component | Domain 2 |  |
| solid.Ceil33 | (solid.Cel11*solid.Cel22-solid.Cel12^2)/(solid.Cel11*solid.Cel22*solid.Cel33+2*solid.Cel12*solid.Cel23*solid.Cel13-solid.Cel11*solid.Cel23^2-solid.Cel12^2*solid.Cel33-solid.Cel22*solid.Cel13^2) | 1 | Elastic Cauchy-Green tensor inverse, local coordinate system, 33 component | Domain 2 |  |
| solid.Jel | solid.J/solid.Ji | 1 | Elastic volume ratio | Domain 2 |  |
| solid.mises | (3*solid.II2s)^0.5 | N/m² | von Mises stress | Domain 2 |  |
| solid.Sl11 | solid.Sliso11+solid.Slvol11 | N/m² | Second Piola-Kirchhoff stress, local coordinate system, 11 component | Domain 2 | + operation |
| solid.Sl12 | solid.Sliso12+solid.Slvol12 | N/m² | Second Piola-Kirchhoff stress, local coordinate system, 12 component | Domain 2 | + operation |
| solid.Sl13 | solid.Sliso13+solid.Slvol13 | N/m² | Second Piola-Kirchhoff stress, local coordinate system, 13 component | Domain 2 | + operation |
| solid.Sl22 | solid.Sliso22+solid.Slvol22 | N/m² | Second Piola-Kirchhoff stress, local coordinate system, 22 component | Domain 2 | + operation |
| solid.Sl23 | solid.Sliso23+solid.Slvol23 | N/m² | Second Piola-Kirchhoff stress, local coordinate system, 23 component | Domain 2 | + operation |
| solid.Sl33 | solid.Sliso33+solid.Slvol33 | N/m² | Second Piola-Kirchhoff stress, local coordinate system, 33 component | Domain 2 | + operation |
| solid.SXX | solid.Sl11 | N/m² | Second Piola-Kirchhoff stress, XX component | Domain 2 |  |
| solid.SXY | solid.Sl12 | N/m² | Second Piola-Kirchhoff stress, XY component | Domain 2 |  |
| solid.SXZ | solid.Sl13 | N/m² | Second Piola-Kirchhoff stress, XZ component | Domain 2 |  |
| solid.SYY | solid.Sl22 | N/m² | Second Piola-Kirchhoff stress, YY component | Domain 2 |  |
| solid.SYZ | solid.Sl23 | N/m² | Second Piola-Kirchhoff stress, YZ component | Domain 2 |  |
| solid.SZZ | solid.Sl33 | N/m² | Second Piola-Kirchhoff stress, ZZ component | Domain 2 |  |
| solid.u_ttX | d(u2TIME,TIME) | m/s² | Acceleration, X component | Domain 2 |  |
| solid.u_ttY | d(v2TIME,TIME) | m/s² | Acceleration, Y component | Domain 2 |  |
| solid.u_ttZ | d(w2TIME,TIME) | m/s² | Acceleration, Z component | Domain 2 |  |
| solid.afX | 0 | m/s² | Frame acceleration, X component | Domain 2 | + operation |
| solid.afY | 0 | m/s² | Frame acceleration, Y component | Domain 2 | + operation |
| solid.afZ | 0 | m/s² | Frame acceleration, Z component | Domain 2 | + operation |
| solid.accX | solid.u_ttX | m/s² | Total acceleration, X component | Domain 2 | + operation |
| solid.accY | solid.u_ttY | m/s² | Total acceleration, Y component | Domain 2 | + operation |
| solid.accZ | solid.u_ttZ | m/s² | Total acceleration, Z component | Domain 2 | + operation |
| solid.vel | sqrt(real(solid.u_tX)^2+real(solid.u_tY)^2+real(solid.u_tZ)^2) | m/s | Velocity magnitude | Domain 2 |  |
| solid.acc | sqrt(real(solid.accX)^2+real(solid.accY)^2+real(solid.accZ)^2) | m/s² | Total acceleration magnitude | Domain 2 |  |
| solid.u_tt | sqrt(real(solid.u_ttX)^2+real(solid.u_ttY)^2+real(solid.u_ttZ)^2) | m/s² | Acceleration magnitude | Domain 2 |  |
| solid.disp | sqrt(real(u2)^2+real(v2)^2+real(w2)^2) | m | Total displacement | Domain 2 |  |
| solid.sl11 | solid.sx | N/m² | Stress tensor, local coordinate system, 11 component | Domain 2 |  |
| solid.sl12 | solid.sxy | N/m² | Stress tensor, local coordinate system, 12 component | Domain 2 |  |
| solid.sl13 | solid.sxz | N/m² | Stress tensor, local coordinate system, 13 component | Domain 2 |  |
| solid.sl22 | solid.sy | N/m² | Stress tensor, local coordinate system, 22 component | Domain 2 |  |
| solid.sl23 | solid.syz | N/m² | Stress tensor, local coordinate system, 23 component | Domain 2 |  |
| solid.sl33 | solid.sz | N/m² | Stress tensor, local coordinate system, 33 component | Domain 2 |  |
| solid.eelvol | solid.eel11+solid.eel22+solid.eel33 | 1 | First invariant of elastic strain tensor | Domain 2 |  |
| solid.eeldev11 | solid.eel11-(solid.eel11+solid.eel22+solid.eel33)/3 | 1 | Deviatoric elastic strain tensor, local coordinate system, 11 component | Domain 2 |  |
| solid.eeldev12 | solid.eel12 | 1 | Deviatoric elastic strain tensor, local coordinate system, 12 component | Domain 2 |  |
| solid.eeldev13 | solid.eel13 | 1 | Deviatoric elastic strain tensor, local coordinate system, 13 component | Domain 2 |  |
| solid.eeldev22 | solid.eel22-(solid.eel11+solid.eel22+solid.eel33)/3 | 1 | Deviatoric elastic strain tensor, local coordinate system, 22 component | Domain 2 |  |
| solid.eeldev23 | solid.eel23 | 1 | Deviatoric elastic strain tensor, local coordinate system, 23 component | Domain 2 |  |
| solid.eeldev33 | solid.eel33-(solid.eel11+solid.eel22+solid.eel33)/3 | 1 | Deviatoric elastic strain tensor, local coordinate system, 33 component | Domain 2 |  |
| solid.II2eel | 0.5*((solid.eel11-(solid.eel11+solid.eel22+solid.eel33)/3)^2+2*solid.eel12^2+2*solid.eel13^2+(solid.eel22-(solid.eel11+solid.eel22+solid.eel33)/3)^2+2*solid.eel23^2+(solid.eel33-(solid.eel11+solid.eel22+solid.eel33)/3)^2) | 1 | Second invariant of deviatoric elastic strain tensor | Domain 2 |  |
| solid.Qh | 0 | W/m³ | Total power dissipation density | Domain 2 | + operation |
| solid.pwTXt | solid.pwTXTIME | kg/(m²·s³) | Tangential gradient of pw, X component, first time derivative | Boundaries 4–7, 9–11, 13–15, 17 |  |
| solid.pwTYt | solid.pwTYTIME | kg/(m²·s³) | Tangential gradient of pw, Y component, first time derivative | Boundaries 4–7, 9–11, 13–15, 17 |  |
| solid.pwTZt | solid.pwTZTIME | kg/(m²·s³) | Tangential gradient of pw, Z component, first time derivative | Boundaries 4–7, 9–11, 13–15, 17 |  |
| solid.pwTXtt | solid.pwTXTIMETIME | kg/(m²·s⁴) | Tangential gradient of pw, X component, second time derivative | Boundaries 4–7, 9–11, 13–15, 17 |  |
| solid.pwTYtt | solid.pwTYTIMETIME | kg/(m²·s⁴) | Tangential gradient of pw, Y component, second time derivative | Boundaries 4–7, 9–11, 13–15, 17 |  |
| solid.pwTZtt | solid.pwTZTIMETIME | kg/(m²·s⁴) | Tangential gradient of pw, Z component, second time derivative | Boundaries 4–7, 9–11, 13–15, 17 |  |
| solid.pwXt | solid.pwXTIME | kg/(m²·s³) | Gradient of pw, X component, first time derivative | Domain 2 |  |
| solid.pwYt | solid.pwYTIME | kg/(m²·s³) | Gradient of pw, Y component, first time derivative | Domain 2 |  |
| solid.pwZt | solid.pwZTIME | kg/(m²·s³) | Gradient of pw, Z component, first time derivative | Domain 2 |  |
| solid.pwXtt | solid.pwXTIMETIME | kg/(m²·s⁴) | Gradient of pw, X component, second time derivative | Domain 2 |  |
| solid.pwYtt | solid.pwYTIMETIME | kg/(m²·s⁴) | Gradient of pw, Y component, second time derivative | Domain 2 |  |
| solid.pwZtt | solid.pwZTIMETIME | kg/(m²·s⁴) | Gradient of pw, Z component, second time derivative | Domain 2 |  |
| solid.pwt | solid.pwTIME | W/m³ | Auxiliary pressure, first time derivative | Domain 2 |  |
| solid.pwt | solid.pwTIME | W/m³ | Auxiliary pressure, first time derivative | Boundaries 4–7, 9–11, 13–15, 17 |  |
| solid.pwtt | solid.pwTIMETIME | Pa/s² | Auxiliary pressure, second time derivative | Domain 2 |  |
| solid.pwtt | solid.pwTIMETIME | Pa/s² | Auxiliary pressure, second time derivative | Boundaries 4–7, 9–11, 13–15, 17 |  |
| solid.sysT11 | 1 | 1 | Transform to global system, 11 component | Domain 2 |  |
| solid.sysT21 | 0 | 1 | Transform to global system, 21 component | Domain 2 |  |
| solid.sysT31 | 0 | 1 | Transform to global system, 31 component | Domain 2 |  |
| solid.sysT12 | 0 | 1 | Transform to global system, 12 component | Domain 2 |  |
| solid.sysT22 | 1 | 1 | Transform to global system, 22 component | Domain 2 |  |
| solid.sysT32 | 0 | 1 | Transform to global system, 32 component | Domain 2 |  |
| solid.sysT13 | 0 | 1 | Transform to global system, 13 component | Domain 2 |  |
| solid.sysT23 | 0 | 1 | Transform to global system, 23 component | Domain 2 |  |
| solid.sysT33 | 1 | 1 | Transform to global system, 33 component | Domain 2 |  |
| solid.kappa | 962000000 | Pa | Bulk modulus | Domain 2 |  |
| solid.Ws | solid.Wsvol+solid.Wsiso | J/m³ | Elastic strain energy density | Domain 2 | * operation |
| solid.Wsiso | solid.Ji*(solid.mup1*(-3+solid.stchIelp1^solid.alphap1+solid.stchIelp2^solid.alphap1+solid.stchIelp3^solid.alphap1)/solid.alphap1+solid.mup2*(-3+solid.stchIelp1^solid.alphap2+solid.stchIelp2^solid.alphap2+solid.stchIelp3^solid.alphap2)/solid.alphap2) | J/m³ | Isochoric strain energy density | Domain 2 | * operation |
| solid.Wsvol | -solid.pw*solid.Ji*(-1+solid.Jel) | J/m³ | Volumetric strain energy density | Domain 2 | * operation |
| solid.Uvol | 0.5*solid.Ji*solid.kappa*(-1+solid.Jel)^2 | J/m³ | Auxiliary volumetric energy density | Domain 2 |  |
| solid.Sliso11 | 2*d(solid.Wsiso,solid.Cl11) | N/m² | Second Piola-Kirchhoff isochoric stress local coordinate system, 11 component | Domain 2 | + operation |
| solid.Sliso12 | d(solid.Wsiso,solid.Cl12) | N/m² | Second Piola-Kirchhoff isochoric stress local coordinate system, 12 component | Domain 2 | + operation |
| solid.Sliso13 | d(solid.Wsiso,solid.Cl13) | N/m² | Second Piola-Kirchhoff isochoric stress local coordinate system, 13 component | Domain 2 | + operation |
| solid.Sliso22 | 2*d(solid.Wsiso,solid.Cl22) | N/m² | Second Piola-Kirchhoff isochoric stress local coordinate system, 22 component | Domain 2 | + operation |
| solid.Sliso23 | d(solid.Wsiso,solid.Cl23) | N/m² | Second Piola-Kirchhoff isochoric stress local coordinate system, 23 component | Domain 2 | + operation |
| solid.Sliso33 | 2*d(solid.Wsiso,solid.Cl33) | N/m² | Second Piola-Kirchhoff isochoric stress local coordinate system, 33 component | Domain 2 | + operation |
| solid.Slvol11 | -solid.pw*solid.J*(solid.Cl22*solid.Cl33-solid.Cl23^2)/(solid.Cl11*solid.Cl22*solid.Cl33+2*solid.Cl12*solid.Cl23*solid.Cl13-solid.Cl11*solid.Cl23^2-solid.Cl12^2*solid.Cl33-solid.Cl22*solid.Cl13^2) | N/m² | Second Piola-Kirchhoff volumetric stress local coordinate system, 11 component | Domain 2 | * operation |
| solid.Slvol12 | -solid.pw*solid.J*(solid.Cl23*solid.Cl13-solid.Cl12*solid.Cl33)/(solid.Cl11*solid.Cl22*solid.Cl33+2*solid.Cl12*solid.Cl23*solid.Cl13-solid.Cl11*solid.Cl23^2-solid.Cl12^2*solid.Cl33-solid.Cl22*solid.Cl13^2) | N/m² | Second Piola-Kirchhoff volumetric stress local coordinate system, 12 component | Domain 2 | * operation |
| solid.Slvol13 | -solid.pw*solid.J*(solid.Cl12*solid.Cl23-solid.Cl22*solid.Cl13)/(solid.Cl11*solid.Cl22*solid.Cl33+2*solid.Cl12*solid.Cl23*solid.Cl13-solid.Cl11*solid.Cl23^2-solid.Cl12^2*solid.Cl33-solid.Cl22*solid.Cl13^2) | N/m² | Second Piola-Kirchhoff volumetric stress local coordinate system, 13 component | Domain 2 | * operation |
| solid.Slvol22 | -solid.pw*solid.J*(solid.Cl11*solid.Cl33-solid.Cl13^2)/(solid.Cl11*solid.Cl22*solid.Cl33+2*solid.Cl12*solid.Cl23*solid.Cl13-solid.Cl11*solid.Cl23^2-solid.Cl12^2*solid.Cl33-solid.Cl22*solid.Cl13^2) | N/m² | Second Piola-Kirchhoff volumetric stress local coordinate system, 22 component | Domain 2 | * operation |
| solid.Slvol23 | -solid.pw*solid.J*(solid.Cl12*solid.Cl13-solid.Cl11*solid.Cl23)/(solid.Cl11*solid.Cl22*solid.Cl33+2*solid.Cl12*solid.Cl23*solid.Cl13-solid.Cl11*solid.Cl23^2-solid.Cl12^2*solid.Cl33-solid.Cl22*solid.Cl13^2) | N/m² | Second Piola-Kirchhoff volumetric stress local coordinate system, 23 component | Domain 2 | * operation |
| solid.Slvol33 | -solid.pw*solid.J*(solid.Cl11*solid.Cl22-solid.Cl12^2)/(solid.Cl11*solid.Cl22*solid.Cl33+2*solid.Cl12*solid.Cl23*solid.Cl13-solid.Cl11*solid.Cl23^2-solid.Cl12^2*solid.Cl33-solid.Cl22*solid.Cl13^2) | N/m² | Second Piola-Kirchhoff volumetric stress local coordinate system, 33 component | Domain 2 | * operation |
| solid.pm | -d(solid.Uvol,solid.J) | N/m² | Pressure | Domain 2 |  |
| solid.Wh | solid.Ws | J/m³ | Stored energy density | Domain 2 | + operation |
| solid.I1Cel | solid.Cel11+solid.Cel22+solid.Cel33 | 1 | First invariant of elastic right Cauchy-Green tensor | Domain 2 |  |
| solid.I2Cel | 0.5*(solid.I1Cel^2-solid.Cel11^2-2*solid.Cel12^2-2*solid.Cel13^2-solid.Cel22^2-2*solid.Cel23^2-solid.Cel33^2) | 1 | Second invariant of elastic right Cauchy-Green tensor | Domain 2 |  |
| solid.I3Cel | solid.Cel11*solid.Cel22*solid.Cel33+2*solid.Cel12*solid.Cel23*solid.Cel13-solid.Cel11*solid.Cel23^2-solid.Cel12^2*solid.Cel33-solid.Cel22*solid.Cel13^2 | 1 | Third invariant of elastic right Cauchy-Green tensor | Domain 2 |  |
| solid.CIel11 | solid.Jel^(-2/3)*solid.Cel11 | 1 | Isochoric elastic Cauchy-Green tensor, local coordinate system, 11 component | Domain 2 |  |
| solid.CIel12 | solid.Jel^(-2/3)*solid.Cel12 | 1 | Isochoric elastic Cauchy-Green tensor, local coordinate system, 12 component | Domain 2 |  |
| solid.CIel13 | solid.Jel^(-2/3)*solid.Cel13 | 1 | Isochoric elastic Cauchy-Green tensor, local coordinate system, 13 component | Domain 2 |  |
| solid.CIel22 | solid.Jel^(-2/3)*solid.Cel22 | 1 | Isochoric elastic Cauchy-Green tensor, local coordinate system, 22 component | Domain 2 |  |
| solid.CIel23 | solid.Jel^(-2/3)*solid.Cel23 | 1 | Isochoric elastic Cauchy-Green tensor, local coordinate system, 23 component | Domain 2 |  |
| solid.CIel33 | solid.Jel^(-2/3)*solid.Cel33 | 1 | Isochoric elastic Cauchy-Green tensor, local coordinate system, 33 component | Domain 2 |  |
| solid.I1CIel | solid.Jel^(-2/3)*solid.I1Cel | 1 | First invariant of isochoric elastic right Cauchy-Green tensor | Domain 2 |  |
| solid.I2CIel | solid.Jel^(-4/3)*solid.I2Cel | 1 | Second invariant of isochoric elastic right Cauchy-Green tensor | Domain 2 |  |
| solid.I1eel | 0.5*(-3+solid.I1Cel) | 1 | First invariant of elastic Green-Lagrange strain tensor | Domain 2 |  |
| solid.I2eel | 0.25*(3+solid.I2Cel-2*solid.I1Cel) | 1 | Second invariant of elastic Green-Lagrange strain tensor | Domain 2 |  |
| solid.I3eel | 0.125*(-1+solid.I3Cel-solid.I2Cel+solid.I1Cel) | 1 | Third invariant of elastic Green-Lagrange strain tensor | Domain 2 |  |
| solid.I1eIel | 0.5*(-3+solid.I1CIel) | 1 | First invariant of isochoric elastic Green-Lagrange strain tensor | Domain 2 |  |
| solid.I2eIel | 0.25*(3+solid.I2CIel-2*solid.I1CIel) | 1 | Second invariant of isochoric elastic Green-Lagrange strain tensor | Domain 2 |  |
| solid.I3eIel | 0.125*(solid.I1CIel-solid.I2CIel) | 1 | Third invariant of isochoric elastic Green-Lagrange strain tensor | Domain 2 |  |
| solid.stchIelp1 | solid.stchelp1/solid.Jel^(1/3) | 1 | First principal isochoric elastic stretch | Domain 2 |  |
| solid.stchIelp2 | solid.stchelp2/solid.Jel^(1/3) | 1 | Second principal isochoric elastic stretch | Domain 2 |  |
| solid.stchIelp3 | solid.stchelp3/solid.Jel^(1/3) | 1 | Third principal isochoric elastic stretch | Domain 2 |  |
| solid.Wk | 0.5*(u2TIME^2+v2TIME^2+w2TIME^2)*solid.rho | J/m³ | Kinetic energy density | Domain 2 | + operation |
| solid.Ws_tot | root.comp1.solid.hmm1.int10(solid.Ws) | J | Total elastic strain energy | Global | + operation |
| solid.Wh_tot | root.comp1.solid.hmm1.int11(solid.Ws) | J | Total stored energy | Global | + operation |
| solid.PxX | solid.FdxX*solid.SX+solid.FdxY*solid.SXY+solid.FdxZ*solid.SXZ | N/m² | First Piola-Kirchhoff stress, xX component | Domain 2 |  |
| solid.PyX | solid.FdyX*solid.SX+solid.FdyY*solid.SXY+solid.FdyZ*solid.SXZ | N/m² | First Piola-Kirchhoff stress, yX component | Domain 2 |  |
| solid.PzX | solid.FdzX*solid.SX+solid.FdzY*solid.SXY+solid.FdzZ*solid.SXZ | N/m² | First Piola-Kirchhoff stress, zX component | Domain 2 |  |
| solid.PxY | solid.FdxX*solid.SXY+solid.FdxY*solid.SY+solid.FdxZ*solid.SYZ | N/m² | First Piola-Kirchhoff stress, xY component | Domain 2 |  |
| solid.PyY | solid.FdyX*solid.SXY+solid.FdyY*solid.SY+solid.FdyZ*solid.SYZ | N/m² | First Piola-Kirchhoff stress, yY component | Domain 2 |  |
| solid.PzY | solid.FdzX*solid.SXY+solid.FdzY*solid.SY+solid.FdzZ*solid.SYZ | N/m² | First Piola-Kirchhoff stress, zY component | Domain 2 |  |
| solid.PxZ | solid.FdxX*solid.SXZ+solid.FdxY*solid.SYZ+solid.FdxZ*solid.SZ | N/m² | First Piola-Kirchhoff stress, xZ component | Domain 2 |  |
| solid.PyZ | solid.FdyX*solid.SXZ+solid.FdyY*solid.SYZ+solid.FdyZ*solid.SZ | N/m² | First Piola-Kirchhoff stress, yZ component | Domain 2 |  |
| solid.PzZ | solid.FdzX*solid.SXZ+solid.FdzY*solid.SYZ+solid.FdzZ*solid.SZ | N/m² | First Piola-Kirchhoff stress, zZ component | Domain 2 |  |
| solid.sx | ((solid.Fdlx1*solid.Sl11+solid.Fdlx2*solid.Sl12+solid.Fdlx3*solid.Sl13)*solid.Fdlx1+(solid.Fdlx1*solid.Sl12+solid.Fdlx2*solid.Sl22+solid.Fdlx3*solid.Sl23)*solid.Fdlx2+(solid.Fdlx1*solid.Sl13+solid.Fdlx2*solid.Sl23+solid.Fdlx3*solid.Sl33)*solid.Fdlx3)/solid.J | N/m² | Stress tensor, x component | Domain 2 |  |
| solid.sxy | ((solid.Fdlx1*solid.Sl11+solid.Fdlx2*solid.Sl12+solid.Fdlx3*solid.Sl13)*solid.Fdly1+(solid.Fdlx1*solid.Sl12+solid.Fdlx2*solid.Sl22+solid.Fdlx3*solid.Sl23)*solid.Fdly2+(solid.Fdlx1*solid.Sl13+solid.Fdlx2*solid.Sl23+solid.Fdlx3*solid.Sl33)*solid.Fdly3)/solid.J | N/m² | Stress tensor, xy component | Domain 2 |  |
| solid.sxz | ((solid.Fdlx1*solid.Sl11+solid.Fdlx2*solid.Sl12+solid.Fdlx3*solid.Sl13)*solid.Fdlz1+(solid.Fdlx1*solid.Sl12+solid.Fdlx2*solid.Sl22+solid.Fdlx3*solid.Sl23)*solid.Fdlz2+(solid.Fdlx1*solid.Sl13+solid.Fdlx2*solid.Sl23+solid.Fdlx3*solid.Sl33)*solid.Fdlz3)/solid.J | N/m² | Stress tensor, xz component | Domain 2 |  |
| solid.sy | ((solid.Fdly1*solid.Sl11+solid.Fdly2*solid.Sl12+solid.Fdly3*solid.Sl13)*solid.Fdly1+(solid.Fdly1*solid.Sl12+solid.Fdly2*solid.Sl22+solid.Fdly3*solid.Sl23)*solid.Fdly2+(solid.Fdly1*solid.Sl13+solid.Fdly2*solid.Sl23+solid.Fdly3*solid.Sl33)*solid.Fdly3)/solid.J | N/m² | Stress tensor, y component | Domain 2 |  |
| solid.syz | ((solid.Fdly1*solid.Sl11+solid.Fdly2*solid.Sl12+solid.Fdly3*solid.Sl13)*solid.Fdlz1+(solid.Fdly1*solid.Sl12+solid.Fdly2*solid.Sl22+solid.Fdly3*solid.Sl23)*solid.Fdlz2+(solid.Fdly1*solid.Sl13+solid.Fdly2*solid.Sl23+solid.Fdly3*solid.Sl33)*solid.Fdlz3)/solid.J | N/m² | Stress tensor, yz component | Domain 2 |  |
| solid.sz | ((solid.Fdlz1*solid.Sl11+solid.Fdlz2*solid.Sl12+solid.Fdlz3*solid.Sl13)*solid.Fdlz1+(solid.Fdlz1*solid.Sl12+solid.Fdlz2*solid.Sl22+solid.Fdlz3*solid.Sl23)*solid.Fdlz2+(solid.Fdlz1*solid.Sl13+solid.Fdlz2*solid.Sl23+solid.Fdlz3*solid.Sl33)*solid.Fdlz3)/solid.J | N/m² | Stress tensor, z component | Domain 2 |  |
| solid.sdevx | solid.sx-(solid.sx+solid.sy+solid.sz)/3 | N/m² | Deviatoric stress tensor, x component | Domain 2 |  |
| solid.sdevxy | solid.sxy | N/m² | Deviatoric stress tensor, xy component | Domain 2 |  |
| solid.sdevxz | solid.sxz | N/m² | Deviatoric stress tensor, xz component | Domain 2 |  |
| solid.sdevy | solid.sy-(solid.sx+solid.sy+solid.sz)/3 | N/m² | Deviatoric stress tensor, y component | Domain 2 |  |
| solid.sdevyz | solid.syz | N/m² | Deviatoric stress tensor, yz component | Domain 2 |  |
| solid.sdevz | solid.sz-(solid.sx+solid.sy+solid.sz)/3 | N/m² | Deviatoric stress tensor, z component | Domain 2 |  |
| solid.SdevX | solid.SX-(solid.SX+solid.SY+solid.SZ)/3 | N/m² | Deviatoric second Piola-Kirchhoff stress, X component | Domain 2 |  |
| solid.SdevXY | solid.SXY | N/m² | Deviatoric second Piola-Kirchhoff stress, XY component | Domain 2 |  |
| solid.SdevXZ | solid.SXZ | N/m² | Deviatoric second Piola-Kirchhoff stress, XZ component | Domain 2 |  |
| solid.SdevY | solid.SY-(solid.SX+solid.SY+solid.SZ)/3 | N/m² | Deviatoric second Piola-Kirchhoff stress, Y component | Domain 2 |  |
| solid.SdevYZ | solid.SYZ | N/m² | Deviatoric second Piola-Kirchhoff stress, YZ component | Domain 2 |  |
| solid.SdevZ | solid.SZ-(solid.SX+solid.SY+solid.SZ)/3 | N/m² | Deviatoric second Piola-Kirchhoff stress, Z component | Domain 2 |  |
| solid.I1s | solid.sl11+solid.sl22+solid.sl33 | N/m² | First principal invariant of stress | Domain 2 |  |
| solid.I2s | 0.5*(solid.I1s^2-solid.sl11^2-2*solid.sl12^2-2*solid.sl13^2-solid.sl22^2-2*solid.sl23^2-solid.sl33^2) | kg²/(m²·s⁴) | Second principal invariant of stress | Domain 2 |  |
| solid.I3s | solid.sl11*solid.sl22*solid.sl33+2*solid.sl12*solid.sl23*solid.sl13-solid.sl11*solid.sl23^2-solid.sl12^2*solid.sl33-solid.sl22*solid.sl13^2 | kg³/(m³·s⁶) | Third principal invariant of stress | Domain 2 |  |
| solid.II2s | 0.5*((solid.sl11-(solid.sl11+solid.sl22+solid.sl33)/3)^2+2*solid.sl12^2+2*solid.sl13^2+(solid.sl22-(solid.sl11+solid.sl22+solid.sl33)/3)^2+2*solid.sl23^2+(solid.sl33-(solid.sl11+solid.sl22+solid.sl33)/3)^2) | kg²/(m²·s⁴) | Second invariant of stress deviator | Domain 2 |  |
| solid.II3s | (solid.sl11-(solid.sl11+solid.sl22+solid.sl33)/3)*(solid.sl22-(solid.sl11+solid.sl22+solid.sl33)/3)*(solid.sl33-(solid.sl11+solid.sl22+solid.sl33)/3)+2*solid.sl12*solid.sl23*solid.sl13-(solid.sl11-(solid.sl11+solid.sl22+solid.sl33)/3)*solid.sl23^2-solid.sl12^2*(solid.sl33-(solid.sl11+solid.sl22+solid.sl33)/3)-(solid.sl22-(solid.sl11+solid.sl22+solid.sl33)/3)*solid.sl13^2 | kg³/(m³·s⁶) | Third invariant of stress deviator | Domain 2 |  |
| solid.thetaL | atan2(sqrt(max(0.14814814814814814*solid.II2s^3-solid.II3s^2,eps)),solid.II3s)/3 | rad | Lode angle | Domain 2 |  |
| solid.curlUX | solid.gradUzY-solid.gradUyZ | 1 | Curl of displacement, X component | Domain 2 |  |
| solid.curlUY | solid.gradUxZ-solid.gradUzX | 1 | Curl of displacement, Y component | Domain 2 |  |
| solid.curlUZ | solid.gradUyX-solid.gradUxY | 1 | Curl of displacement, Z component | Domain 2 |  |
| solid.tresca | solid.sp1-solid.sp3 | N/m² | Tresca stress | Domain 2 |  |
| solid.RFx | reacf(u2) | N | Reaction force, x component | Domain 2 |  |
| solid.RFy | reacf(v2) | N | Reaction force, y component | Domain 2 |  |
| solid.RFz | reacf(w2) | N | Reaction force, z component | Domain 2 |  |
| solid.RMx | solid.RFz*(y-solid.refpnty)-solid.RFy*(z-solid.refpntz) | N·m | Reaction moment, x component | Domain 2 |  |
| solid.RMy | -solid.RFz*(x-solid.refpntx)+solid.RFx*(z-solid.refpntz) | N·m | Reaction moment, y component | Domain 2 |  |
| solid.RMz | solid.RFy*(x-solid.refpntx)-solid.RFx*(y-solid.refpnty) | N·m | Reaction moment, z component | Domain 2 |  |
| solid.Tax | solid.sx*solid.nx+solid.sxy*solid.ny+solid.sxz*solid.nz | N/m² | Traction (force/area), x component | Boundaries 4–7, 9–11, 13–15, 17 |  |
| solid.Tay | solid.sxy*solid.nx+solid.sy*solid.ny+solid.syz*solid.nz | N/m² | Traction (force/area), y component | Boundaries 4–7, 9–11, 13–15, 17 |  |
| solid.Taz | solid.sxz*solid.nx+solid.syz*solid.ny+solid.sz*solid.nz | N/m² | Traction (force/area), z component | Boundaries 4–7, 9–11, 13–15, 17 |  |
| solid.stn | (solid.sx*solid.nx+solid.sxy*solid.ny+solid.sxz*solid.nz)*solid.nx+(solid.sxy*solid.nx+solid.sy*solid.ny+solid.syz*solid.nz)*solid.ny+(solid.sxz*solid.nx+solid.syz*solid.ny+solid.sz*solid.nz)*solid.nz | N/m² | Normal stress | Boundaries 4–7, 9–11, 13–15, 17 |  |
| solid.Tanx | ((solid.sx*solid.nx+solid.sxy*solid.ny+solid.sxz*solid.nz)*solid.nx+(solid.sxy*solid.nx+solid.sy*solid.ny+solid.syz*solid.nz)*solid.ny+(solid.sxz*solid.nx+solid.syz*solid.ny+solid.sz*solid.nz)*solid.nz)*solid.nx | N/m² | Normal component of traction, x component | Boundaries 4–7, 9–11, 13–15, 17 |  |
| solid.Tany | ((solid.sx*solid.nx+solid.sxy*solid.ny+solid.sxz*solid.nz)*solid.nx+(solid.sxy*solid.nx+solid.sy*solid.ny+solid.syz*solid.nz)*solid.ny+(solid.sxz*solid.nx+solid.syz*solid.ny+solid.sz*solid.nz)*solid.nz)*solid.ny | N/m² | Normal component of traction, y component | Boundaries 4–7, 9–11, 13–15, 17 |  |
| solid.Tanz | ((solid.sx*solid.nx+solid.sxy*solid.ny+solid.sxz*solid.nz)*solid.nx+(solid.sxy*solid.nx+solid.sy*solid.ny+solid.syz*solid.nz)*solid.ny+(solid.sxz*solid.nx+solid.syz*solid.ny+solid.sz*solid.nz)*solid.nz)*solid.nz | N/m² | Normal component of traction, z component | Boundaries 4–7, 9–11, 13–15, 17 |  |
| solid.Tatx | solid.sx*solid.nx+solid.sxy*solid.ny+solid.sxz*solid.nz-((solid.sx*solid.nx+solid.sxy*solid.ny+solid.sxz*solid.nz)*solid.nx+(solid.sxy*solid.nx+solid.sy*solid.ny+solid.syz*solid.nz)*solid.ny+(solid.sxz*solid.nx+solid.syz*solid.ny+solid.sz*solid.nz)*solid.nz)*solid.nx | N/m² | Shear component of traction, x component | Boundaries 4–7, 9–11, 13–15, 17 |  |
| solid.Taty | solid.sxy*solid.nx+solid.sy*solid.ny+solid.syz*solid.nz-((solid.sx*solid.nx+solid.sxy*solid.ny+solid.sxz*solid.nz)*solid.nx+(solid.sxy*solid.nx+solid.sy*solid.ny+solid.syz*solid.nz)*solid.ny+(solid.sxz*solid.nx+solid.syz*solid.ny+solid.sz*solid.nz)*solid.nz)*solid.ny | N/m² | Shear component of traction, y component | Boundaries 4–7, 9–11, 13–15, 17 |  |
| solid.Tatz | solid.sxz*solid.nx+solid.syz*solid.ny+solid.sz*solid.nz-((solid.sx*solid.nx+solid.sxy*solid.ny+solid.sxz*solid.nz)*solid.nx+(solid.sxy*solid.nx+solid.sy*solid.ny+solid.syz*solid.nz)*solid.ny+(solid.sxz*solid.nx+solid.syz*solid.ny+solid.sz*solid.nz)*solid.nz)*solid.nz | N/m² | Shear component of traction, z component | Boundaries 4–7, 9–11, 13–15, 17 |  |
| solid.stt | sqrt((solid.sx*solid.nx+solid.sxy*solid.ny+solid.sxz*solid.nz-((solid.sx*solid.nx+solid.sxy*solid.ny+solid.sxz*solid.nz)*solid.nx+(solid.sxy*solid.nx+solid.sy*solid.ny+solid.syz*solid.nz)*solid.ny+(solid.sxz*solid.nx+solid.syz*solid.ny+solid.sz*solid.nz)*solid.nz)*solid.nx)^2+(solid.sxy*solid.nx+solid.sy*solid.ny+solid.syz*solid.nz-((solid.sx*solid.nx+solid.sxy*solid.ny+solid.sxz*solid.nz)*solid.nx+(solid.sxy*solid.nx+solid.sy*solid.ny+solid.syz*solid.nz)*solid.ny+(solid.sxz*solid.nx+solid.syz*solid.ny+solid.sz*solid.nz)*solid.nz)*solid.ny)^2+(solid.sxz*solid.nx+solid.syz*solid.ny+solid.sz*solid.nz-((solid.sx*solid.nx+solid.sxy*solid.ny+solid.sxz*solid.nz)*solid.nx+(solid.sxy*solid.nx+solid.sy*solid.ny+solid.syz*solid.nz)*solid.ny+(solid.sxz*solid.nx+solid.syz*solid.ny+solid.sz*solid.nz)*solid.nz)*solid.nz)^2) | N/m² | Shear stress | Boundaries 4–7, 9–11, 13–15, 17 |  |
| solid.Qh_tot | root.comp1.solid.hmm1.int12(solid.Qh) | W | Total power dissipation | Global | + operation |
| solid.IX | -solid.SX*solid.u_tX-solid.SXY*solid.u_tY-solid.SXZ*solid.u_tZ | W/m² | Mechanical energy flux, X component | Domain 2 |  |
| solid.IY | -solid.SXY*solid.u_tX-solid.SY*solid.u_tY-solid.SYZ*solid.u_tZ | W/m² | Mechanical energy flux, Y component | Domain 2 |  |
| solid.IZ | -solid.SXZ*solid.u_tX-solid.SYZ*solid.u_tY-solid.SZ*solid.u_tZ | W/m² | Mechanical energy flux, Z component | Domain 2 |  |
| solid.nI | nX*solid.IX+nY*solid.IY+nZ*solid.IZ | W/m² | Outward mechanical energy flux | Boundaries 4–5, 7, 9–11, 13–15, 17 | Meta |
| solid.hmm1.nI | nX*solid.IX+nY*solid.IY+nZ*solid.IZ | W/m² | Outward mechanical energy flux | Boundaries 4–7, 9–11, 13–15, 17 | Meta |

#### Shape functions

| **Name** | **Shape function** | **Unit** | **Description** | **Shape frame** | **Selection** | **Details** |
| --- | --- | --- | --- | --- | --- | --- |
| u2 | Lagrange (Quadratic) | m | Displacement field, X component | Material | Domain 2 |  |
| v2 | Lagrange (Quadratic) | m | Displacement field, Y component | Material | Domain 2 |  |
| w2 | Lagrange (Quadratic) | m | Displacement field, Z component | Material | Domain 2 |  |
| solid.pw | Lagrange (Linear) | N/m² | Auxiliary pressure | Material | Domain 2 |  |
| solid.pw | Lagrange (Linear) | N/m² | Auxiliary pressure | Material | Boundaries 4–7, 9–11, 13–15, 17 | Slit |

#### Weak expressions

| **Weak expression** | **Integration order** | **Integration frame** | **Selection** |
| --- | --- | --- | --- |
| (solid.pw-solid.pm)*test(solid.pw)/solid.K | 4 | Material | Domain 2 |
| -solid.Sl11*test(solid.el11)-2*solid.Sl12*test(solid.el12)-2*solid.Sl13*test(solid.el13)-solid.Sl22*test(solid.el22)-2*solid.Sl23*test(solid.el23)-solid.Sl33*test(solid.el33) | 4 | Material | Domain 2 |
| solid.rho*(-d(u2TIME,TIME)*test(u2)-d(v2TIME,TIME)*test(v2)-d(w2TIME,TIME)*test(w2)) | 4 | Material | Domain 2 |

- - 1. Hyperelastic Material 2


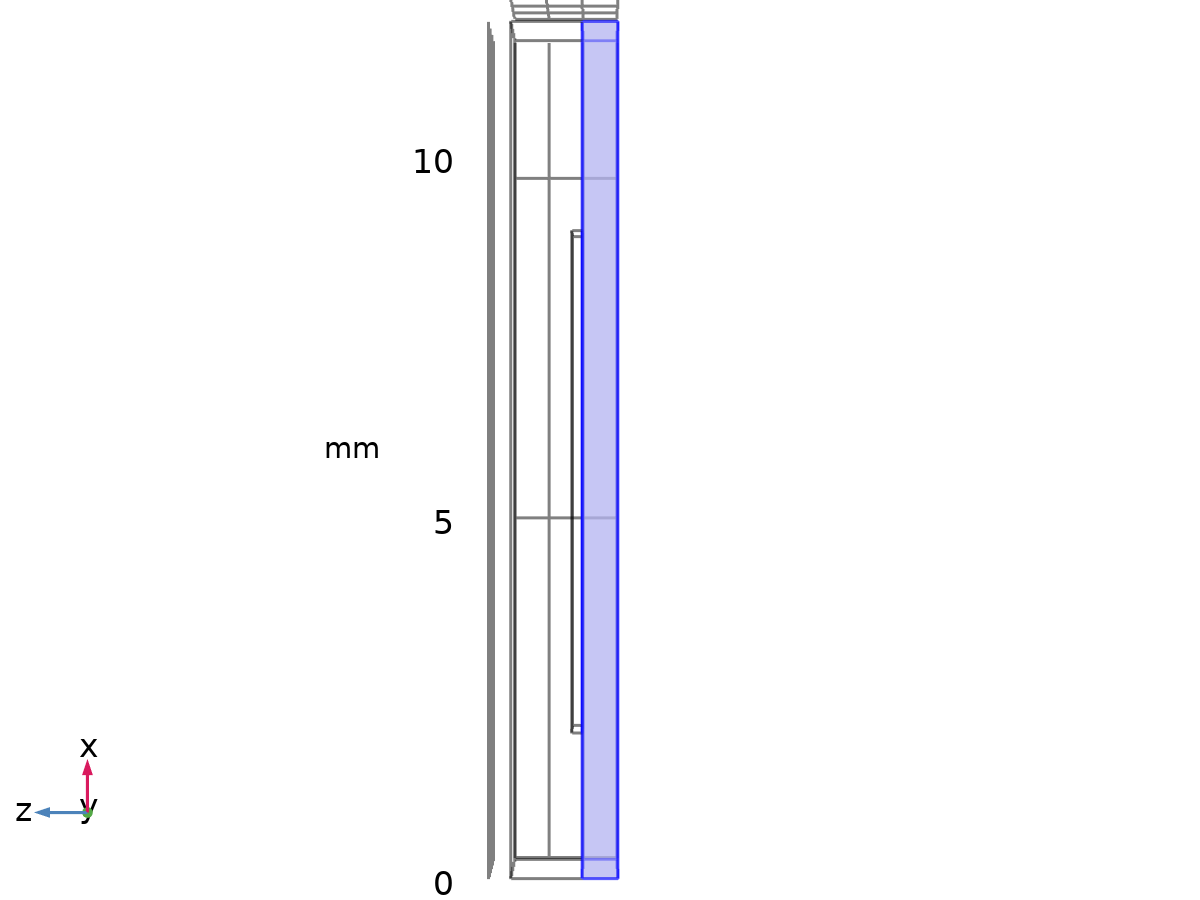


Hyperelastic Material 2

Selection

| Geometric entity level | Domain |
| --- | --- |
| Selection | Geometry geom1: Dimension 3: Domain 1 |

Equations


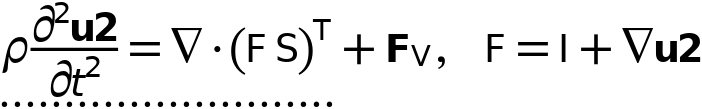


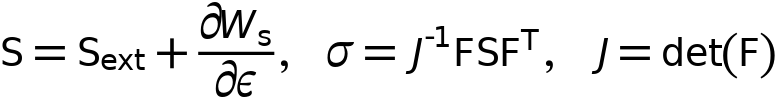


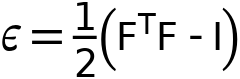


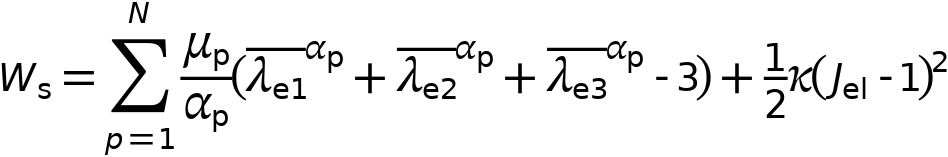


#### Hyperelastic material

Settings

| **Description** | **Value** |
| --- | --- |
| Material model | Ogden |
| Compressibility | Nearly incompressible material, quadratic volumetric strain energy |
| Bulk modulus | 5.5e4 |
| Density | User defined |
| Density | 1070 |

| **p** | **Shear modulus** | **Alpha parameter** |
| --- | --- | --- |
| 1 | 1241 | 3.039 |
| 2 | 7.8789e-6 | 13.023 |

#### Coordinate system selection

Settings

| **Description** | **Value** |
| --- | --- |
| Coordinate system | Global coordinate system |

#### Variables

| **Name** | **Expression** | **Unit** | **Description** | **Selection** | **Details** |
| --- | --- | --- | --- | --- | --- |
| solid.u_tX | u2TIME | m/s | Velocity, X component | Domain 1 |  |
| solid.u_tY | v2TIME | m/s | Velocity, Y component | Domain 1 |  |
| solid.u_tZ | w2TIME | m/s | Velocity, Z component | Domain 1 |  |
| solid.Wk_tot | root.comp1.solid.hmm2.int13(solid.Wk) | J | Total kinetic energy | Global | + operation |
| solid.rho | 1070 | kg/m³ | Density | Domain 1 | * operation |
| u2TXt | u2TXTIME | 1/s | Tangential gradient of u2, X component, first time derivative | Boundaries 1–3, 6, 8, 12, 16 |  |
| u2TYt | u2TYTIME | 1/s | Tangential gradient of u2, Y component, first time derivative | Boundaries 1–3, 6, 8, 12, 16 |  |
| u2TZt | u2TZTIME | 1/s | Tangential gradient of u2, Z component, first time derivative | Boundaries 1–3, 6, 8, 12, 16 |  |
| u2TXtt | u2TXTIMETIME | 1/s² | Tangential gradient of u2, X component, second time derivative | Boundaries 1–3, 6, 8, 12, 16 |  |
| u2TYtt | u2TYTIMETIME | 1/s² | Tangential gradient of u2, Y component, second time derivative | Boundaries 1–3, 6, 8, 12, 16 |  |
| u2TZtt | u2TZTIMETIME | 1/s² | Tangential gradient of u2, Z component, second time derivative | Boundaries 1–3, 6, 8, 12, 16 |  |
| u2Xt | u2XTIME | 1/s | Gradient of u2, X component, first time derivative | Domain 1 |  |
| u2Yt | u2YTIME | 1/s | Gradient of u2, Y component, first time derivative | Domain 1 |  |
| u2Zt | u2ZTIME | 1/s | Gradient of u2, Z component, first time derivative | Domain 1 |  |
| u2Xtt | u2XTIMETIME | 1/s² | Gradient of u2, X component, second time derivative | Domain 1 |  |
| u2Ytt | u2YTIMETIME | 1/s² | Gradient of u2, Y component, second time derivative | Domain 1 |  |
| u2Ztt | u2ZTIMETIME | 1/s² | Gradient of u2, Z component, second time derivative | Domain 1 |  |
| v2TXt | v2TXTIME | 1/s | Tangential gradient of v2, X component, first time derivative | Boundaries 1–3, 6, 8, 12, 16 |  |
| v2TYt | v2TYTIME | 1/s | Tangential gradient of v2, Y component, first time derivative | Boundaries 1–3, 6, 8, 12, 16 |  |
| v2TZt | v2TZTIME | 1/s | Tangential gradient of v2, Z component, first time derivative | Boundaries 1–3, 6, 8, 12, 16 |  |
| v2TXtt | v2TXTIMETIME | 1/s² | Tangential gradient of v2, X component, second time derivative | Boundaries 1–3, 6, 8, 12, 16 |  |
| v2TYtt | v2TYTIMETIME | 1/s² | Tangential gradient of v2, Y component, second time derivative | Boundaries 1–3, 6, 8, 12, 16 |  |
| v2TZtt | v2TZTIMETIME | 1/s² | Tangential gradient of v2, Z component, second time derivative | Boundaries 1–3, 6, 8, 12, 16 |  |
| v2Xt | v2XTIME | 1/s | Gradient of v2, X component, first time derivative | Domain 1 |  |
| v2Yt | v2YTIME | 1/s | Gradient of v2, Y component, first time derivative | Domain 1 |  |
| v2Zt | v2ZTIME | 1/s | Gradient of v2, Z component, first time derivative | Domain 1 |  |
| v2Xtt | v2XTIMETIME | 1/s² | Gradient of v2, X component, second time derivative | Domain 1 |  |
| v2Ytt | v2YTIMETIME | 1/s² | Gradient of v2, Y component, second time derivative | Domain 1 |  |
| v2Ztt | v2ZTIMETIME | 1/s² | Gradient of v2, Z component, second time derivative | Domain 1 |  |
| w2TXt | w2TXTIME | 1/s | Tangential gradient of w2, X component, first time derivative | Boundaries 1–3, 6, 8, 12, 16 |  |
| w2TYt | w2TYTIME | 1/s | Tangential gradient of w2, Y component, first time derivative | Boundaries 1–3, 6, 8, 12, 16 |  |
| w2TZt | w2TZTIME | 1/s | Tangential gradient of w2, Z component, first time derivative | Boundaries 1–3, 6, 8, 12, 16 |  |
| w2TXtt | w2TXTIMETIME | 1/s² | Tangential gradient of w2, X component, second time derivative | Boundaries 1–3, 6, 8, 12, 16 |  |
| w2TYtt | w2TYTIMETIME | 1/s² | Tangential gradient of w2, Y component, second time derivative | Boundaries 1–3, 6, 8, 12, 16 |  |
| w2TZtt | w2TZTIMETIME | 1/s² | Tangential gradient of w2, Z component, second time derivative | Boundaries 1–3, 6, 8, 12, 16 |  |
| w2Xt | w2XTIME | 1/s | Gradient of w2, X component, first time derivative | Domain 1 |  |
| w2Yt | w2YTIME | 1/s | Gradient of w2, Y component, first time derivative | Domain 1 |  |
| w2Zt | w2ZTIME | 1/s | Gradient of w2, Z component, first time derivative | Domain 1 |  |
| w2Xtt | w2XTIMETIME | 1/s² | Gradient of w2, X component, second time derivative | Domain 1 |  |
| w2Ytt | w2YTIMETIME | 1/s² | Gradient of w2, Y component, second time derivative | Domain 1 |  |
| w2Ztt | w2ZTIMETIME | 1/s² | Gradient of w2, Z component, second time derivative | Domain 1 |  |
| u2t | u2TIME | m/s | Structural velocity field, X component | Domain 1 |  |
| v2t | v2TIME | m/s | Structural velocity field, Y component | Domain 1 |  |
| w2t | w2TIME | m/s | Structural velocity field, Z component | Domain 1 |  |
| u2tt | u2TIMETIME | m/s² | Acceleration field, X component | Domain 1 |  |
| v2tt | v2TIMETIME | m/s² | Acceleration field, Y component | Domain 1 |  |
| w2tt | w2TIMETIME | m/s² | Acceleration field, Z component | Domain 1 |  |
| solid.gradUxX | u2X | 1 | Displacement gradient, xX component | Domain 1 |  |
| solid.gradUyX | v2X | 1 | Displacement gradient, yX component | Domain 1 |  |
| solid.gradUzX | w2X | 1 | Displacement gradient, zX component | Domain 1 |  |
| solid.gradUxY | u2Y | 1 | Displacement gradient, xY component | Domain 1 |  |
| solid.gradUyY | v2Y | 1 | Displacement gradient, yY component | Domain 1 |  |
| solid.gradUzY | w2Y | 1 | Displacement gradient, zY component | Domain 1 |  |
| solid.gradUxZ | u2Z | 1 | Displacement gradient, xZ component | Domain 1 |  |
| solid.gradUyZ | v2Z | 1 | Displacement gradient, yZ component | Domain 1 |  |
| solid.gradUzZ | w2Z | 1 | Displacement gradient, zZ component | Domain 1 |  |
| solid.elogxx | log(solid.stchp1)*solid.LW11^2+log(solid.stchp2)*solid.LW12^2+log(solid.stchp3)*solid.LW13^2 | 1 | Logarithmic strain tensor, xx component | Domain 1 |  |
| solid.elogxy | log(solid.stchp1)*solid.LW11*solid.LW21+log(solid.stchp2)*solid.LW12*solid.LW22+log(solid.stchp3)*solid.LW13*solid.LW23 | 1 | Logarithmic strain tensor, xy component | Domain 1 |  |
| solid.elogxz | log(solid.stchp1)*solid.LW11*solid.LW31+log(solid.stchp2)*solid.LW12*solid.LW32+log(solid.stchp3)*solid.LW13*solid.LW33 | 1 | Logarithmic strain tensor, xz component | Domain 1 |  |
| solid.elogyy | log(solid.stchp1)*solid.LW21^2+log(solid.stchp2)*solid.LW22^2+log(solid.stchp3)*solid.LW23^2 | 1 | Logarithmic strain tensor, yy component | Domain 1 |  |
| solid.elogyz | log(solid.stchp1)*solid.LW21*solid.LW31+log(solid.stchp2)*solid.LW22*solid.LW32+log(solid.stchp3)*solid.LW23*solid.LW33 | 1 | Logarithmic strain tensor, yz component | Domain 1 |  |
| solid.elogzz | log(solid.stchp1)*solid.LW31^2+log(solid.stchp2)*solid.LW32^2+log(solid.stchp3)*solid.LW33^2 | 1 | Logarithmic strain tensor, zz component | Domain 1 |  |
| solid.FdxX | 1+solid.gradUxX | 1 | Deformation gradient, xX component | Domain 1 |  |
| solid.FdyX | solid.gradUyX | 1 | Deformation gradient, yX component | Domain 1 |  |
| solid.FdzX | solid.gradUzX | 1 | Deformation gradient, zX component | Domain 1 |  |
| solid.FdxY | solid.gradUxY | 1 | Deformation gradient, xY component | Domain 1 |  |
| solid.FdyY | 1+solid.gradUyY | 1 | Deformation gradient, yY component | Domain 1 |  |
| solid.FdzY | solid.gradUzY | 1 | Deformation gradient, zY component | Domain 1 |  |
| solid.FdxZ | solid.gradUxZ | 1 | Deformation gradient, xZ component | Domain 1 |  |
| solid.FdyZ | solid.gradUyZ | 1 | Deformation gradient, yZ component | Domain 1 |  |
| solid.FdzZ | 1+solid.gradUzZ | 1 | Deformation gradient, zZ component | Domain 1 |  |
| solid.Fdlx1 | solid.FdxX | 1 | Deformation gradient, local, x1 component | Domain 1 |  |
| solid.Fdly1 | solid.FdyX | 1 | Deformation gradient, local, y1 component | Domain 1 |  |
| solid.Fdlz1 | solid.FdzX | 1 | Deformation gradient, local, z1 component | Domain 1 |  |
| solid.Fdlx2 | solid.FdxY | 1 | Deformation gradient, local, x2 component | Domain 1 |  |
| solid.Fdly2 | solid.FdyY | 1 | Deformation gradient, local, y2 component | Domain 1 |  |
| solid.Fdlz2 | solid.FdzY | 1 | Deformation gradient, local, z2 component | Domain 1 |  |
| solid.Fdlx3 | solid.FdxZ | 1 | Deformation gradient, local, x3 component | Domain 1 |  |
| solid.Fdly3 | solid.FdyZ | 1 | Deformation gradient, local, y3 component | Domain 1 |  |
| solid.Fdlz3 | solid.FdzZ | 1 | Deformation gradient, local, z3 component | Domain 1 |  |
| solid.K | -d(solid.pm,solid.J) | N/m² | Bulk modulus | Domain 1 | * operation |
| solid.Eequ | 4.5*solid.kappa*(solid.mup1*solid.alphap1+solid.mup2*solid.alphap2)/(3*solid.kappa+0.5*solid.mup1*solid.alphap1+0.5*solid.mup2*solid.alphap2) | Pa | Equivalent Young's modulus | Domain 1 |  |
| solid.nuequ | (3*solid.kappa-solid.mup1*solid.alphap1-solid.mup2*solid.alphap2)/(6*solid.kappa+solid.mup1*solid.alphap1+solid.mup2*solid.alphap2) | 1 | Equivalent Poisson's ratio | Domain 1 |  |
| solid.cp | sqrt((solid.kappa+2*(solid.mup1*solid.alphap1+solid.mup2*solid.alphap2)/3)/solid.rho) | m/s | Equivalent speed of pressure wave | Domain 1 |  |
| solid.cs | sqrt(0.5*(solid.mup1*solid.alphap1+solid.mup2*solid.alphap2)/solid.rho) | m/s | Equivalent speed of shear wave | Domain 1 |  |
| solid.eXX | solid.el11 | 1 | Strain tensor, XX component | Domain 1 | + operation |
| solid.eXY | solid.el12 | 1 | Strain tensor, XY component | Domain 1 | + operation |
| solid.eXZ | solid.el13 | 1 | Strain tensor, XZ component | Domain 1 | + operation |
| solid.eYY | solid.el22 | 1 | Strain tensor, YY component | Domain 1 | + operation |
| solid.eYZ | solid.el23 | 1 | Strain tensor, YZ component | Domain 1 | + operation |
| solid.eZZ | solid.el33 | 1 | Strain tensor, ZZ component | Domain 1 | + operation |
| solid.el11 | 0.5*(-1+solid.Cl11) | 1 | Strain tensor, local coordinate system, 11 component | Domain 1 | + operation |
| solid.el12 | 0.5*solid.Cl12 | 1 | Strain tensor, local coordinate system, 12 component | Domain 1 | + operation |
| solid.el13 | 0.5*solid.Cl13 | 1 | Strain tensor, local coordinate system, 13 component | Domain 1 | + operation |
| solid.el22 | 0.5*(-1+solid.Cl22) | 1 | Strain tensor, local coordinate system, 22 component | Domain 1 | + operation |
| solid.el23 | 0.5*solid.Cl23 | 1 | Strain tensor, local coordinate system, 23 component | Domain 1 | + operation |
| solid.el33 | 0.5*(-1+solid.Cl33) | 1 | Strain tensor, local coordinate system, 33 component | Domain 1 | + operation |
| solid.J | sqrt(solid.Cl11*solid.Cl22*solid.Cl33+2*solid.Cl12*solid.Cl23*solid.Cl13-solid.Cl11*solid.Cl23^2-solid.Cl12^2*solid.Cl33-solid.Cl22*solid.Cl13^2) | 1 | Volume ratio | Domain 1 |  |
| solid.eX | solid.eXX | 1 | Strain tensor, X component | Domain 1 |  |
| solid.eY | solid.eYY | 1 | Strain tensor, Y component | Domain 1 |  |
| solid.eZ | solid.eZZ | 1 | Strain tensor, Z component | Domain 1 |  |
| solid.Cl11 | solid.Fdlx1^2+solid.Fdly1^2+solid.Fdlz1^2 | 1 | Cauchy-Green tensor, local coordinate system, 11 component | Domain 1 |  |
| solid.Cl12 | solid.Fdlx1*solid.Fdlx2+solid.Fdly1*solid.Fdly2+solid.Fdlz1*solid.Fdlz2 | 1 | Cauchy-Green tensor, local coordinate system, 12 component | Domain 1 |  |
| solid.Cl13 | solid.Fdlx1*solid.Fdlx3+solid.Fdly1*solid.Fdly3+solid.Fdlz1*solid.Fdlz3 | 1 | Cauchy-Green tensor, local coordinate system, 13 component | Domain 1 |  |
| solid.Cl22 | solid.Fdlx2^2+solid.Fdly2^2+solid.Fdlz2^2 | 1 | Cauchy-Green tensor, local coordinate system, 22 component | Domain 1 |  |
| solid.Cl23 | solid.Fdlx2*solid.Fdlx3+solid.Fdly2*solid.Fdly3+solid.Fdlz2*solid.Fdlz3 | 1 | Cauchy-Green tensor, local coordinate system, 23 component | Domain 1 |  |
| solid.Cl33 | solid.Fdlx3^2+solid.Fdly3^2+solid.Fdlz3^2 | 1 | Cauchy-Green tensor, local coordinate system, 33 component | Domain 1 |  |
| solid.eel11 | 0.5*(-1+solid.Cel11) | 1 | Elastic strain tensor, local coordinate system, 11 component | Domain 1 |  |
| solid.eel12 | 0.5*solid.Cel12 | 1 | Elastic strain tensor, local coordinate system, 12 component | Domain 1 |  |
| solid.eel13 | 0.5*solid.Cel13 | 1 | Elastic strain tensor, local coordinate system, 13 component | Domain 1 |  |
| solid.eel22 | 0.5*(-1+solid.Cel22) | 1 | Elastic strain tensor, local coordinate system, 22 component | Domain 1 |  |
| solid.eel23 | 0.5*solid.Cel23 | 1 | Elastic strain tensor, local coordinate system, 23 component | Domain 1 |  |
| solid.eel33 | 0.5*(-1+solid.Cel33) | 1 | Elastic strain tensor, local coordinate system, 33 component | Domain 1 |  |
| solid.Felx1 | solid.Fdlx1*solid.Fiil11+solid.Fdlx2*solid.Fiil21+solid.Fdlx3*solid.Fiil31 | 1 | Elastic deformation gradient, local coordinate system, x1 component | Domain 1 |  |
| solid.Fely1 | solid.Fdly1*solid.Fiil11+solid.Fdly2*solid.Fiil21+solid.Fdly3*solid.Fiil31 | 1 | Elastic deformation gradient, local coordinate system, y1 component | Domain 1 |  |
| solid.Felz1 | solid.Fdlz1*solid.Fiil11+solid.Fdlz2*solid.Fiil21+solid.Fdlz3*solid.Fiil31 | 1 | Elastic deformation gradient, local coordinate system, z1 component | Domain 1 |  |
| solid.Felx2 | solid.Fdlx1*solid.Fiil12+solid.Fdlx2*solid.Fiil22+solid.Fdlx3*solid.Fiil32 | 1 | Elastic deformation gradient, local coordinate system, x2 component | Domain 1 |  |
| solid.Fely2 | solid.Fdly1*solid.Fiil12+solid.Fdly2*solid.Fiil22+solid.Fdly3*solid.Fiil32 | 1 | Elastic deformation gradient, local coordinate system, y2 component | Domain 1 |  |
| solid.Felz2 | solid.Fdlz1*solid.Fiil12+solid.Fdlz2*solid.Fiil22+solid.Fdlz3*solid.Fiil32 | 1 | Elastic deformation gradient, local coordinate system, z2 component | Domain 1 |  |
| solid.Felx3 | solid.Fdlx1*solid.Fiil13+solid.Fdlx2*solid.Fiil23+solid.Fdlx3*solid.Fiil33 | 1 | Elastic deformation gradient, local coordinate system, x3 component | Domain 1 |  |
| solid.Fely3 | solid.Fdly1*solid.Fiil13+solid.Fdly2*solid.Fiil23+solid.Fdly3*solid.Fiil33 | 1 | Elastic deformation gradient, local coordinate system, y3 component | Domain 1 |  |
| solid.Felz3 | solid.Fdlz1*solid.Fiil13+solid.Fdlz2*solid.Fiil23+solid.Fdlz3*solid.Fiil33 | 1 | Elastic deformation gradient, local coordinate system, z3 component | Domain 1 |  |
| solid.Cel11 | (solid.Fiil11*solid.Cl11+solid.Fiil21*solid.Cl12+solid.Fiil31*solid.Cl13)*solid.Fiil11+(solid.Fiil11*solid.Cl12+solid.Fiil21*solid.Cl22+solid.Fiil31*solid.Cl23)*solid.Fiil21+(solid.Fiil11*solid.Cl13+solid.Fiil21*solid.Cl23+solid.Fiil31*solid.Cl33)*solid.Fiil31 | 1 | Elastic Cauchy-Green tensor, local coordinate system, 11 component | Domain 1 |  |
| solid.Cel12 | (solid.Fiil11*solid.Cl11+solid.Fiil21*solid.Cl12+solid.Fiil31*solid.Cl13)*solid.Fiil12+(solid.Fiil11*solid.Cl12+solid.Fiil21*solid.Cl22+solid.Fiil31*solid.Cl23)*solid.Fiil22+(solid.Fiil11*solid.Cl13+solid.Fiil21*solid.Cl23+solid.Fiil31*solid.Cl33)*solid.Fiil32 | 1 | Elastic Cauchy-Green tensor, local coordinate system, 12 component | Domain 1 |  |
| solid.Cel13 | (solid.Fiil11*solid.Cl11+solid.Fiil21*solid.Cl12+solid.Fiil31*solid.Cl13)*solid.Fiil13+(solid.Fiil11*solid.Cl12+solid.Fiil21*solid.Cl22+solid.Fiil31*solid.Cl23)*solid.Fiil23+(solid.Fiil11*solid.Cl13+solid.Fiil21*solid.Cl23+solid.Fiil31*solid.Cl33)*solid.Fiil33 | 1 | Elastic Cauchy-Green tensor, local coordinate system, 13 component | Domain 1 |  |
| solid.Cel22 | (solid.Fiil12*solid.Cl11+solid.Fiil22*solid.Cl12+solid.Fiil32*solid.Cl13)*solid.Fiil12+(solid.Fiil12*solid.Cl12+solid.Fiil22*solid.Cl22+solid.Fiil32*solid.Cl23)*solid.Fiil22+(solid.Fiil12*solid.Cl13+solid.Fiil22*solid.Cl23+solid.Fiil32*solid.Cl33)*solid.Fiil32 | 1 | Elastic Cauchy-Green tensor, local coordinate system, 22 component | Domain 1 |  |
| solid.Cel23 | (solid.Fiil12*solid.Cl11+solid.Fiil22*solid.Cl12+solid.Fiil32*solid.Cl13)*solid.Fiil13+(solid.Fiil12*solid.Cl12+solid.Fiil22*solid.Cl22+solid.Fiil32*solid.Cl23)*solid.Fiil23+(solid.Fiil12*solid.Cl13+solid.Fiil22*solid.Cl23+solid.Fiil32*solid.Cl33)*solid.Fiil33 | 1 | Elastic Cauchy-Green tensor, local coordinate system, 23 component | Domain 1 |  |
| solid.Cel33 | (solid.Fiil13*solid.Cl11+solid.Fiil23*solid.Cl12+solid.Fiil33*solid.Cl13)*solid.Fiil13+(solid.Fiil13*solid.Cl12+solid.Fiil23*solid.Cl22+solid.Fiil33*solid.Cl23)*solid.Fiil23+(solid.Fiil13*solid.Cl13+solid.Fiil23*solid.Cl23+solid.Fiil33*solid.Cl33)*solid.Fiil33 | 1 | Elastic Cauchy-Green tensor, local coordinate system, 33 component | Domain 1 |  |
| solid.Fiil11 | 1 | 1 | Inelastic deformation gradient inverse, local coordinate system, 11 component | Domain 1 | Matrix multiplication |
| solid.Fiil21 | 0 | 1 | Inelastic deformation gradient inverse, local coordinate system, 21 component | Domain 1 | Matrix multiplication |
| solid.Fiil31 | 0 | 1 | Inelastic deformation gradient inverse, local coordinate system, 31 component | Domain 1 | Matrix multiplication |
| solid.Fiil12 | 0 | 1 | Inelastic deformation gradient inverse, local coordinate system, 12 component | Domain 1 | Matrix multiplication |
| solid.Fiil22 | 1 | 1 | Inelastic deformation gradient inverse, local coordinate system, 22 component | Domain 1 | Matrix multiplication |
| solid.Fiil32 | 0 | 1 | Inelastic deformation gradient inverse, local coordinate system, 32 component | Domain 1 | Matrix multiplication |
| solid.Fiil13 | 0 | 1 | Inelastic deformation gradient inverse, local coordinate system, 13 component | Domain 1 | Matrix multiplication |
| solid.Fiil23 | 0 | 1 | Inelastic deformation gradient inverse, local coordinate system, 23 component | Domain 1 | Matrix multiplication |
| solid.Fiil33 | 1 | 1 | Inelastic deformation gradient inverse, local coordinate system, 33 component | Domain 1 | Matrix multiplication |
| solid.Ji | 1/(solid.Fiil11*solid.Fiil22*solid.Fiil33+solid.Fiil12*solid.Fiil23*solid.Fiil31+solid.Fiil13*solid.Fiil21*solid.Fiil32-solid.Fiil11*solid.Fiil23*solid.Fiil32-solid.Fiil12*solid.Fiil21*solid.Fiil33-solid.Fiil13*solid.Fiil22*solid.Fiil31) | 1 | Inelastic volume ratio | Domain 1 |  |
| solid.Ceil11 | (solid.Cel22*solid.Cel33-solid.Cel23^2)/(solid.Cel11*solid.Cel22*solid.Cel33+2*solid.Cel12*solid.Cel23*solid.Cel13-solid.Cel11*solid.Cel23^2-solid.Cel12^2*solid.Cel33-solid.Cel22*solid.Cel13^2) | 1 | Elastic Cauchy-Green tensor inverse, local coordinate system, 11 component | Domain 1 |  |
| solid.Ceil12 | (solid.Cel23*solid.Cel13-solid.Cel12*solid.Cel33)/(solid.Cel11*solid.Cel22*solid.Cel33+2*solid.Cel12*solid.Cel23*solid.Cel13-solid.Cel11*solid.Cel23^2-solid.Cel12^2*solid.Cel33-solid.Cel22*solid.Cel13^2) | 1 | Elastic Cauchy-Green tensor inverse, local coordinate system, 12 component | Domain 1 |  |
| solid.Ceil13 | (solid.Cel12*solid.Cel23-solid.Cel22*solid.Cel13)/(solid.Cel11*solid.Cel22*solid.Cel33+2*solid.Cel12*solid.Cel23*solid.Cel13-solid.Cel11*solid.Cel23^2-solid.Cel12^2*solid.Cel33-solid.Cel22*solid.Cel13^2) | 1 | Elastic Cauchy-Green tensor inverse, local coordinate system, 13 component | Domain 1 |  |
| solid.Ceil22 | (solid.Cel11*solid.Cel33-solid.Cel13^2)/(solid.Cel11*solid.Cel22*solid.Cel33+2*solid.Cel12*solid.Cel23*solid.Cel13-solid.Cel11*solid.Cel23^2-solid.Cel12^2*solid.Cel33-solid.Cel22*solid.Cel13^2) | 1 | Elastic Cauchy-Green tensor inverse, local coordinate system, 22 component | Domain 1 |  |
| solid.Ceil23 | (solid.Cel12*solid.Cel13-solid.Cel11*solid.Cel23)/(solid.Cel11*solid.Cel22*solid.Cel33+2*solid.Cel12*solid.Cel23*solid.Cel13-solid.Cel11*solid.Cel23^2-solid.Cel12^2*solid.Cel33-solid.Cel22*solid.Cel13^2) | 1 | Elastic Cauchy-Green tensor inverse, local coordinate system, 23 component | Domain 1 |  |
| solid.Ceil33 | (solid.Cel11*solid.Cel22-solid.Cel12^2)/(solid.Cel11*solid.Cel22*solid.Cel33+2*solid.Cel12*solid.Cel23*solid.Cel13-solid.Cel11*solid.Cel23^2-solid.Cel12^2*solid.Cel33-solid.Cel22*solid.Cel13^2) | 1 | Elastic Cauchy-Green tensor inverse, local coordinate system, 33 component | Domain 1 |  |
| solid.Jel | solid.J/solid.Ji | 1 | Elastic volume ratio | Domain 1 |  |
| solid.mises | (3*solid.II2s)^0.5 | N/m² | von Mises stress | Domain 1 |  |
| solid.Sl11 | solid.Sliso11+solid.Slvol11 | N/m² | Second Piola-Kirchhoff stress, local coordinate system, 11 component | Domain 1 | + operation |
| solid.Sl12 | solid.Sliso12+solid.Slvol12 | N/m² | Second Piola-Kirchhoff stress, local coordinate system, 12 component | Domain 1 | + operation |
| solid.Sl13 | solid.Sliso13+solid.Slvol13 | N/m² | Second Piola-Kirchhoff stress, local coordinate system, 13 component | Domain 1 | + operation |
| solid.Sl22 | solid.Sliso22+solid.Slvol22 | N/m² | Second Piola-Kirchhoff stress, local coordinate system, 22 component | Domain 1 | + operation |
| solid.Sl23 | solid.Sliso23+solid.Slvol23 | N/m² | Second Piola-Kirchhoff stress, local coordinate system, 23 component | Domain 1 | + operation |
| solid.Sl33 | solid.Sliso33+solid.Slvol33 | N/m² | Second Piola-Kirchhoff stress, local coordinate system, 33 component | Domain 1 | + operation |
| solid.SXX | solid.Sl11 | N/m² | Second Piola-Kirchhoff stress, XX component | Domain 1 |  |
| solid.SXY | solid.Sl12 | N/m² | Second Piola-Kirchhoff stress, XY component | Domain 1 |  |
| solid.SXZ | solid.Sl13 | N/m² | Second Piola-Kirchhoff stress, XZ component | Domain 1 |  |
| solid.SYY | solid.Sl22 | N/m² | Second Piola-Kirchhoff stress, YY component | Domain 1 |  |
| solid.SYZ | solid.Sl23 | N/m² | Second Piola-Kirchhoff stress, YZ component | Domain 1 |  |
| solid.SZZ | solid.Sl33 | N/m² | Second Piola-Kirchhoff stress, ZZ component | Domain 1 |  |
| solid.u_ttX | d(u2TIME,TIME) | m/s² | Acceleration, X component | Domain 1 |  |
| solid.u_ttY | d(v2TIME,TIME) | m/s² | Acceleration, Y component | Domain 1 |  |
| solid.u_ttZ | d(w2TIME,TIME) | m/s² | Acceleration, Z component | Domain 1 |  |
| solid.afX | 0 | m/s² | Frame acceleration, X component | Domain 1 | + operation |
| solid.afY | 0 | m/s² | Frame acceleration, Y component | Domain 1 | + operation |
| solid.afZ | 0 | m/s² | Frame acceleration, Z component | Domain 1 | + operation |
| solid.accX | solid.u_ttX | m/s² | Total acceleration, X component | Domain 1 | + operation |
| solid.accY | solid.u_ttY | m/s² | Total acceleration, Y component | Domain 1 | + operation |
| solid.accZ | solid.u_ttZ | m/s² | Total acceleration, Z component | Domain 1 | + operation |
| solid.vel | sqrt(real(solid.u_tX)^2+real(solid.u_tY)^2+real(solid.u_tZ)^2) | m/s | Velocity magnitude | Domain 1 |  |
| solid.acc | sqrt(real(solid.accX)^2+real(solid.accY)^2+real(solid.accZ)^2) | m/s² | Total acceleration magnitude | Domain 1 |  |
| solid.u_tt | sqrt(real(solid.u_ttX)^2+real(solid.u_ttY)^2+real(solid.u_ttZ)^2) | m/s² | Acceleration magnitude | Domain 1 |  |
| solid.disp | sqrt(real(u2)^2+real(v2)^2+real(w2)^2) | m | Total displacement | Domain 1 |  |
| solid.sl11 | solid.sx | N/m² | Stress tensor, local coordinate system, 11 component | Domain 1 |  |
| solid.sl12 | solid.sxy | N/m² | Stress tensor, local coordinate system, 12 component | Domain 1 |  |
| solid.sl13 | solid.sxz | N/m² | Stress tensor, local coordinate system, 13 component | Domain 1 |  |
| solid.sl22 | solid.sy | N/m² | Stress tensor, local coordinate system, 22 component | Domain 1 |  |
| solid.sl23 | solid.syz | N/m² | Stress tensor, local coordinate system, 23 component | Domain 1 |  |
| solid.sl33 | solid.sz | N/m² | Stress tensor, local coordinate system, 33 component | Domain 1 |  |
| solid.eelvol | solid.eel11+solid.eel22+solid.eel33 | 1 | First invariant of elastic strain tensor | Domain 1 |  |
| solid.eeldev11 | solid.eel11-(solid.eel11+solid.eel22+solid.eel33)/3 | 1 | Deviatoric elastic strain tensor, local coordinate system, 11 component | Domain 1 |  |
| solid.eeldev12 | solid.eel12 | 1 | Deviatoric elastic strain tensor, local coordinate system, 12 component | Domain 1 |  |
| solid.eeldev13 | solid.eel13 | 1 | Deviatoric elastic strain tensor, local coordinate system, 13 component | Domain 1 |  |
| solid.eeldev22 | solid.eel22-(solid.eel11+solid.eel22+solid.eel33)/3 | 1 | Deviatoric elastic strain tensor, local coordinate system, 22 component | Domain 1 |  |
| solid.eeldev23 | solid.eel23 | 1 | Deviatoric elastic strain tensor, local coordinate system, 23 component | Domain 1 |  |
| solid.eeldev33 | solid.eel33-(solid.eel11+solid.eel22+solid.eel33)/3 | 1 | Deviatoric elastic strain tensor, local coordinate system, 33 component | Domain 1 |  |
| solid.II2eel | 0.5*((solid.eel11-(solid.eel11+solid.eel22+solid.eel33)/3)^2+2*solid.eel12^2+2*solid.eel13^2+(solid.eel22-(solid.eel11+solid.eel22+solid.eel33)/3)^2+2*solid.eel23^2+(solid.eel33-(solid.eel11+solid.eel22+solid.eel33)/3)^2) | 1 | Second invariant of deviatoric elastic strain tensor | Domain 1 |  |
| solid.Qh | 0 | W/m³ | Total power dissipation density | Domain 1 | + operation |
| solid.pwTXt | solid.pwTXTIME | kg/(m²·s³) | Tangential gradient of pw, X component, first time derivative | Boundaries 1–3, 6, 8, 12, 16 |  |
| solid.pwTYt | solid.pwTYTIME | kg/(m²·s³) | Tangential gradient of pw, Y component, first time derivative | Boundaries 1–3, 6, 8, 12, 16 |  |
| solid.pwTZt | solid.pwTZTIME | kg/(m²·s³) | Tangential gradient of pw, Z component, first time derivative | Boundaries 1–3, 6, 8, 12, 16 |  |
| solid.pwTXtt | solid.pwTXTIMETIME | kg/(m²·s⁴) | Tangential gradient of pw, X component, second time derivative | Boundaries 1–3, 6, 8, 12, 16 |  |
| solid.pwTYtt | solid.pwTYTIMETIME | kg/(m²·s⁴) | Tangential gradient of pw, Y component, second time derivative | Boundaries 1–3, 6, 8, 12, 16 |  |
| solid.pwTZtt | solid.pwTZTIMETIME | kg/(m²·s⁴) | Tangential gradient of pw, Z component, second time derivative | Boundaries 1–3, 6, 8, 12, 16 |  |
| solid.pwXt | solid.pwXTIME | kg/(m²·s³) | Gradient of pw, X component, first time derivative | Domain 1 |  |
| solid.pwYt | solid.pwYTIME | kg/(m²·s³) | Gradient of pw, Y component, first time derivative | Domain 1 |  |
| solid.pwZt | solid.pwZTIME | kg/(m²·s³) | Gradient of pw, Z component, first time derivative | Domain 1 |  |
| solid.pwXtt | solid.pwXTIMETIME | kg/(m²·s⁴) | Gradient of pw, X component, second time derivative | Domain 1 |  |
| solid.pwYtt | solid.pwYTIMETIME | kg/(m²·s⁴) | Gradient of pw, Y component, second time derivative | Domain 1 |  |
| solid.pwZtt | solid.pwZTIMETIME | kg/(m²·s⁴) | Gradient of pw, Z component, second time derivative | Domain 1 |  |
| solid.pwt | solid.pwTIME | W/m³ | Auxiliary pressure, first time derivative | Domain 1 |  |
| solid.pwt | solid.pwTIME | W/m³ | Auxiliary pressure, first time derivative | Boundaries 1–3, 6, 8, 12, 16 |  |
| solid.pwtt | solid.pwTIMETIME | Pa/s² | Auxiliary pressure, second time derivative | Domain 1 |  |
| solid.pwtt | solid.pwTIMETIME | Pa/s² | Auxiliary pressure, second time derivative | Boundaries 1–3, 6, 8, 12, 16 |  |
| solid.sysT11 | 1 | 1 | Transform to global system, 11 component | Domain 1 |  |
| solid.sysT21 | 0 | 1 | Transform to global system, 21 component | Domain 1 |  |
| solid.sysT31 | 0 | 1 | Transform to global system, 31 component | Domain 1 |  |
| solid.sysT12 | 0 | 1 | Transform to global system, 12 component | Domain 1 |  |
| solid.sysT22 | 1 | 1 | Transform to global system, 22 component | Domain 1 |  |
| solid.sysT32 | 0 | 1 | Transform to global system, 32 component | Domain 1 |  |
| solid.sysT13 | 0 | 1 | Transform to global system, 13 component | Domain 1 |  |
| solid.sysT23 | 0 | 1 | Transform to global system, 23 component | Domain 1 |  |
| solid.sysT33 | 1 | 1 | Transform to global system, 33 component | Domain 1 |  |
| solid.kappa | 55000 | Pa | Bulk modulus | Domain 1 |  |
| solid.Ws | solid.Wsvol+solid.Wsiso | J/m³ | Elastic strain energy density | Domain 1 | * operation |
| solid.Wsiso | solid.Ji*(solid.mup1*(-3+solid.stchIelp1^solid.alphap1+solid.stchIelp2^solid.alphap1+solid.stchIelp3^solid.alphap1)/solid.alphap1+solid.mup2*(-3+solid.stchIelp1^solid.alphap2+solid.stchIelp2^solid.alphap2+solid.stchIelp3^solid.alphap2)/solid.alphap2) | J/m³ | Isochoric strain energy density | Domain 1 | * operation |
| solid.Wsvol | -solid.pw*solid.Ji*(-1+solid.Jel) | J/m³ | Volumetric strain energy density | Domain 1 | * operation |
| solid.Uvol | 0.5*solid.Ji*solid.kappa*(-1+solid.Jel)^2 | J/m³ | Auxiliary volumetric energy density | Domain 1 |  |
| solid.Sliso11 | 2*d(solid.Wsiso,solid.Cl11) | N/m² | Second Piola-Kirchhoff isochoric stress local coordinate system, 11 component | Domain 1 | + operation |
| solid.Sliso12 | d(solid.Wsiso,solid.Cl12) | N/m² | Second Piola-Kirchhoff isochoric stress local coordinate system, 12 component | Domain 1 | + operation |
| solid.Sliso13 | d(solid.Wsiso,solid.Cl13) | N/m² | Second Piola-Kirchhoff isochoric stress local coordinate system, 13 component | Domain 1 | + operation |
| solid.Sliso22 | 2*d(solid.Wsiso,solid.Cl22) | N/m² | Second Piola-Kirchhoff isochoric stress local coordinate system, 22 component | Domain 1 | + operation |
| solid.Sliso23 | d(solid.Wsiso,solid.Cl23) | N/m² | Second Piola-Kirchhoff isochoric stress local coordinate system, 23 component | Domain 1 | + operation |
| solid.Sliso33 | 2*d(solid.Wsiso,solid.Cl33) | N/m² | Second Piola-Kirchhoff isochoric stress local coordinate system, 33 component | Domain 1 | + operation |
| solid.Slvol11 | -solid.pw*solid.J*(solid.Cl22*solid.Cl33-solid.Cl23^2)/(solid.Cl11*solid.Cl22*solid.Cl33+2*solid.Cl12*solid.Cl23*solid.Cl13-solid.Cl11*solid.Cl23^2-solid.Cl12^2*solid.Cl33-solid.Cl22*solid.Cl13^2) | N/m² | Second Piola-Kirchhoff volumetric stress local coordinate system, 11 component | Domain 1 | * operation |
| solid.Slvol12 | -solid.pw*solid.J*(solid.Cl23*solid.Cl13-solid.Cl12*solid.Cl33)/(solid.Cl11*solid.Cl22*solid.Cl33+2*solid.Cl12*solid.Cl23*solid.Cl13-solid.Cl11*solid.Cl23^2-solid.Cl12^2*solid.Cl33-solid.Cl22*solid.Cl13^2) | N/m² | Second Piola-Kirchhoff volumetric stress local coordinate system, 12 component | Domain 1 | * operation |
| solid.Slvol13 | -solid.pw*solid.J*(solid.Cl12*solid.Cl23-solid.Cl22*solid.Cl13)/(solid.Cl11*solid.Cl22*solid.Cl33+2*solid.Cl12*solid.Cl23*solid.Cl13-solid.Cl11*solid.Cl23^2-solid.Cl12^2*solid.Cl33-solid.Cl22*solid.Cl13^2) | N/m² | Second Piola-Kirchhoff volumetric stress local coordinate system, 13 component | Domain 1 | * operation |
| solid.Slvol22 | -solid.pw*solid.J*(solid.Cl11*solid.Cl33-solid.Cl13^2)/(solid.Cl11*solid.Cl22*solid.Cl33+2*solid.Cl12*solid.Cl23*solid.Cl13-solid.Cl11*solid.Cl23^2-solid.Cl12^2*solid.Cl33-solid.Cl22*solid.Cl13^2) | N/m² | Second Piola-Kirchhoff volumetric stress local coordinate system, 22 component | Domain 1 | * operation |
| solid.Slvol23 | -solid.pw*solid.J*(solid.Cl12*solid.Cl13-solid.Cl11*solid.Cl23)/(solid.Cl11*solid.Cl22*solid.Cl33+2*solid.Cl12*solid.Cl23*solid.Cl13-solid.Cl11*solid.Cl23^2-solid.Cl12^2*solid.Cl33-solid.Cl22*solid.Cl13^2) | N/m² | Second Piola-Kirchhoff volumetric stress local coordinate system, 23 component | Domain 1 | * operation |
| solid.Slvol33 | -solid.pw*solid.J*(solid.Cl11*solid.Cl22-solid.Cl12^2)/(solid.Cl11*solid.Cl22*solid.Cl33+2*solid.Cl12*solid.Cl23*solid.Cl13-solid.Cl11*solid.Cl23^2-solid.Cl12^2*solid.Cl33-solid.Cl22*solid.Cl13^2) | N/m² | Second Piola-Kirchhoff volumetric stress local coordinate system, 33 component | Domain 1 | * operation |
| solid.pm | -d(solid.Uvol,solid.J) | N/m² | Pressure | Domain 1 |  |
| solid.Wh | solid.Ws | J/m³ | Stored energy density | Domain 1 | + operation |
| solid.I1Cel | solid.Cel11+solid.Cel22+solid.Cel33 | 1 | First invariant of elastic right Cauchy-Green tensor | Domain 1 |  |
| solid.I2Cel | 0.5*(solid.I1Cel^2-solid.Cel11^2-2*solid.Cel12^2-2*solid.Cel13^2-solid.Cel22^2-2*solid.Cel23^2-solid.Cel33^2) | 1 | Second invariant of elastic right Cauchy-Green tensor | Domain 1 |  |
| solid.I3Cel | solid.Cel11*solid.Cel22*solid.Cel33+2*solid.Cel12*solid.Cel23*solid.Cel13-solid.Cel11*solid.Cel23^2-solid.Cel12^2*solid.Cel33-solid.Cel22*solid.Cel13^2 | 1 | Third invariant of elastic right Cauchy-Green tensor | Domain 1 |  |
| solid.CIel11 | solid.Jel^(-2/3)*solid.Cel11 | 1 | Isochoric elastic Cauchy-Green tensor, local coordinate system, 11 component | Domain 1 |  |
| solid.CIel12 | solid.Jel^(-2/3)*solid.Cel12 | 1 | Isochoric elastic Cauchy-Green tensor, local coordinate system, 12 component | Domain 1 |  |
| solid.CIel13 | solid.Jel^(-2/3)*solid.Cel13 | 1 | Isochoric elastic Cauchy-Green tensor, local coordinate system, 13 component | Domain 1 |  |
| solid.CIel22 | solid.Jel^(-2/3)*solid.Cel22 | 1 | Isochoric elastic Cauchy-Green tensor, local coordinate system, 22 component | Domain 1 |  |
| solid.CIel23 | solid.Jel^(-2/3)*solid.Cel23 | 1 | Isochoric elastic Cauchy-Green tensor, local coordinate system, 23 component | Domain 1 |  |
| solid.CIel33 | solid.Jel^(-2/3)*solid.Cel33 | 1 | Isochoric elastic Cauchy-Green tensor, local coordinate system, 33 component | Domain 1 |  |
| solid.I1CIel | solid.Jel^(-2/3)*solid.I1Cel | 1 | First invariant of isochoric elastic right Cauchy-Green tensor | Domain 1 |  |
| solid.I2CIel | solid.Jel^(-4/3)*solid.I2Cel | 1 | Second invariant of isochoric elastic right Cauchy-Green tensor | Domain 1 |  |
| solid.I1eel | 0.5*(-3+solid.I1Cel) | 1 | First invariant of elastic Green-Lagrange strain tensor | Domain 1 |  |
| solid.I2eel | 0.25*(3+solid.I2Cel-2*solid.I1Cel) | 1 | Second invariant of elastic Green-Lagrange strain tensor | Domain 1 |  |
| solid.I3eel | 0.125*(-1+solid.I3Cel-solid.I2Cel+solid.I1Cel) | 1 | Third invariant of elastic Green-Lagrange strain tensor | Domain 1 |  |
| solid.I1eIel | 0.5*(-3+solid.I1CIel) | 1 | First invariant of isochoric elastic Green-Lagrange strain tensor | Domain 1 |  |
| solid.I2eIel | 0.25*(3+solid.I2CIel-2*solid.I1CIel) | 1 | Second invariant of isochoric elastic Green-Lagrange strain tensor | Domain 1 |  |
| solid.I3eIel | 0.125*(solid.I1CIel-solid.I2CIel) | 1 | Third invariant of isochoric elastic Green-Lagrange strain tensor | Domain 1 |  |
| solid.stchIelp1 | solid.stchelp1/solid.Jel^(1/3) | 1 | First principal isochoric elastic stretch | Domain 1 |  |
| solid.stchIelp2 | solid.stchelp2/solid.Jel^(1/3) | 1 | Second principal isochoric elastic stretch | Domain 1 |  |
| solid.stchIelp3 | solid.stchelp3/solid.Jel^(1/3) | 1 | Third principal isochoric elastic stretch | Domain 1 |  |
| solid.Wk | 0.5*(u2TIME^2+v2TIME^2+w2TIME^2)*solid.rho | J/m³ | Kinetic energy density | Domain 1 | + operation |
| solid.Ws_tot | root.comp1.solid.hmm2.int14(solid.Ws) | J | Total elastic strain energy | Global | + operation |
| solid.Wh_tot | root.comp1.solid.hmm2.int15(solid.Ws) | J | Total stored energy | Global | + operation |
| solid.PxX | solid.FdxX*solid.SX+solid.FdxY*solid.SXY+solid.FdxZ*solid.SXZ | N/m² | First Piola-Kirchhoff stress, xX component | Domain 1 |  |
| solid.PyX | solid.FdyX*solid.SX+solid.FdyY*solid.SXY+solid.FdyZ*solid.SXZ | N/m² | First Piola-Kirchhoff stress, yX component | Domain 1 |  |
| solid.PzX | solid.FdzX*solid.SX+solid.FdzY*solid.SXY+solid.FdzZ*solid.SXZ | N/m² | First Piola-Kirchhoff stress, zX component | Domain 1 |  |
| solid.PxY | solid.FdxX*solid.SXY+solid.FdxY*solid.SY+solid.FdxZ*solid.SYZ | N/m² | First Piola-Kirchhoff stress, xY component | Domain 1 |  |
| solid.PyY | solid.FdyX*solid.SXY+solid.FdyY*solid.SY+solid.FdyZ*solid.SYZ | N/m² | First Piola-Kirchhoff stress, yY component | Domain 1 |  |
| solid.PzY | solid.FdzX*solid.SXY+solid.FdzY*solid.SY+solid.FdzZ*solid.SYZ | N/m² | First Piola-Kirchhoff stress, zY component | Domain 1 |  |
| solid.PxZ | solid.FdxX*solid.SXZ+solid.FdxY*solid.SYZ+solid.FdxZ*solid.SZ | N/m² | First Piola-Kirchhoff stress, xZ component | Domain 1 |  |
| solid.PyZ | solid.FdyX*solid.SXZ+solid.FdyY*solid.SYZ+solid.FdyZ*solid.SZ | N/m² | First Piola-Kirchhoff stress, yZ component | Domain 1 |  |
| solid.PzZ | solid.FdzX*solid.SXZ+solid.FdzY*solid.SYZ+solid.FdzZ*solid.SZ | N/m² | First Piola-Kirchhoff stress, zZ component | Domain 1 |  |
| solid.sx | ((solid.Fdlx1*solid.Sl11+solid.Fdlx2*solid.Sl12+solid.Fdlx3*solid.Sl13)*solid.Fdlx1+(solid.Fdlx1*solid.Sl12+solid.Fdlx2*solid.Sl22+solid.Fdlx3*solid.Sl23)*solid.Fdlx2+(solid.Fdlx1*solid.Sl13+solid.Fdlx2*solid.Sl23+solid.Fdlx3*solid.Sl33)*solid.Fdlx3)/solid.J | N/m² | Stress tensor, x component | Domain 1 |  |
| solid.sxy | ((solid.Fdlx1*solid.Sl11+solid.Fdlx2*solid.Sl12+solid.Fdlx3*solid.Sl13)*solid.Fdly1+(solid.Fdlx1*solid.Sl12+solid.Fdlx2*solid.Sl22+solid.Fdlx3*solid.Sl23)*solid.Fdly2+(solid.Fdlx1*solid.Sl13+solid.Fdlx2*solid.Sl23+solid.Fdlx3*solid.Sl33)*solid.Fdly3)/solid.J | N/m² | Stress tensor, xy component | Domain 1 |  |
| solid.sxz | ((solid.Fdlx1*solid.Sl11+solid.Fdlx2*solid.Sl12+solid.Fdlx3*solid.Sl13)*solid.Fdlz1+(solid.Fdlx1*solid.Sl12+solid.Fdlx2*solid.Sl22+solid.Fdlx3*solid.Sl23)*solid.Fdlz2+(solid.Fdlx1*solid.Sl13+solid.Fdlx2*solid.Sl23+solid.Fdlx3*solid.Sl33)*solid.Fdlz3)/solid.J | N/m² | Stress tensor, xz component | Domain 1 |  |
| solid.sy | ((solid.Fdly1*solid.Sl11+solid.Fdly2*solid.Sl12+solid.Fdly3*solid.Sl13)*solid.Fdly1+(solid.Fdly1*solid.Sl12+solid.Fdly2*solid.Sl22+solid.Fdly3*solid.Sl23)*solid.Fdly2+(solid.Fdly1*solid.Sl13+solid.Fdly2*solid.Sl23+solid.Fdly3*solid.Sl33)*solid.Fdly3)/solid.J | N/m² | Stress tensor, y component | Domain 1 |  |
| solid.syz | ((solid.Fdly1*solid.Sl11+solid.Fdly2*solid.Sl12+solid.Fdly3*solid.Sl13)*solid.Fdlz1+(solid.Fdly1*solid.Sl12+solid.Fdly2*solid.Sl22+solid.Fdly3*solid.Sl23)*solid.Fdlz2+(solid.Fdly1*solid.Sl13+solid.Fdly2*solid.Sl23+solid.Fdly3*solid.Sl33)*solid.Fdlz3)/solid.J | N/m² | Stress tensor, yz component | Domain 1 |  |
| solid.sz | ((solid.Fdlz1*solid.Sl11+solid.Fdlz2*solid.Sl12+solid.Fdlz3*solid.Sl13)*solid.Fdlz1+(solid.Fdlz1*solid.Sl12+solid.Fdlz2*solid.Sl22+solid.Fdlz3*solid.Sl23)*solid.Fdlz2+(solid.Fdlz1*solid.Sl13+solid.Fdlz2*solid.Sl23+solid.Fdlz3*solid.Sl33)*solid.Fdlz3)/solid.J | N/m² | Stress tensor, z component | Domain 1 |  |
| solid.sdevx | solid.sx-(solid.sx+solid.sy+solid.sz)/3 | N/m² | Deviatoric stress tensor, x component | Domain 1 |  |
| solid.sdevxy | solid.sxy | N/m² | Deviatoric stress tensor, xy component | Domain 1 |  |
| solid.sdevxz | solid.sxz | N/m² | Deviatoric stress tensor, xz component | Domain 1 |  |
| solid.sdevy | solid.sy-(solid.sx+solid.sy+solid.sz)/3 | N/m² | Deviatoric stress tensor, y component | Domain 1 |  |
| solid.sdevyz | solid.syz | N/m² | Deviatoric stress tensor, yz component | Domain 1 |  |
| solid.sdevz | solid.sz-(solid.sx+solid.sy+solid.sz)/3 | N/m² | Deviatoric stress tensor, z component | Domain 1 |  |
| solid.SdevX | solid.SX-(solid.SX+solid.SY+solid.SZ)/3 | N/m² | Deviatoric second Piola-Kirchhoff stress, X component | Domain 1 |  |
| solid.SdevXY | solid.SXY | N/m² | Deviatoric second Piola-Kirchhoff stress, XY component | Domain 1 |  |
| solid.SdevXZ | solid.SXZ | N/m² | Deviatoric second Piola-Kirchhoff stress, XZ component | Domain 1 |  |
| solid.SdevY | solid.SY-(solid.SX+solid.SY+solid.SZ)/3 | N/m² | Deviatoric second Piola-Kirchhoff stress, Y component | Domain 1 |  |
| solid.SdevYZ | solid.SYZ | N/m² | Deviatoric second Piola-Kirchhoff stress, YZ component | Domain 1 |  |
| solid.SdevZ | solid.SZ-(solid.SX+solid.SY+solid.SZ)/3 | N/m² | Deviatoric second Piola-Kirchhoff stress, Z component | Domain 1 |  |
| solid.I1s | solid.sl11+solid.sl22+solid.sl33 | N/m² | First principal invariant of stress | Domain 1 |  |
| solid.I2s | 0.5*(solid.I1s^2-solid.sl11^2-2*solid.sl12^2-2*solid.sl13^2-solid.sl22^2-2*solid.sl23^2-solid.sl33^2) | kg²/(m²·s⁴) | Second principal invariant of stress | Domain 1 |  |
| solid.I3s | solid.sl11*solid.sl22*solid.sl33+2*solid.sl12*solid.sl23*solid.sl13-solid.sl11*solid.sl23^2-solid.sl12^2*solid.sl33-solid.sl22*solid.sl13^2 | kg³/(m³·s⁶) | Third principal invariant of stress | Domain 1 |  |
| solid.II2s | 0.5*((solid.sl11-(solid.sl11+solid.sl22+solid.sl33)/3)^2+2*solid.sl12^2+2*solid.sl13^2+(solid.sl22-(solid.sl11+solid.sl22+solid.sl33)/3)^2+2*solid.sl23^2+(solid.sl33-(solid.sl11+solid.sl22+solid.sl33)/3)^2) | kg²/(m²·s⁴) | Second invariant of stress deviator | Domain 1 |  |
| solid.II3s | (solid.sl11-(solid.sl11+solid.sl22+solid.sl33)/3)*(solid.sl22-(solid.sl11+solid.sl22+solid.sl33)/3)*(solid.sl33-(solid.sl11+solid.sl22+solid.sl33)/3)+2*solid.sl12*solid.sl23*solid.sl13-(solid.sl11-(solid.sl11+solid.sl22+solid.sl33)/3)*solid.sl23^2-solid.sl12^2*(solid.sl33-(solid.sl11+solid.sl22+solid.sl33)/3)-(solid.sl22-(solid.sl11+solid.sl22+solid.sl33)/3)*solid.sl13^2 | kg³/(m³·s⁶) | Third invariant of stress deviator | Domain 1 |  |
| solid.thetaL | atan2(sqrt(max(0.14814814814814814*solid.II2s^3-solid.II3s^2,eps)),solid.II3s)/3 | rad | Lode angle | Domain 1 |  |
| solid.curlUX | solid.gradUzY-solid.gradUyZ | 1 | Curl of displacement, X component | Domain 1 |  |
| solid.curlUY | solid.gradUxZ-solid.gradUzX | 1 | Curl of displacement, Y component | Domain 1 |  |
| solid.curlUZ | solid.gradUyX-solid.gradUxY | 1 | Curl of displacement, Z component | Domain 1 |  |
| solid.tresca | solid.sp1-solid.sp3 | N/m² | Tresca stress | Domain 1 |  |
| solid.RFx | reacf(u2) | N | Reaction force, x component | Domain 1 |  |
| solid.RFy | reacf(v2) | N | Reaction force, y component | Domain 1 |  |
| solid.RFz | reacf(w2) | N | Reaction force, z component | Domain 1 |  |
| solid.RMx | solid.RFz*(y-solid.refpnty)-solid.RFy*(z-solid.refpntz) | N·m | Reaction moment, x component | Domain 1 |  |
| solid.RMy | -solid.RFz*(x-solid.refpntx)+solid.RFx*(z-solid.refpntz) | N·m | Reaction moment, y component | Domain 1 |  |
| solid.RMz | solid.RFy*(x-solid.refpntx)-solid.RFx*(y-solid.refpnty) | N·m | Reaction moment, z component | Domain 1 |  |
| solid.Tax | solid.sx*solid.nx+solid.sxy*solid.ny+solid.sxz*solid.nz | N/m² | Traction (force/area), x component | Boundaries 1–3, 6, 8, 12, 16 |  |
| solid.Tay | solid.sxy*solid.nx+solid.sy*solid.ny+solid.syz*solid.nz | N/m² | Traction (force/area), y component | Boundaries 1–3, 6, 8, 12, 16 |  |
| solid.Taz | solid.sxz*solid.nx+solid.syz*solid.ny+solid.sz*solid.nz | N/m² | Traction (force/area), z component | Boundaries 1–3, 6, 8, 12, 16 |  |
| solid.stn | (solid.sx*solid.nx+solid.sxy*solid.ny+solid.sxz*solid.nz)*solid.nx+(solid.sxy*solid.nx+solid.sy*solid.ny+solid.syz*solid.nz)*solid.ny+(solid.sxz*solid.nx+solid.syz*solid.ny+solid.sz*solid.nz)*solid.nz | N/m² | Normal stress | Boundaries 1–3, 6, 8, 12, 16 |  |
| solid.Tanx | ((solid.sx*solid.nx+solid.sxy*solid.ny+solid.sxz*solid.nz)*solid.nx+(solid.sxy*solid.nx+solid.sy*solid.ny+solid.syz*solid.nz)*solid.ny+(solid.sxz*solid.nx+solid.syz*solid.ny+solid.sz*solid.nz)*solid.nz)*solid.nx | N/m² | Normal component of traction, x component | Boundaries 1–3, 6, 8, 12, 16 |  |
| solid.Tany | ((solid.sx*solid.nx+solid.sxy*solid.ny+solid.sxz*solid.nz)*solid.nx+(solid.sxy*solid.nx+solid.sy*solid.ny+solid.syz*solid.nz)*solid.ny+(solid.sxz*solid.nx+solid.syz*solid.ny+solid.sz*solid.nz)*solid.nz)*solid.ny | N/m² | Normal component of traction, y component | Boundaries 1–3, 6, 8, 12, 16 |  |
| solid.Tanz | ((solid.sx*solid.nx+solid.sxy*solid.ny+solid.sxz*solid.nz)*solid.nx+(solid.sxy*solid.nx+solid.sy*solid.ny+solid.syz*solid.nz)*solid.ny+(solid.sxz*solid.nx+solid.syz*solid.ny+solid.sz*solid.nz)*solid.nz)*solid.nz | N/m² | Normal component of traction, z component | Boundaries 1–3, 6, 8, 12, 16 |  |
| solid.Tatx | solid.sx*solid.nx+solid.sxy*solid.ny+solid.sxz*solid.nz-((solid.sx*solid.nx+solid.sxy*solid.ny+solid.sxz*solid.nz)*solid.nx+(solid.sxy*solid.nx+solid.sy*solid.ny+solid.syz*solid.nz)*solid.ny+(solid.sxz*solid.nx+solid.syz*solid.ny+solid.sz*solid.nz)*solid.nz)*solid.nx | N/m² | Shear component of traction, x component | Boundaries 1–3, 6, 8, 12, 16 |  |
| solid.Taty | solid.sxy*solid.nx+solid.sy*solid.ny+solid.syz*solid.nz-((solid.sx*solid.nx+solid.sxy*solid.ny+solid.sxz*solid.nz)*solid.nx+(solid.sxy*solid.nx+solid.sy*solid.ny+solid.syz*solid.nz)*solid.ny+(solid.sxz*solid.nx+solid.syz*solid.ny+solid.sz*solid.nz)*solid.nz)*solid.ny | N/m² | Shear component of traction, y component | Boundaries 1–3, 6, 8, 12, 16 |  |
| solid.Tatz | solid.sxz*solid.nx+solid.syz*solid.ny+solid.sz*solid.nz-((solid.sx*solid.nx+solid.sxy*solid.ny+solid.sxz*solid.nz)*solid.nx+(solid.sxy*solid.nx+solid.sy*solid.ny+solid.syz*solid.nz)*solid.ny+(solid.sxz*solid.nx+solid.syz*solid.ny+solid.sz*solid.nz)*solid.nz)*solid.nz | N/m² | Shear component of traction, z component | Boundaries 1–3, 6, 8, 12, 16 |  |
| solid.stt | sqrt((solid.sx*solid.nx+solid.sxy*solid.ny+solid.sxz*solid.nz-((solid.sx*solid.nx+solid.sxy*solid.ny+solid.sxz*solid.nz)*solid.nx+(solid.sxy*solid.nx+solid.sy*solid.ny+solid.syz*solid.nz)*solid.ny+(solid.sxz*solid.nx+solid.syz*solid.ny+solid.sz*solid.nz)*solid.nz)*solid.nx)^2+(solid.sxy*solid.nx+solid.sy*solid.ny+solid.syz*solid.nz-((solid.sx*solid.nx+solid.sxy*solid.ny+solid.sxz*solid.nz)*solid.nx+(solid.sxy*solid.nx+solid.sy*solid.ny+solid.syz*solid.nz)*solid.ny+(solid.sxz*solid.nx+solid.syz*solid.ny+solid.sz*solid.nz)*solid.nz)*solid.ny)^2+(solid.sxz*solid.nx+solid.syz*solid.ny+solid.sz*solid.nz-((solid.sx*solid.nx+solid.sxy*solid.ny+solid.sxz*solid.nz)*solid.nx+(solid.sxy*solid.nx+solid.sy*solid.ny+solid.syz*solid.nz)*solid.ny+(solid.sxz*solid.nx+solid.syz*solid.ny+solid.sz*solid.nz)*solid.nz)*solid.nz)^2) | N/m² | Shear stress | Boundaries 1–3, 6, 8, 12, 16 |  |
| solid.Qh_tot | root.comp1.solid.hmm2.int16(solid.Qh) | W | Total power dissipation | Global | + operation |
| solid.IX | -solid.SX*solid.u_tX-solid.SXY*solid.u_tY-solid.SXZ*solid.u_tZ | W/m² | Mechanical energy flux, X component | Domain 1 |  |
| solid.IY | -solid.SXY*solid.u_tX-solid.SY*solid.u_tY-solid.SYZ*solid.u_tZ | W/m² | Mechanical energy flux, Y component | Domain 1 |  |
| solid.IZ | -solid.SXZ*solid.u_tX-solid.SYZ*solid.u_tY-solid.SZ*solid.u_tZ | W/m² | Mechanical energy flux, Z component | Domain 1 |  |
| solid.nI | nX*solid.IX+nY*solid.IY+nZ*solid.IZ | W/m² | Outward mechanical energy flux | Boundaries 1–3, 8, 12, 16 | Meta |
| solid.hmm2.nI | nX*solid.IX+nY*solid.IY+nZ*solid.IZ | W/m² | Outward mechanical energy flux | Boundaries 1–3, 6, 8, 12, 16 | Meta |

#### Shape functions

| **Name** | **Shape function** | **Unit** | **Description** | **Shape frame** | **Selection** | **Details** |
| --- | --- | --- | --- | --- | --- | --- |
| u2 | Lagrange (Quadratic) | m | Displacement field, X component | Material | Domain 1 |  |
| v2 | Lagrange (Quadratic) | m | Displacement field, Y component | Material | Domain 1 |  |
| w2 | Lagrange (Quadratic) | m | Displacement field, Z component | Material | Domain 1 |  |
| solid.pw | Lagrange (Linear) | N/m² | Auxiliary pressure | Material | Domain 1 |  |
| solid.pw | Lagrange (Linear) | N/m² | Auxiliary pressure | Material | Boundaries 1–3, 6, 8, 12, 16 | Slit |

#### Weak expressions

| **Weak expression** | **Integration order** | **Integration frame** | **Selection** |
| --- | --- | --- | --- |
| (solid.pw-solid.pm)*test(solid.pw)/solid.K | 4 | Material | Domain 1 |
| -solid.Sl11*test(solid.el11)-2*solid.Sl12*test(solid.el12)-2*solid.Sl13*test(solid.el13)-solid.Sl22*test(solid.el22)-2*solid.Sl23*test(solid.el23)-solid.Sl33*test(solid.el33) | 4 | Material | Domain 1 |
| solid.rho*(-d(u2TIME,TIME)*test(u2)-d(v2TIME,TIME)*test(v2)-d(w2TIME,TIME)*test(w2)) | 4 | Material | Domain 1 |

- - 1. Fixed Constraint 1


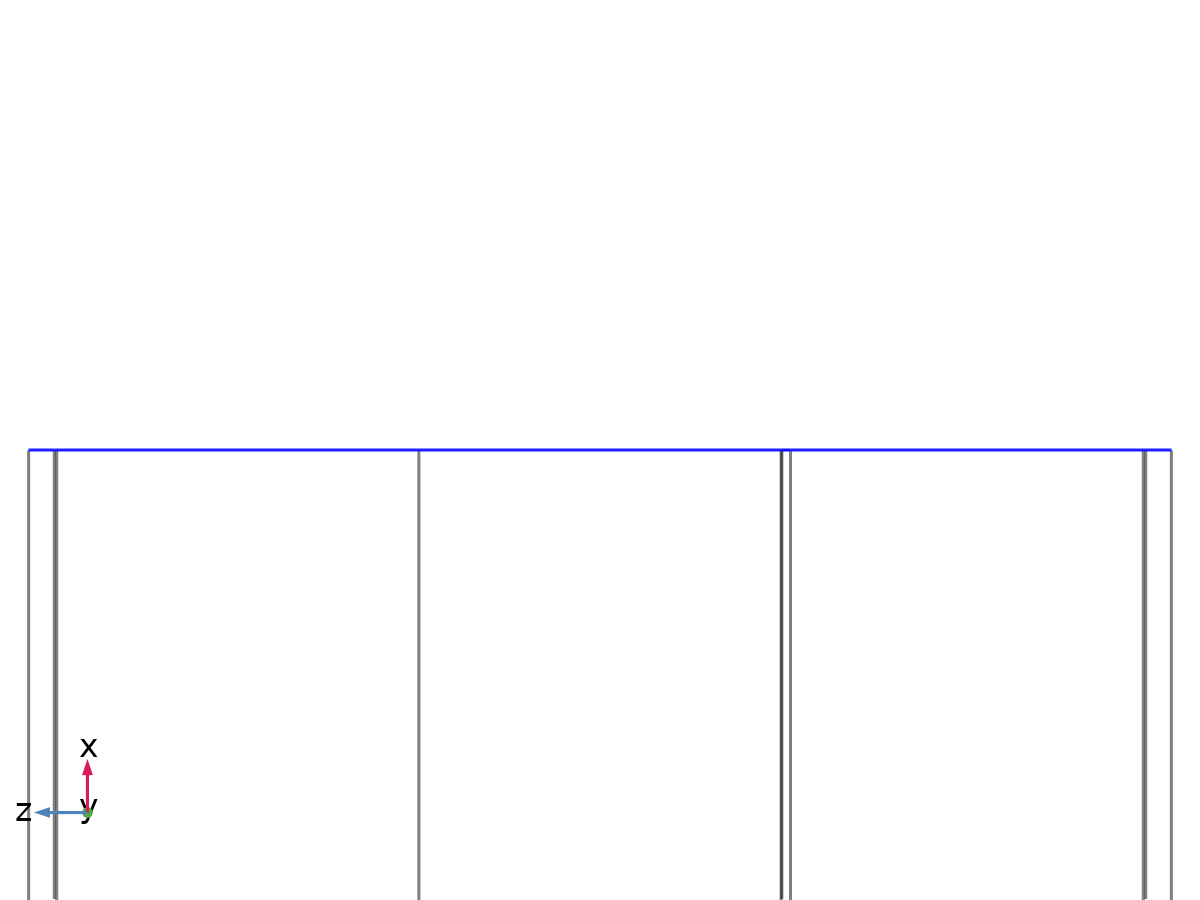


Fixed Constraint 1

Selection

| Geometric entity level | Boundary |
| --- | --- |
| Selection | Geometry geom1: Dimension 2: Boundaries 16–17 |

Equations


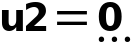


Used products

| COMSOL Multiphysics |
| --- |

#### Variables

| **Name** | **Expression** | **Unit** | **Description** | **Selection** | **Details** |
| --- | --- | --- | --- | --- | --- |
| solid.fix1.usx | u2 | m | Displacement, spatial coordinate system, x component | Boundaries 16–17 | + operation |
| solid.fix1.usy | v2 | m | Displacement, spatial coordinate system, y component | Boundaries 16–17 | + operation |
| solid.fix1.usz | w2 | m | Displacement, spatial coordinate system, z component | Boundaries 16–17 | + operation |

#### Constraints

| **Constraint** | **Constraint force** | **Shape function** | **Selection** | **Details** |
| --- | --- | --- | --- | --- |
| -solid.fix1.usx | test(-solid.fix1.usx) | Lagrange (Quadratic) | Boundaries 16–17 | Nodal |
| -solid.fix1.usy | test(-solid.fix1.usy) | Lagrange (Quadratic) | Boundaries 16–17 | Nodal |
| -solid.fix1.usz | test(-solid.fix1.usz) | Lagrange (Quadratic) | Boundaries 16–17 | Nodal |

- 1. Multiphysics
     1. Fluid-Structure Interaction 1

Used products

| COMSOL Multiphysics |
| --- |
| Structural Mechanics Module |


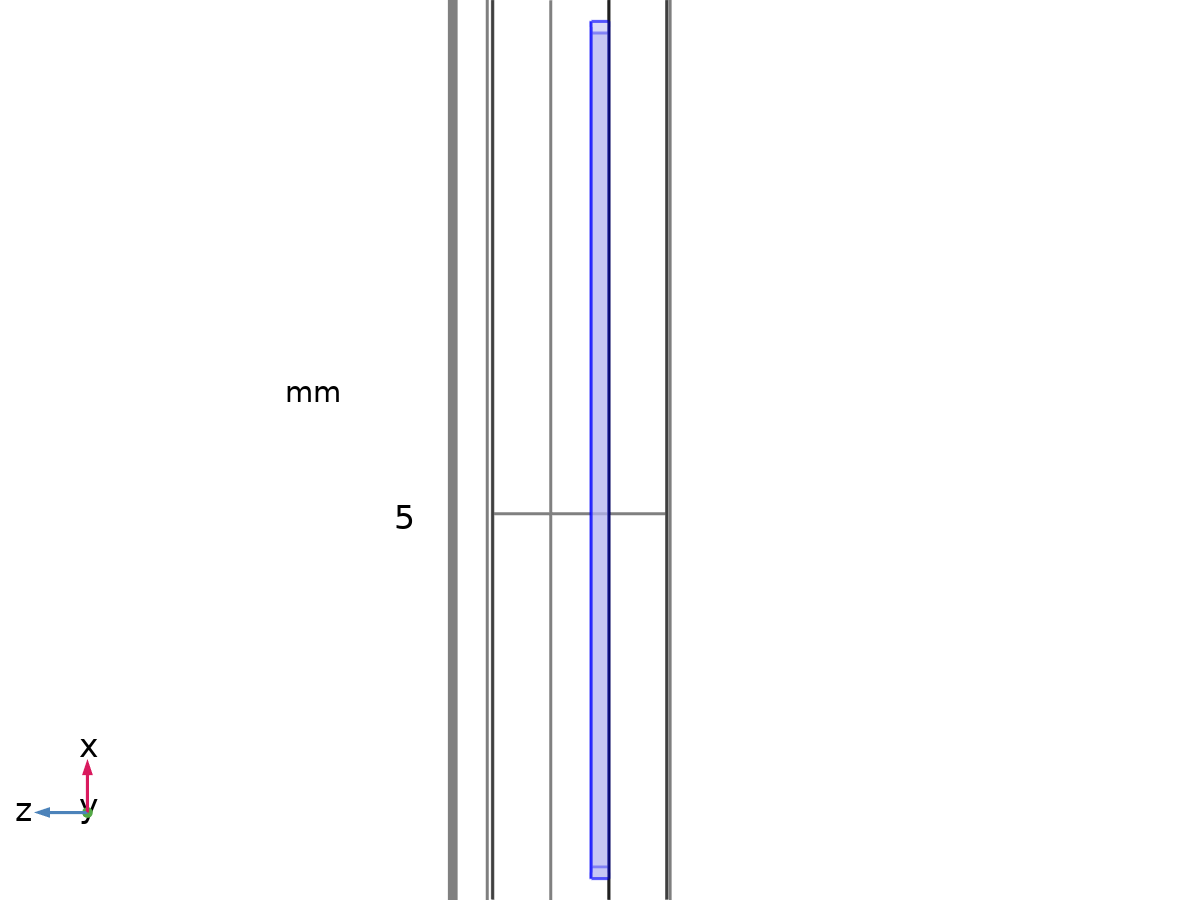


Fluid-Structure Interaction 1

Selection

| Geometric entity level | Boundary |
| --- | --- |
| Selection | Geometry geom1: Dimension 2: Boundaries 10–14 |

#### Fixed geometry

Settings

| **Description** | **Value** |
| --- | --- |
| Fixed geometry coupling type | Fully coupled |

#### Coupled interfaces

Settings

| **Description** | **Value** |
| --- | --- |
| Fluid | Laminar Flow (spf) |
| Structure | Solid Mechanics (solid) |

#### Variables

| **Name** | **Expression** | **Unit** | **Description** | **Selection** | **Details** |
| --- | --- | --- | --- | --- | --- |
| spf.usePseudoTimeStepping | 1 | 1 | Help variable | Global | + operation |
| fsi1.u_fluidx | u | m/s | Velocity field, x component | Boundaries 10–14 |  |
| fsi1.u_fluidy | v | m/s | Velocity field, y component | Boundaries 10–14 |  |
| fsi1.u_fluidz | w | m/s | Velocity field, z component | Boundaries 10–14 |  |
| fsi1.u_solid1 | u2 | m | Displacement, 1 component | Boundaries 10–14 |  |
| fsi1.u_solid2 | v2 | m | Displacement, 2 component | Boundaries 10–14 |  |
| fsi1.u_solid3 | w2 | m | Displacement, 3 component | Boundaries 10–14 |  |
| fsi1.ASF | 1 | 1 | Area scaling for offset | Boundaries 10–14 |  |
| fsi1.Tt_stress1 | spf.T_stressx-(spf.T_stressx*spf.nx+spf.T_stressy*spf.ny+spf.T_stressz*spf.nz)*spf.nx | N/m² | Total stress tangential, 1 component | Boundaries 10–14 |  |
| fsi1.Tt_stress2 | spf.T_stressy-(spf.T_stressx*spf.nx+spf.T_stressy*spf.ny+spf.T_stressz*spf.nz)*spf.ny | N/m² | Total stress tangential, 2 component | Boundaries 10–14 |  |
| fsi1.Tt_stress3 | spf.T_stressz-(spf.T_stressx*spf.nx+spf.T_stressy*spf.ny+spf.T_stressz*spf.nz)*spf.nz | N/m² | Total stress tangential, 3 component | Boundaries 10–14 |  |

#### Shape functions

| **Name** | **Shape function** | **Unit** | **Description** | **Shape frame** | **Selection** |
| --- | --- | --- | --- | --- | --- |
| fsi1.vWallx | Lagrange (Linear) | m/s | Wall velocity, x component | Spatial | No boundaries |
| fsi1.vWally | Lagrange (Linear) | m/s | Wall velocity, y component | Spatial | No boundaries |
| fsi1.vWallz | Lagrange (Linear) | m/s | Wall velocity, z component | Spatial | No boundaries |

#### Weak expressions

| **Weak expression** | **Integration order** | **Integration frame** | **Selection** |
| --- | --- | --- | --- |
| (-spf.T_stressx*test(fsi1.u_solid1)-spf.T_stressy*test(fsi1.u_solid2)-spf.T_stressz*test(fsi1.u_solid3))*fsi1.ASF | 4 | Spatial | Boundaries 10–14 |

- 1. Mesh 1

Mesh statistics

| **Description** | **Value** |
| --- | --- |
| Minimum element quality | 0.07182 |
| Average element quality | 0.6423 |
| Tetrahedron | 8645 |
| Pyramid | 340 |
| Hexahedron | 144 |
| Triangle | 1810 |
| Quad | 340 |
| Edge element | 304 |
| Vertex element | 20 |


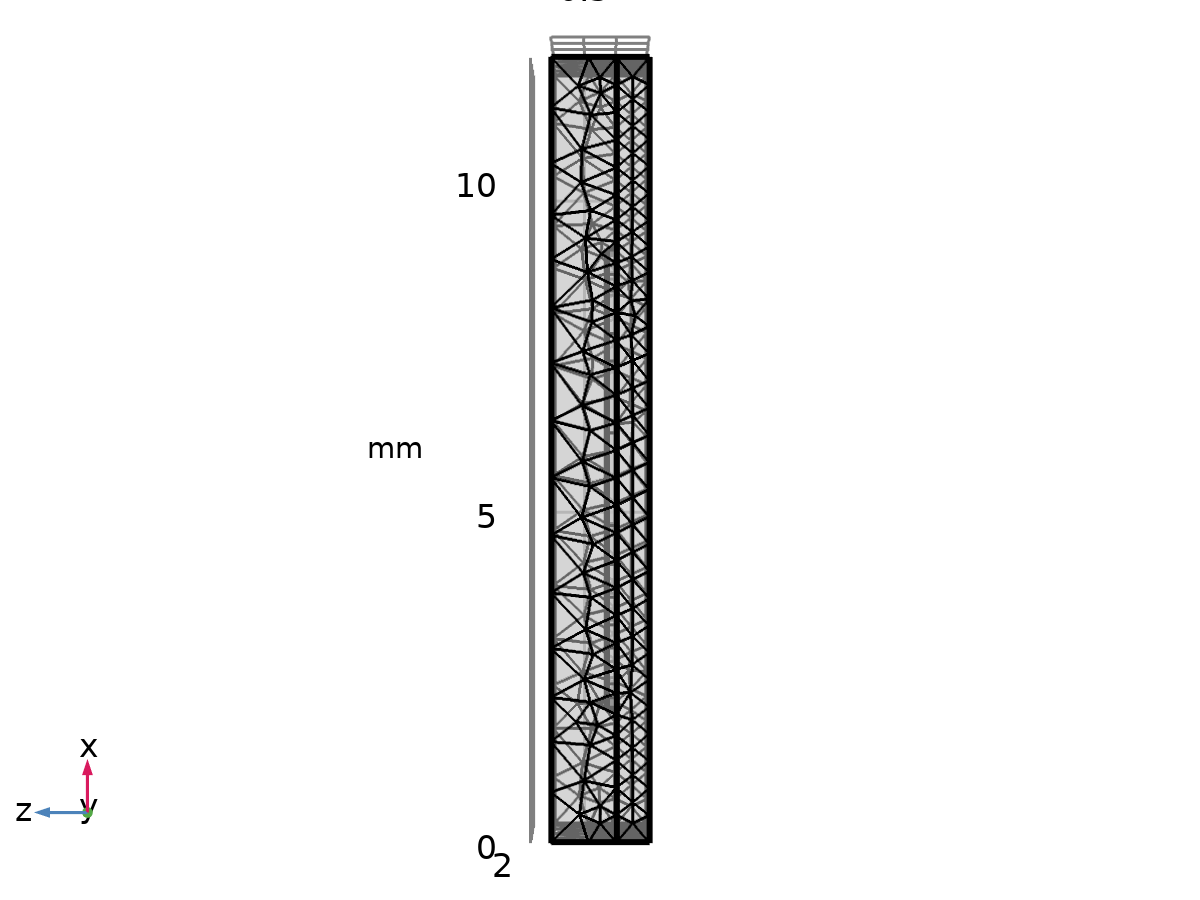


Mesh 1

- - 1. Size (size)

Settings

| **Description** | **Value** |
| --- | --- |
| Maximum element size | 1.8 |
| Minimum element size | 0.336 |
| Curvature factor | 0.7 |
| Resolution of narrow regions | 0.4 |
| Maximum element growth rate | 1.6 |
| Predefined size | Coarse |

- - 1. Corner Refinement 1 (cr1)

Selection

| Geometric entity level | Domain |
| --- | --- |
| Selection | Geometry geom1: Dimension 3: Domain 3 |


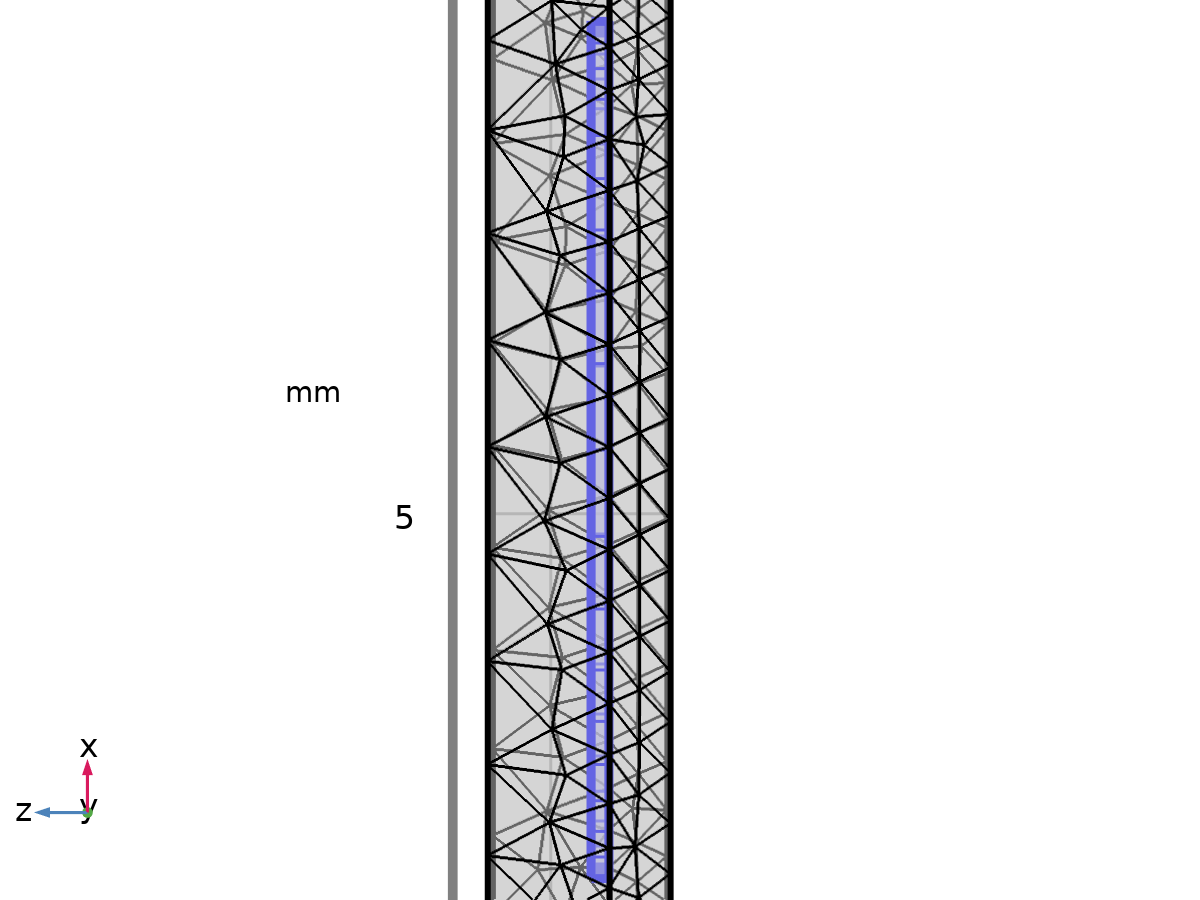


Corner Refinement 1

- - 1. Mapped 1 (map1)

Selection

| Geometric entity level | Boundary |
| --- | --- |
| Selection | Geometry geom1: Dimension 2: Boundary 13 |


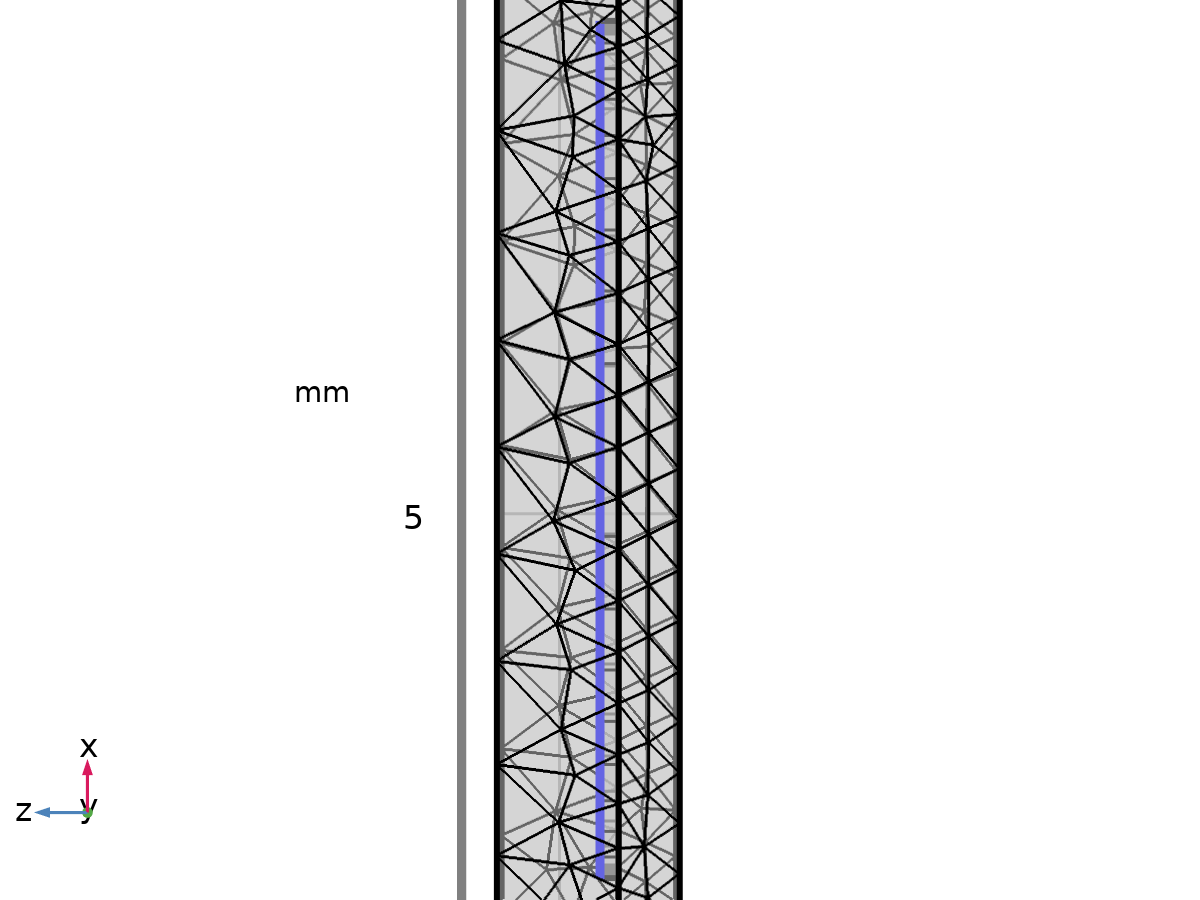


Mapped 1

#### Distribution 2 (dis2)

Selection

| Geometric entity level | Edge |
| --- | --- |
| Selection | Geometry geom1: Dimension 1: Edges 17, 24 |


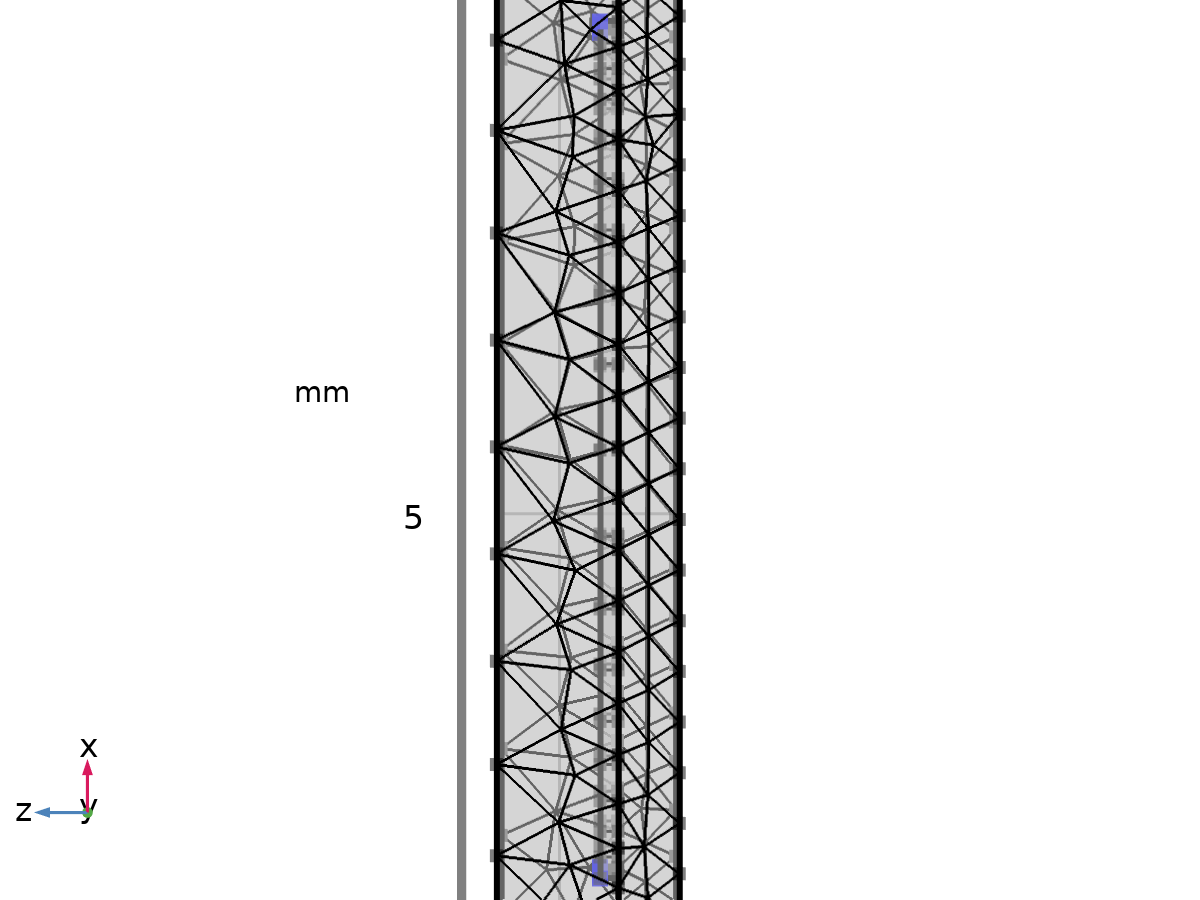


Distribution 2

Settings

| **Description** | **Value** |
| --- | --- |
| Distribution type | Predefined |
| Number of elements | 8 |
| Element ratio | 4 |
| Symmetric distribution | On |

#### Distribution 1 (dis1)

Selection

| Geometric entity level | Edge |
| --- | --- |
| Selection | Geometry geom1: Dimension 1: Edges 18, 21 |


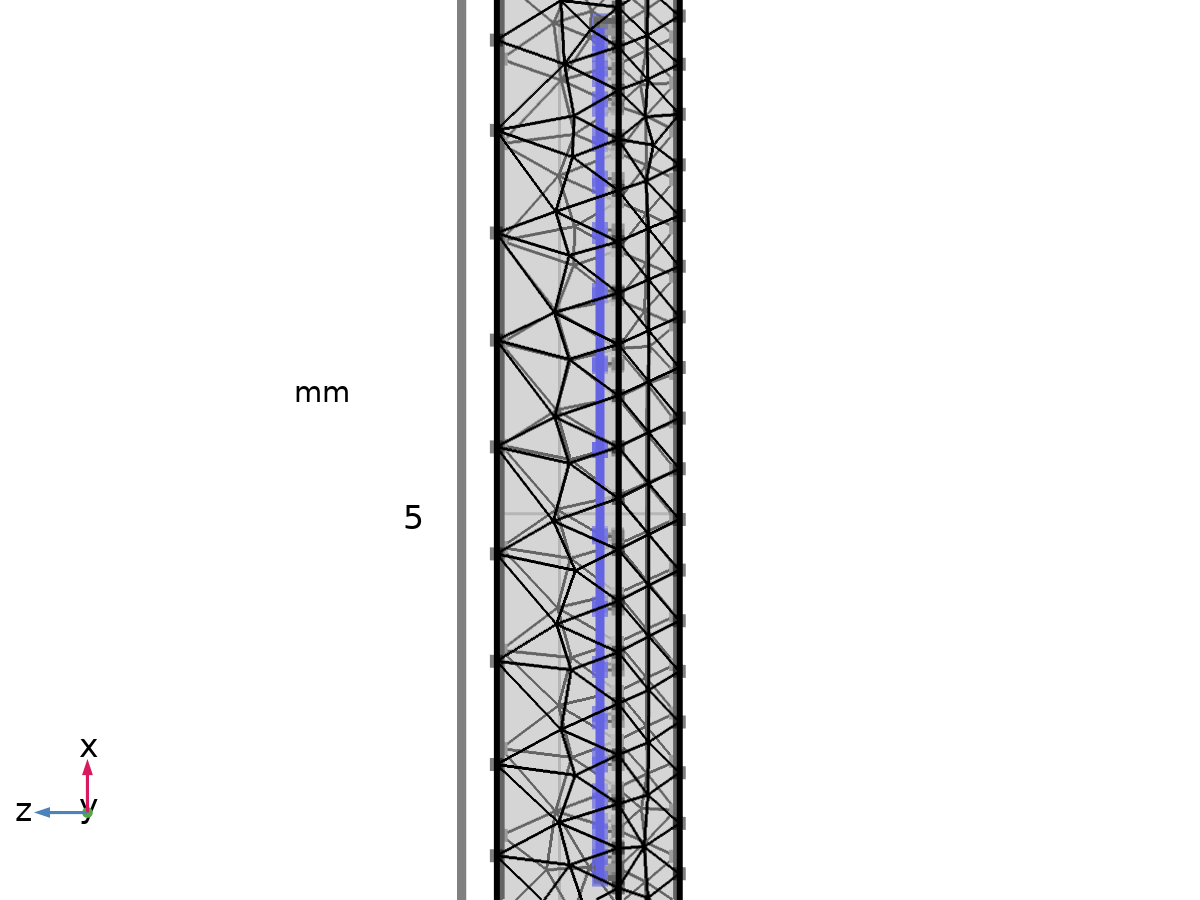


Distribution 1

Settings

| **Description** | **Value** |
| --- | --- |
| Distribution type | Predefined |
| Number of elements | 18 |
| Element ratio | 4 |
| Growth formula | Geometric sequence |
| Symmetric distribution | On |

- - 1. Swept 1 (swe1)

Selection

| Geometric entity level | Domain |
| --- | --- |
| Selection | Geometry geom1: Dimension 3: Domain 3 |


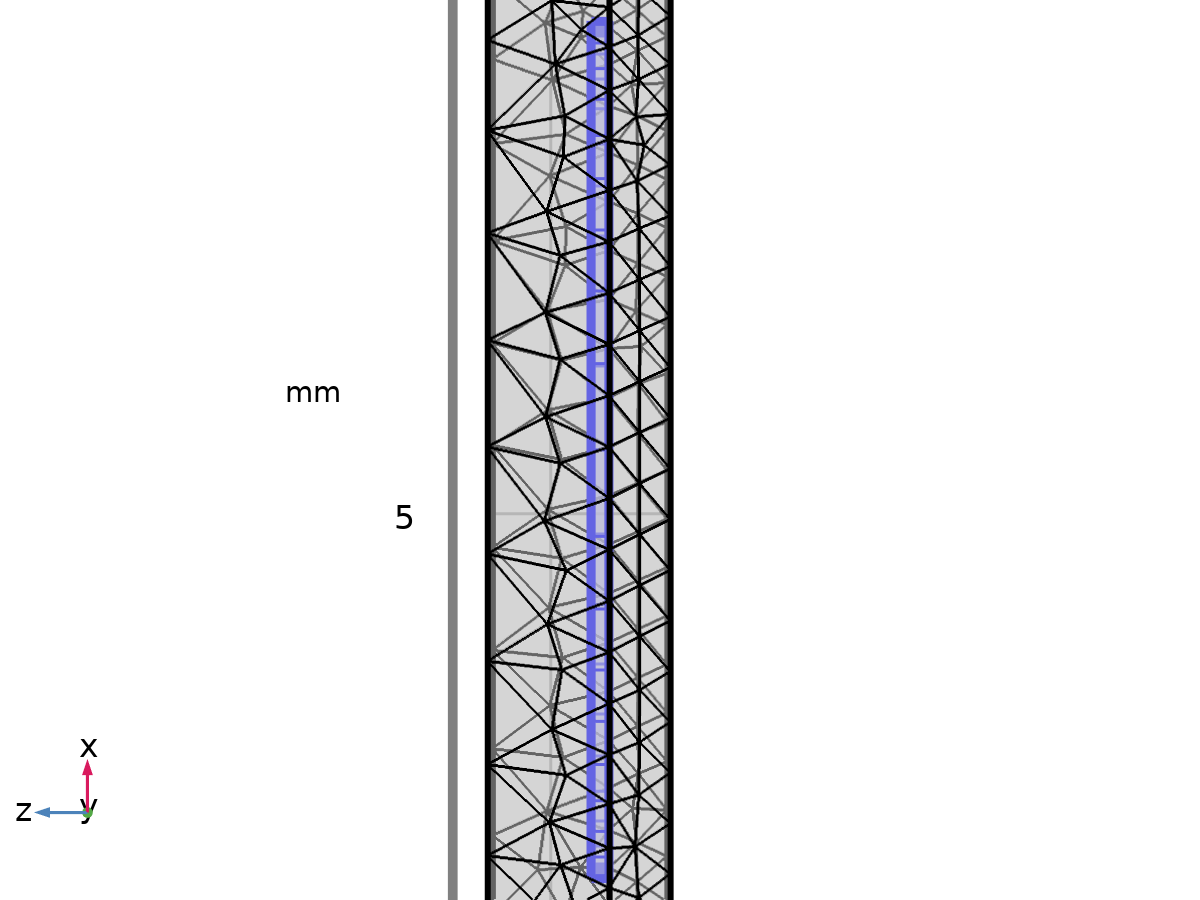


Swept 1

Settings

| **Description** | **Value** |
| --- | --- |
| Face meshing method | Quadrilateral (legacy version 5.4) |

#### Distribution 2 (dis2)

Selection

| Geometric entity level | Domain |
| --- | --- |
| Selection | Geometry geom1: Dimension 3: Domain 3 |


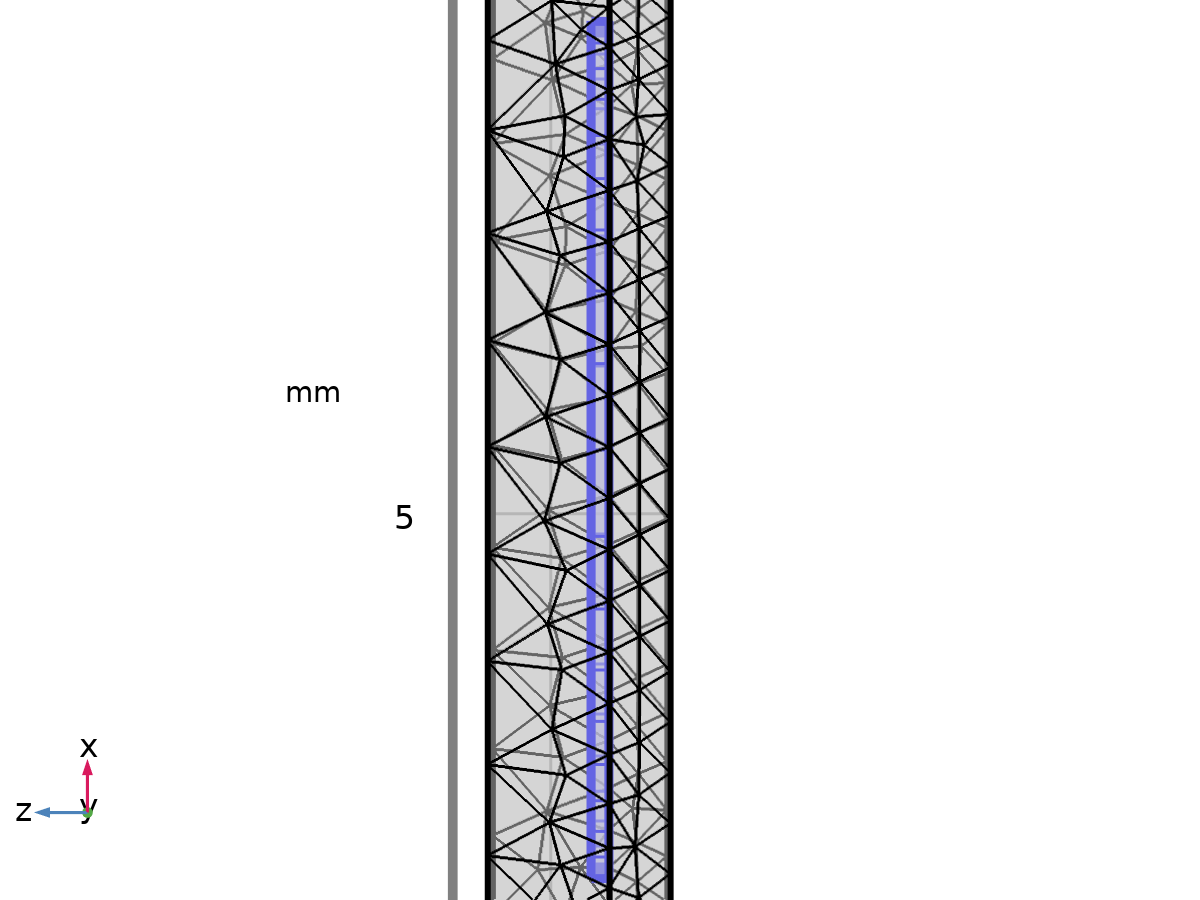


Distribution 2

Settings

| **Description** | **Value** |
| --- | --- |
| Number of elements | 1 |

- - 1. Free Tetrahedral 2 (ftet2)

Selection

| Geometric entity level | Domain |
| --- | --- |
| Selection | Geometry geom1: Dimension 3: Domain 1 |


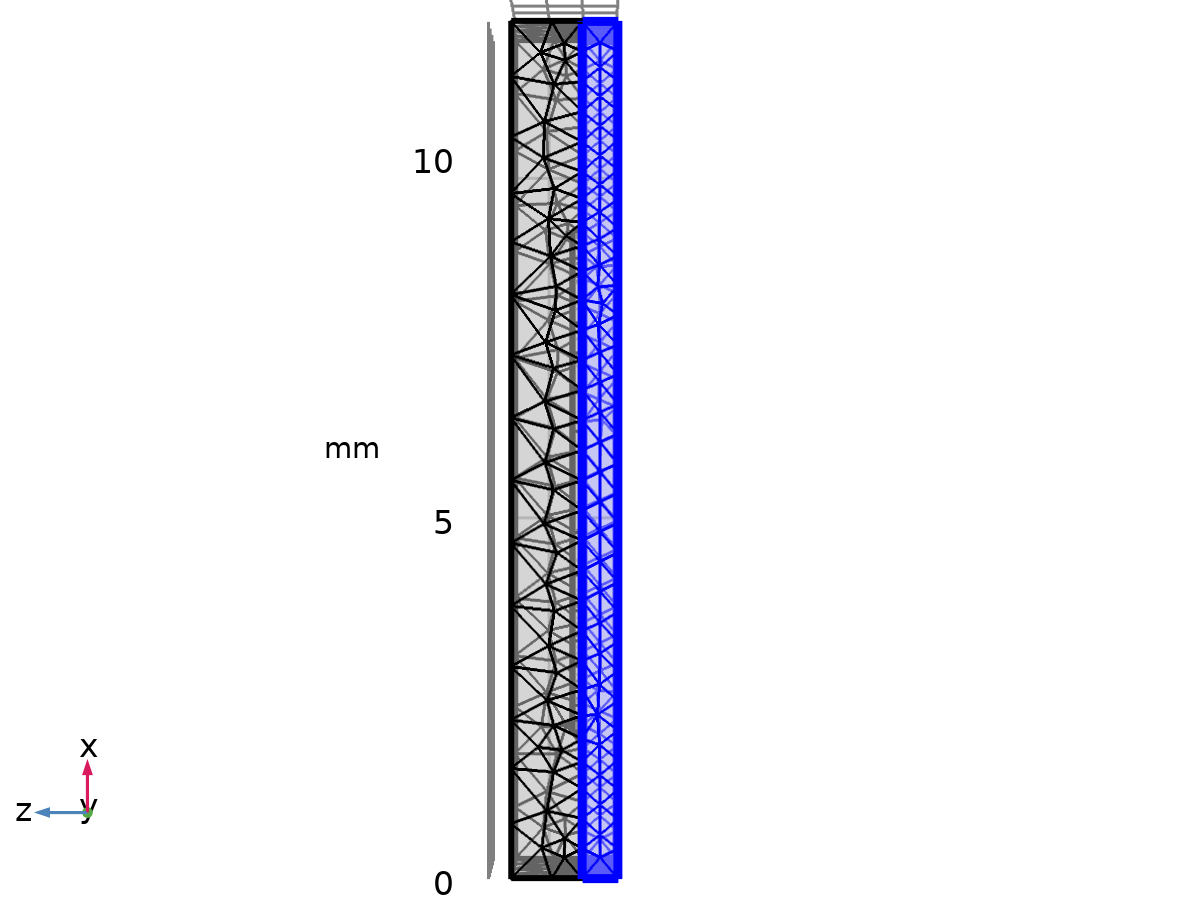


Free Tetrahedral 2

#### Size 1 (size1)

Selection

| Geometric entity level | Domain |
| --- | --- |
| Selection | Geometry geom1: Dimension 3: Domain 1 |


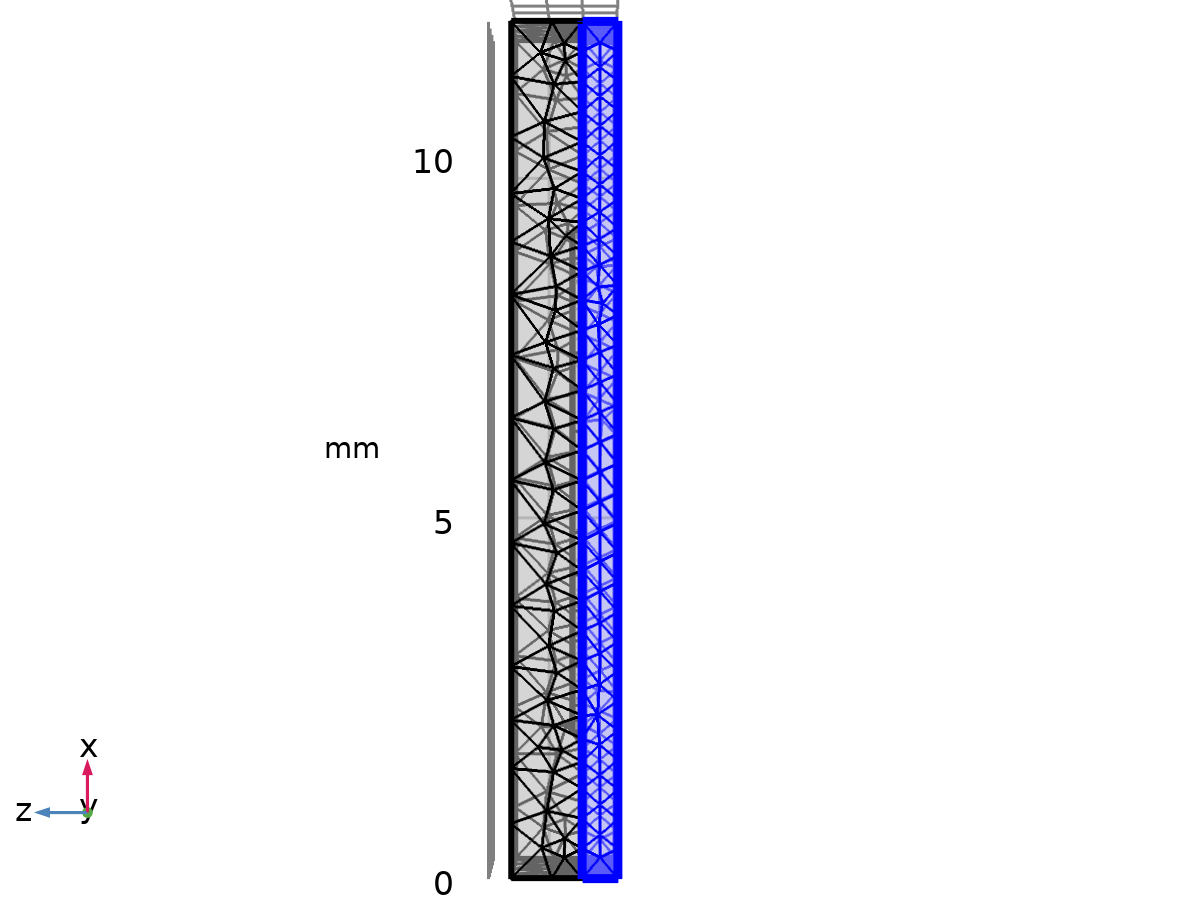


Size 1

Settings

| **Description** | **Value** |
| --- | --- |
| Maximum element size | 0.42 |
| Minimum element size | 0.018 |
| Curvature factor | 0.3 |
| Resolution of narrow regions | 0.85 |
| Maximum element growth rate | 1.35 |
| Predefined size | Extra fine |

- - 1. Free Tetrahedral 3 (ftet3)

Selection

| Geometric entity level | Domain |
| --- | --- |
| Selection | Remaining |


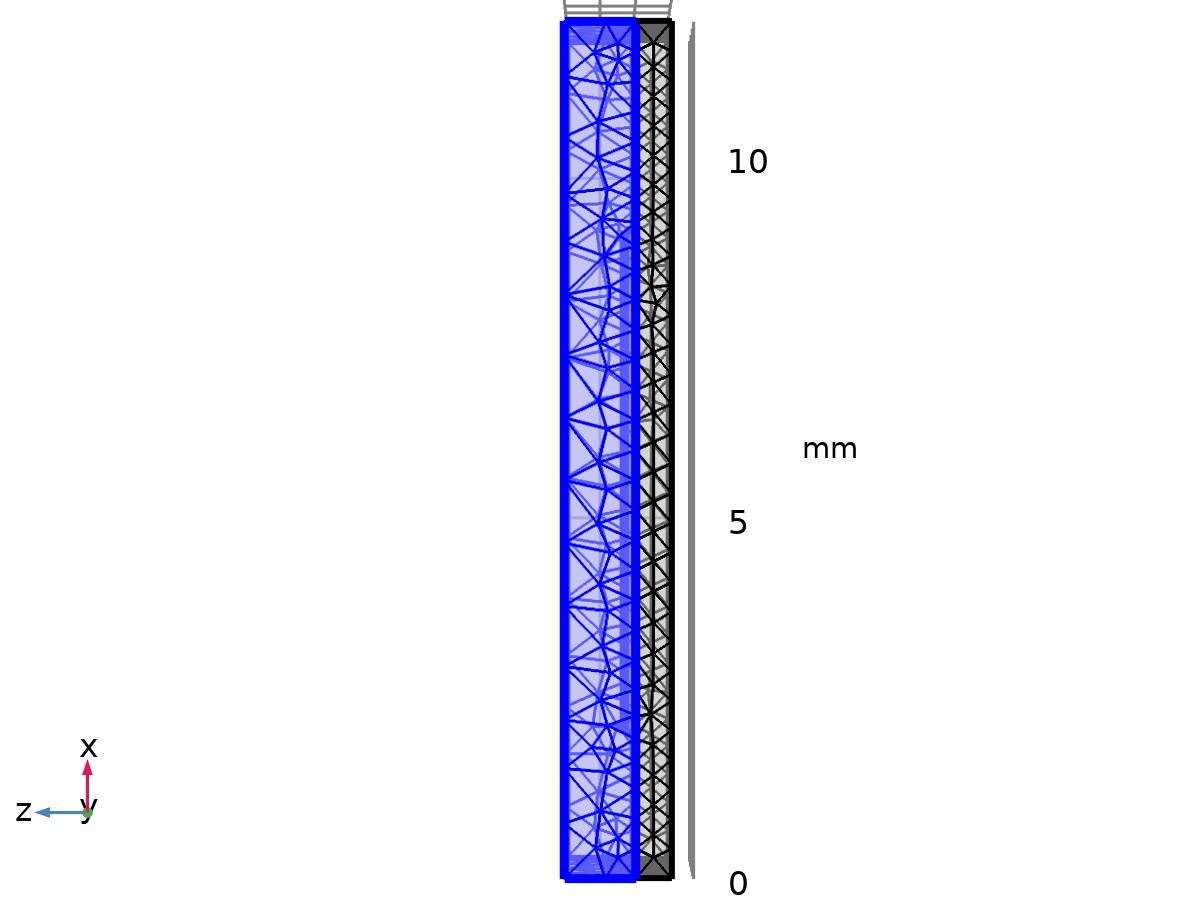


Free Tetrahedral 3

#### Size 1 (size1)

Selection

| Geometric entity level | Domain |
| --- | --- |
| Selection | Geometry geom1 |


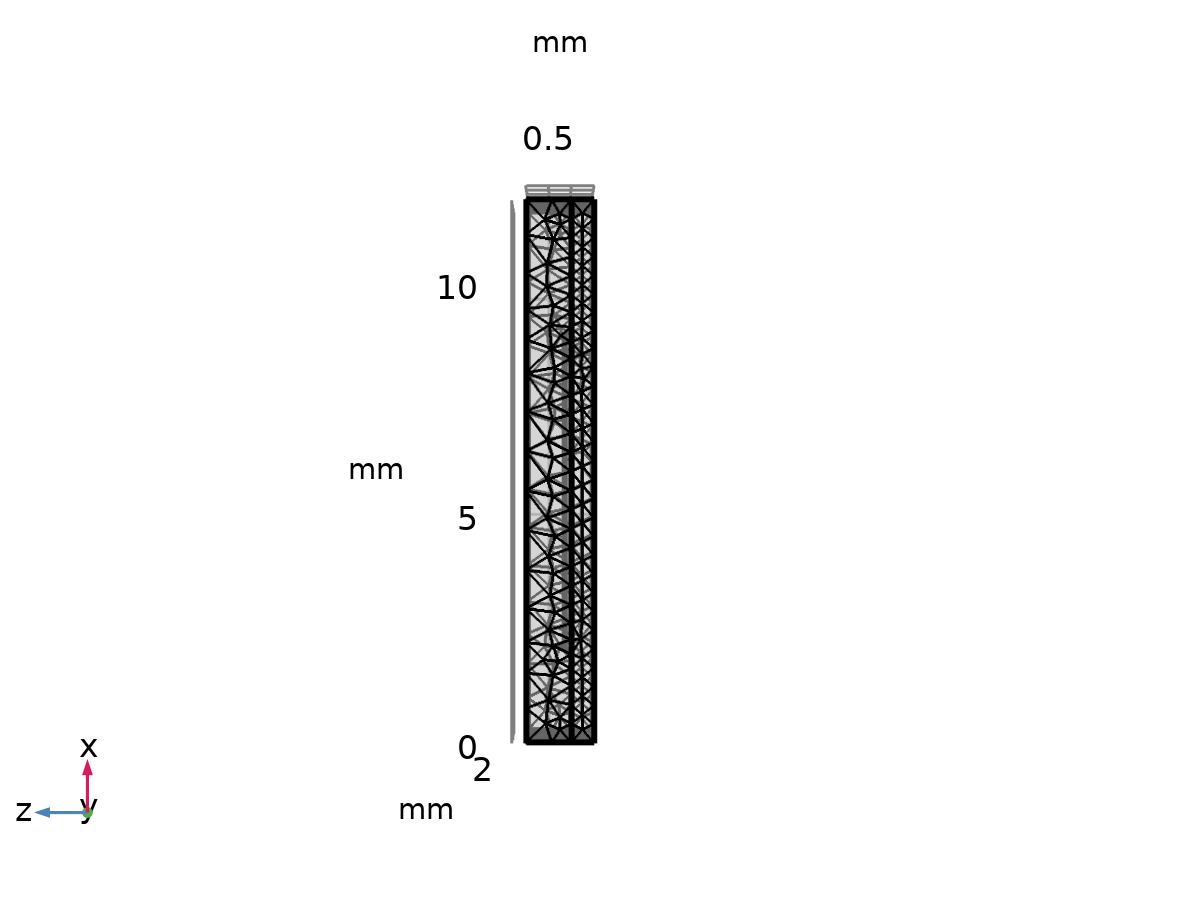


Size 1

Settings

| **Description** | **Value** |
| --- | --- |
| Maximum element size | 1.2 |
| Minimum element size | 0.216 |
| Curvature factor | 0.6 |
| Resolution of narrow regions | 0.5 |
| Maximum element growth rate | 1.5 |

1. Study 1

Computation information

| Computation time | 7 min 38 s |
| --- | --- |
| CPU | Intel64 Family 6 Model 62 Stepping 4, 12 cores |
| Operating system | Windows 10 |

- 1. Time Dependent

| **Times** | **Unit** |
| --- | --- |
| range(0,2,10) | s |

Study settings

| **Description** | **Value** |
| --- | --- |
| Include geometric nonlinearity | On |

Study settings

| **Description** | **Value** |
| --- | --- |
| Times | {0, 2, 4, 6, 8, 10} |

Physics and variables selection

| **Physics interface** | **Discretization** |
| --- | --- |
| Laminar Flow (spf) | physics |
| Solid Mechanics (solid) | physics |

Mesh selection

| **Geometry** | **Mesh** |
| --- | --- |
| Geometry 1 (geom1) | mesh1 |

- 1. Solver Configurations
     1. Solution 1

#### Compile Equations: Time Dependent (st1)

Study and step

| **Description** | **Value** |
| --- | --- |
| Use study | [Study 1](#cs9313594) |
| Use study step | [Time Dependent](#cs8894568) |

Log

<---- Compile Equations: Time Dependent in Study 1/Solution 1 (sol1) -----------

Started at Aug 6, 2022 8:09:11 AM.

Geometry shape order: Linear

Running on Intel64 Family 6 Model 62 Stepping 4, GenuineIntel.

Using 1 socket with 12 cores in total on ORTHO-VARLAB1.

Available memory: 32.71 GB.

Time: 3 s.

Physical memory: 2.94 GB

Virtual memory: 3.45 GB

Ended at Aug 6, 2022 8:09:14 AM.

----- Compile Equations: Time Dependent in Study 1/Solution 1 (sol1) ---------->

#### Dependent Variables 1 (v1)

General

| **Description** | **Value** |
| --- | --- |
| Defined by study step | [Time Dependent](#cs8894568) |

Initial value calculation constants

| **Constant name** | **Initial value source** |
| --- | --- |
| t | range(0,2,10) |
| timestep | 0.01[s] |

Log

<---- Dependent Variables 1 in Study 1/Solution 1 (sol1) -----------------------

Started at Aug 6, 2022 8:09:14 AM.

Solution time: 0 s.

Physical memory: 2.94 GB

Virtual memory: 3.44 GB

Ended at Aug 6, 2022 8:09:14 AM.

----- Dependent Variables 1 in Study 1/Solution 1 (sol1) ---------------------->

##### Auxiliary pressure (comp1.solid.pw) (comp1_solid_pw)

General

| **Description** | **Value** |
| --- | --- |
| Field components | comp1.solid.pw |

Scaling

| **Description** | **Value** |
| --- | --- |
| Method | Manual |
| Scale | 100000000 |

##### Spatial mesh displacement (comp1.spatial.disp) (comp1_spatial_disp)

General

| **Description** | **Value** |
| --- | --- |
| Field components | {comp1.spatial.u, comp1.spatial.v, comp1.spatial.w} |

Scaling

| **Description** | **Value** |
| --- | --- |
| Method | Manual |
| Scale | 3.282226160766679E-5 |

##### Velocity field (spatial frame) (comp1.u) (comp1_u)

General

| **Description** | **Value** |
| --- | --- |
| Field components | {comp1.u, comp1.v, comp1.w} |
| Internal variables | {comp1.spf.isFluidHasBeenSolved, comp1.spf.dt2Inv_u} |

##### Pressure (comp1.p) (comp1_p)

General

| **Description** | **Value** |
| --- | --- |
| Field components | comp1.p |

##### Displacement field (comp1.u2) (comp1_u2)

General

| **Description** | **Value** |
| --- | --- |
| Field components | {comp1.u2, comp1.v2, comp1.w2} |

Scaling

| **Description** | **Value** |
| --- | --- |
| Method | Manual |
| Scale | 1e-2*0.012581335382223939 |

##### Wall velocity (spatial frame) (comp1.fsi1.vWall) (comp1_fsi1_vWall)

General

| **Description** | **Value** |
| --- | --- |
| Field components | {comp1.fsi1.vWallx, comp1.fsi1.vWally, comp1.fsi1.vWallz} |

#### Time-Dependent Solver 1 (t1)

General

| **Description** | **Value** |
| --- | --- |
| Defined by study step | [Time Dependent](#cs8894568) |
| Times | {0, 2, 4, 6, 8, 10} |
| Relative tolerance | 0.005 |

Field tolerance method

| **Field** | **Value** |
| --- | --- |
| Auxiliary pressure (comp1.solid.pw) | Use_global |
| Spatial mesh displacement (comp1.spatial.disp) | Use_global |
| Velocity field (spatial frame) (comp1.u) | Use_global |
| Pressure (comp1.p) | Scaled |
| Displacement field (comp1.u2) | Use_global |
| Wall velocity (spatial frame) (comp1.fsi1.vWall) | Use_global |

Field tolerance factor

| **Field** | **Value** |
| --- | --- |
| Auxiliary pressure (comp1.solid.pw) | 0.1 |
| Spatial mesh displacement (comp1.spatial.disp) | 0.1 |
| Velocity field (spatial frame) (comp1.u) | 0.1 |
| Pressure (comp1.p) | 1 |
| Displacement field (comp1.u2) | 0.1 |
| Wall velocity (spatial frame) (comp1.fsi1.vWall) | 0.1 |

Time stepping

| **Description** | **Value** |
| --- | --- |
| Maximum BDF order | 2 |
| Nonlinear controller | On |
| Fraction of initial step for Backward Euler | 0.01 |
| Error estimation | Exclude algebraic |

Log

<---- Time-Dependent Solver 1 in Study 1/Solution 1 (sol1) ---------------------

Started at Aug 6, 2022 8:09:14 AM.

Time-dependent solver (BDF)

Number of degrees of freedom solved for: 47951 (plus 145 internal DOFs).

Nonsymmetric matrix found.

Scales for dependent variables:

Pressure (comp1.p): 2.1

Auxiliary pressure (comp1.solid.pw): 1e+08

Spatial mesh displacement (comp1.spatial.disp): 3.3e-05

Velocity field (spatial frame) (comp1.u): 3.5

Displacement field (comp1.u2): 0.00013

Step        Time    Stepsize      Res  Jac  Sol Order Tfail NLfail   LinErr   LinRes

   0           0           - out   11    3   11                  0  2.4e-15  1.7e-15

   1  0.00086104  0.00086104       14    4   14     1     0      0  5.6e-15  1.2e-15

   2   0.0017221  0.00086104       17    5   17     1     0      0  4.2e-14  2.4e-15

   3   0.0034442   0.0017221       20    6   20     2     0      0  1.9e-14  4.5e-15

   4   0.0068884   0.0034442       23    7   23     1     0      0  4.4e-15  6.4e-15

   5    0.010333   0.0034442       26    8   26     1     0      0  8.8e-15  2.7e-14

   6    0.017221   0.0068884       29    9   29     1     0      0  4.2e-14  6.2e-14

   7    0.024109   0.0068884       32   10   32     1     0      0  1.5e-13  7.4e-14

   8    0.030998   0.0068884       34   11   34     1     0      0    5e-13  8.3e-14

   9    0.044774    0.013777       37   12   37     1     0      0  6.2e-14  9.5e-14

  10    0.058551    0.013777       40   13   40     1     0      0  1.4e-13  9.9e-14

  11    0.072328    0.013777       43   14   43     1     0      0  3.7e-13  5.6e-14

  12    0.099881    0.027553       46   15   46     2     0      0  1.5e-13  4.7e-14

  13     0.12743    0.027553       49   16   49     1     0      0  4.5e-13  2.8e-14

  14     0.18254    0.055107       52   17   52     1     0      0    2e-13  1.3e-14

  15     0.23765    0.055107       55   18   55     1     0      0  5.1e-13  1.9e-14

  16     0.29275    0.055107       58   19   58     1     0      0  8.3e-13  1.4e-14

  17     0.40297     0.11021       61   20   61     2     0      0  2.7e-13  5.4e-15

  18     0.51318     0.11021       64   21   64     2     0      0  2.6e-13  3.3e-15

  19     0.73361     0.22043       68   22   68     2     0      0  1.4e-12  5.4e-15

  20     0.95404     0.22043       72   23   72     2     0      0  2.9e-13  6.5e-15

  21      1.1745     0.22043       76   24   76     2     0      0  1.9e-12    3e-15

  22      1.3949     0.22043       81   25   81     2     0      0  1.3e-12  3.4e-15

  23      1.6153     0.22043       86   26   86     2     0      0  7.4e-13  2.3e-15

  24      1.8357     0.22043       92   27   92     2     0      0  6.8e-12    2e-15

   -           2           - out

  25      2.0562     0.22043       98   28   98     2     0      0  5.6e-13  1.9e-15

  26      2.2766     0.22043      104   29  104     2     0      0  1.5e-12  1.8e-15

  27       2.497     0.22043      110   30  110     2     0      0  1.3e-12  2.6e-15

  28      2.7175     0.22043      116   31  116     2     0      0  3.5e-12    5e-15

  29      2.9379     0.22043      122   32  122     2     0      0  1.1e-12  1.2e-14

  30      3.1583     0.22043      128   33  128     2     0      0  5.2e-12    1e-14

  31      3.5992     0.44085      135   34  135     2     0      0  2.5e-12  4.2e-15

   -           4           - out

  32        4.04     0.44085      142   35  142     2     0      0  1.2e-12  4.3e-15

  33      4.4809     0.44085      149   36  149     2     0      0  7.3e-13  2.4e-15

  34      4.9217     0.44085      156   37  156     2     0      0  2.3e-12  1.2e-14

  35      5.3626     0.44085      163   38  163     2     0      0  3.3e-12  7.2e-15

  36      5.8034     0.44085      170   39  170     2     0      0  5.5e-12  1.7e-14

   -           6           - out

  37      6.2443     0.44085      178   40  178     2     0      0  3.1e-12    1e-14

  38      6.6851     0.44085      186   41  186     2     0      0    4e-12  9.6e-15

  39       7.126     0.44085      194   42  194     2     0      0  3.3e-12  8.5e-15

  40      7.5669     0.44085      202   43  202     2     0      0  8.4e-13  4.9e-15

  41      7.9504     0.38354      226   46  226     2     0      2  7.8e-13    4e-14

   -           8           - out

  42      8.0463    0.095886      250   49  250     2     0      4    4e-12  1.8e-14

  43      8.2381     0.19177      258   50  258     2     0      4  5.3e-12  2.7e-14

  44      8.4682     0.23013      274   52  274     2     0      5  5.7e-13  1.4e-14

  45      8.5182    0.050052      306   56  306     2     0      8  7.1e-13  8.9e-15

  46      8.5683    0.050052      313   57  313     2     0      8  8.6e-13  5.6e-15

  47      8.6183    0.050052      319   58  319     2     0      8  7.5e-12  2.3e-14

  48      8.6684    0.050052      326   59  326     2     0      8  2.9e-12  1.5e-14

  49      8.7184    0.050052      333   60  333     2     0      8  7.4e-13  1.7e-14

  50      8.7685    0.050052      340   61  340     2     0      8  7.3e-13  1.4e-14

  51      8.8185    0.050052      347   62  347     2     0      8  3.5e-12  2.3e-14

  52      8.8686    0.050052      354   63  354     2     0      8  5.3e-13  9.6e-15

  53      8.9187    0.050052      361   64  361     2     0      8  3.1e-12  8.2e-14

  54      8.9687    0.050052      368   65  368     2     0      8  6.6e-12  1.4e-14

  55      9.0188    0.050052      375   66  375     2     0      8  9.9e-13  9.5e-15

  56      9.1189      0.1001      382   67  382     2     0      8  1.1e-12  2.9e-14

  57      9.3191     0.20021      390   68  390     2     0      8  5.9e-12  1.7e-13

  58      9.3626    0.043546      422   72  422     2     0     11    7e-13  2.5e-14

  59      9.4062    0.043546      430   73  430     2     0     11  3.9e-12    5e-13

  60      9.4497    0.043546      437   74  437     2     0     11  2.6e-12  9.9e-15

  61      9.4933    0.043546      444   75  444     2     0     11  3.3e-12  2.6e-14

  62      9.5368    0.043546      451   76  451     2     0     11  6.6e-12  4.9e-14

  63      9.5803    0.043546      458   77  458     2     0     11  7.4e-12  6.1e-13

  64      9.6239    0.043546      465   78  465     2     0     11  1.5e-12  6.4e-14

  65      9.6674    0.043546      472   79  472     2     0     11    7e-12  2.3e-14

  66       9.711    0.043546      479   80  479     2     0     11  1.6e-12  2.8e-14

  67      9.7545    0.043546      486   81  486     2     0     11  7.4e-13  8.2e-14

  68      9.7981    0.043546      489   82  489     2     0     11  2.4e-12  4.3e-14

  69      9.8852    0.087091      497   83  497     2     0     11  8.9e-12  1.6e-13

  70      9.9041    0.018942      527   87  527     2     0     14  8.9e-13  1.1e-14

  71       9.923    0.018942      533   88  533     2     0     14  2.6e-12  3.6e-13

  72       9.942    0.018942      538   89  538     2     0     14  1.7e-12  7.3e-14

  73      9.9609    0.018942      544   90  544     2     0     14    1e-12  1.9e-13

  74      9.9799    0.018942      549   91  549     2     0     14  8.8e-13  3.4e-14

  75      9.9988    0.018942      555   92  555     2     0     14  6.9e-13  1.7e-14

  76          10   0.0011812 out  558   93  558     2     0     14  2.3e-14  1.2e-14

Time-stepping completed.

Solution time: 455 s. (7 minutes, 35 seconds)

Physical memory: 4.25 GB

Virtual memory: 4.89 GB

Ended at Aug 6, 2022 8:16:49 AM.

----- Time-Dependent Solver 1 in Study 1/Solution 1 (sol1) -------------------->

##### Direct (dDef)

General

| **Description** | **Value** |
| --- | --- |
| Solver | PARDISO |

##### Previous Solution 1 (ps1)

Previous solution

| **Description** | **Value** |
| --- | --- |
| Linear solver | [Direct](#cs3987141) |
| Damping factor | 0.35 |
| Damping factor | On |

##### Fully Coupled 1 (fc1)

General

| **Description** | **Value** |
| --- | --- |
| Linear solver | [Direct](#cs3987141) |

Method and termination

| **Description** | **Value** |
| --- | --- |
| Damping factor | 0.9 |
| Jacobian update | Once per time step |
| Maximum number of iterations | 8 |
| Stabilization and acceleration | Anderson acceleration |
| Dimension of iteration space | 5 |

1. Results
   1. Data Sets
      1. Study 1/Solution 1

Solution

| **Description** | **Value** |
| --- | --- |
| Solution | [Solution 1](#cs8945896) |
| Component | Save Point Geometry 1 |


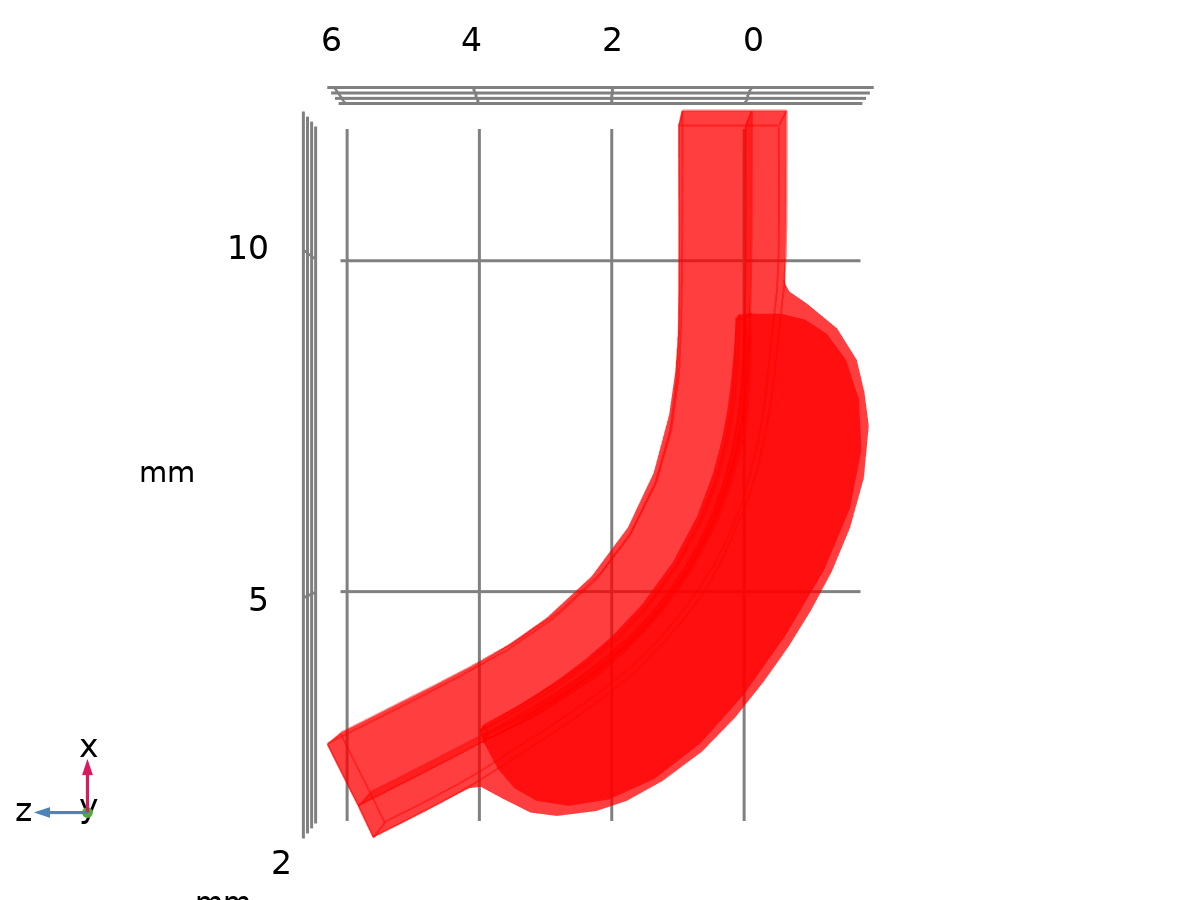


Dataset: Study 1/Solution 1

- - 1. Exterior Walls

Data

| **Description** | **Value** |
| --- | --- |
| Dataset | [Study 1/Solution 1](#cs1818620) |

Parameterization

| **Description** | **Value** |
| --- | --- |
| x- and y-axes | Surface parameters |

- 1. Plot Groups
     1. Pressure (spf)


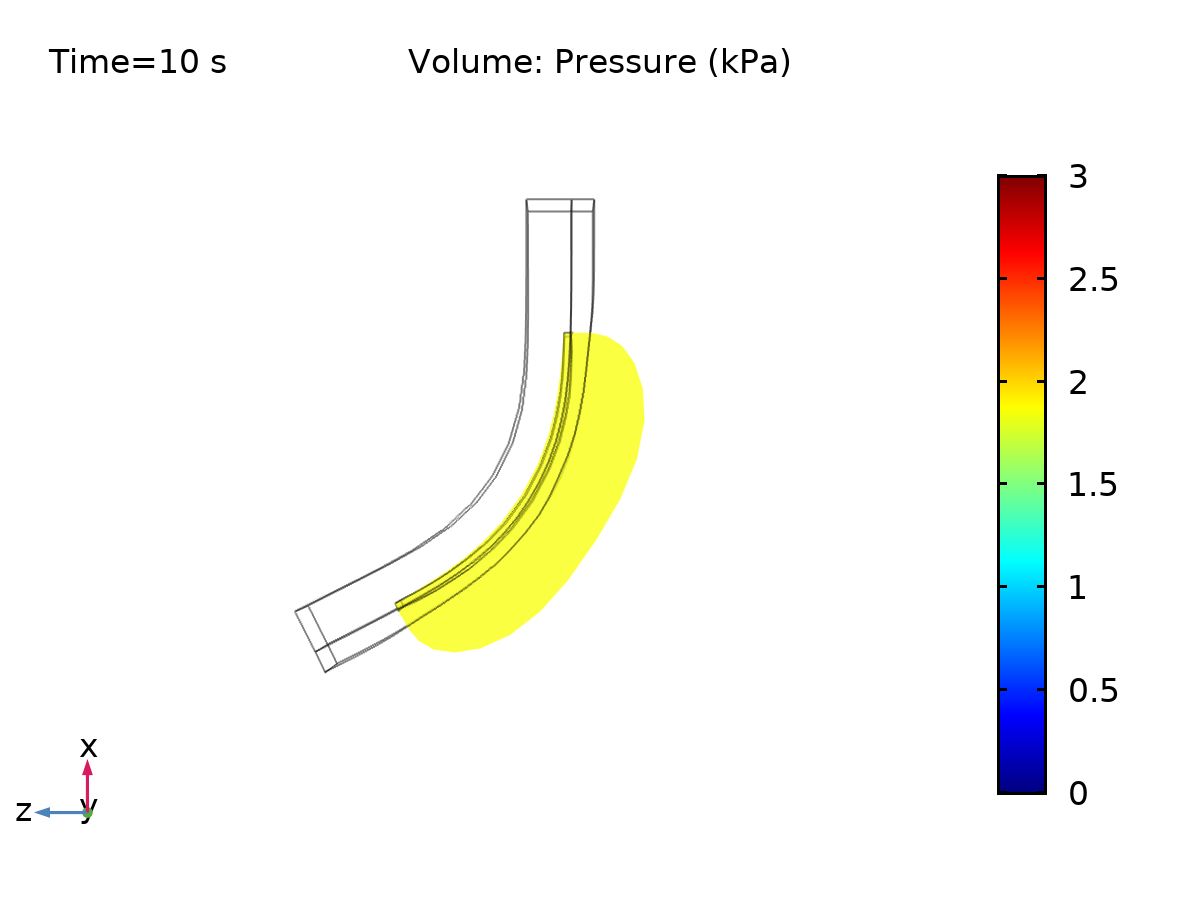


Volume: Pressure (kPa)

- - 1. 3D Plot Group 4


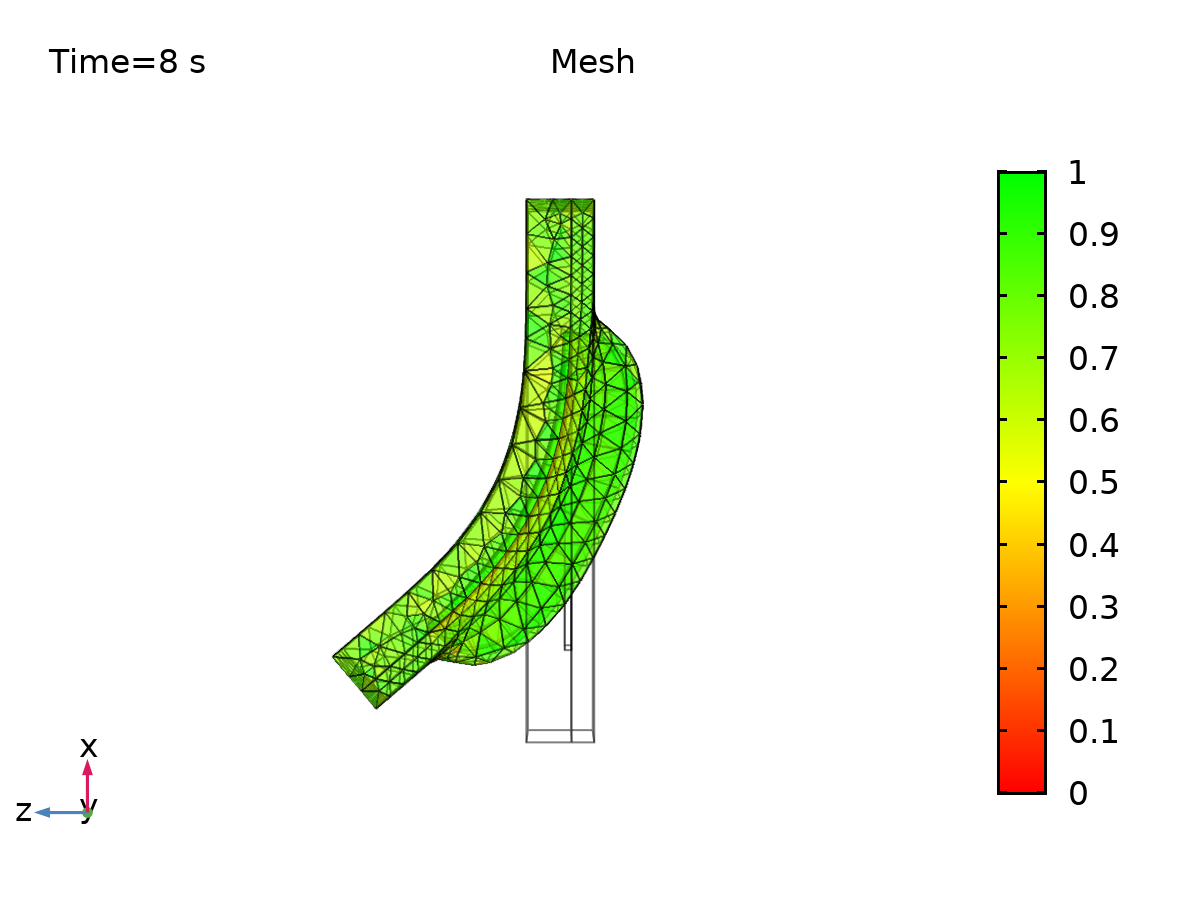


Mesh
